# The mechanism study of quercetin isolated from *Zanthoxylum bungeanum* maxim. inhibiting ferroptosis and alleviating MAFLD through p38 MAPK/ERK signaling pathway based on lipidomics and transcriptomics

**DOI:** 10.3389/fphar.2025.1517291

**Published:** 2025-03-31

**Authors:** Yan Chen, Fajian Ren, Nannan Yang, Qiwen Xiang, Song Gao, Wei Pu, Zhou Yang, Qiuyan Liu, Shajie Luo, Chaolong Rao

**Affiliations:** ^1^ School of Public Health, Chengdu University of Traditional Chinese Medicine, Chengdu, Sichuan, China; ^2^ College of Medical Technology, Chengdu University of Traditional Chinese Medicine, Chengdu, Sichuan, China; ^3^ Key Laboratory of Southwestern Chinese Medicine Resources, Chengdu University of Traditional Chinese Medicine, Chengdu, Sichuan, China

**Keywords:** *Zanthoxylum bungeanum* maxim., metabolic-associated fatty liver disease, quercetin, lipidomics, transcriptomics

## Abstract

**Background:**

As a resource with a variety of medicinal and edible values, *Zanthoxylum bungeanum* Maxim has been found to improve high-fat diet-induced metabolic-associated fatty liver disease (MAFLD).

**Aim of the study:**

The aim of this study was to predict the main active metabolites in *Z. bungeanum* Maxim. Based on network analysis, and to explore and validate their potential mechanisms of action through lipidomics and transcriptomic techniques.

**Materials and Methods:**

MAFLD mouse model and cell model were established to evaluate the effect of active components in *Z. bungeanum* Maxim. on MAFLD. Serum biochemical indexes, pathological staining observation, lipid group and transcriptome were used to verify the mechanism of action of active components in *Z. bungeanum* Maxim. on MAFLD.

**Results:**

Quercetin can regulate the liver lipid metabolites of MAFLD mice through the Glycerophospholipid metabolic pathway, thereby improving liver lipid accumulation and liver injury. At the same time, quercetin can also improve MAFLD by reducing oleic acid-induced lipid accumulation in HepG2 cells, and inhibit ferroptosis through the p38 MAPK/ERK signaling pathway, thereby alleviating the progression of MAFLD.

**Conclusion:**

Quercetin isolated from *Z. bungeanum* Maxim. has ameliorative effects on MAFLD, probably mainly by affecting lipid metabolic pathways and MAPK signaling pathways.

## 1 Introduction

Metabolically associated fatty liver disease (MAFLD) is a new definition proposed by an international panel of experts based on non-alcoholic fatty liver disease (NAFLD), a type of metabolic stress-related liver damage strongly associated with insulin resistance and genetic predisposition (Cai et al., 2025). MAFLD is a common chronic metabolic disease with a global prevalence of 25%–30% (Shi et al., 2025). According to the severity of the disease and symptoms, MAFLD can divided into simple fatty liver disease, non-alcoholic steatohepatitis, liver fibrosis, cirrhosis, and hepatocellular carcinoma (Mansour et al., 2025). The pathogenesis of MAFLD is related to many factors such as diet, obesity, insulin resistance, inflammatory factors and adipose tissue dysfunction (Wan et al., 2025). Due to its complex pathogenesis, the research and development of drugs for the treatment of MAFLD has always been a great challenge. Currently, there is still a lack of effective therapeutic medications. Therefore, it is urgently needed to explore the pathological mechanisms of MAFLD and search for new treatment drugs and targets in order to prevent and slow down the progression of MAFLD at an early stage.

Natural extracts are the main way of drug development, accounting for 30% of clinical drugs for the treatment of diseases (Cai et al., 2025). Due to their rich pharmacological activity and low side effects, natural extracts have been widely used to treat MAFLD (Ma et al., 2023). In the early stage of MAFLD, dietary control and treatment can be utilized for management. Therefore, natural extracts, as a valuable resource, hold significant importance in the prevention and treatment of MAFLD. *Zanthoxylum bungeanum* Maxim. Is a plant of the genus Zanthoxylum in the rutaceae family, which is widely distributed in Japan, India, Korea, China and other places (Zhang T. et al., 2024). Modern scientific research found that *Z. bungeanum* Maxim. Contains rich chemical components, mainly including volatile oil, alkaloids, flavonoids and free fatty acids, with analgesic, anti-inflammatory, antibacterial, antioxidant, anti-tumor and other extensive biological activities (Li et al., 2025). In traditional folk medicine, the peel, stem and seeds of *Z. bungeanum* Maxim. Have been used to treat tuberculosis, malaria, tonsillitis, arthritis, fever and abdominal pain (Yuan et al., 2021; Ye et al., 2023). Studies have found that *Z. bungeanum* Maxim. Can improve high-fat diet-induced MAFLD by regulating fatty acid and cholesterol metabolism, intestinal microflora and metabolic characteristics (Huang X. et al., 2023). However, the specific components of *Z. bungeanum* Maxim. In the treatment of MAFLD are still unclear. Therefore, we further explored the therapeutic effect of active components in *Z. bungeanum* Maxim. On MAFLD. Quercetin is a flavonoid substance in *Z. bungeanum* Maxim., which is widely found in various plants and foods in daily life. Studies have found that quercetin can promote insulin secretion, improve insulin resistance, reduce blood lipid levels, inhibit inflammation and oxidative stress, alleviate liver lipid accumulation, and regulate intestinal microflora disorders to improve MAFLD (Katsaros et al., 2024; Markowska et al., 2024). Therefore, we speculated that quercetin may be an important active ingredient of *Z. bungeanum* Maxim. In the treatment of MAFLD.

As a resource with a variety of medicinal and edible values, the effect of *Z. bungeanum* Maxim. On MAFLD deserves further discussion. In recent years, the development of emerging technologies such as network analysis and molecular docking has shown great potential in revealing the active metabolites of *Z. bungeanum* Maxim. For the treatment of MAFLD and their mechanisms of action (Chandran et al., 2017; Dong et al., 2021). Therefore, the purpose of this study was to screen the main active components and potential mechanisms of *Z. bungeanum* Maxim. In the treatment of MAFLD by network analysis and molecular docking. MAFLD mouse model and cell model were established to evaluate the effect of active components in *Z. bungeanum* Maxim. On MAFLD. Serum biochemical indexes, pathological staining observation, lipid group and transcriptome were used to verify the mechanism of action of active components in *Z. bungeanum* Maxim. On MAFLD, so as to provide theoretical reference for clinical application of *Z. bungeanum* Maxim. In the treatment of MAFLD.

## 2 Materials and Methods

### 2.1 Reagents and antibodies

Quercetin (≥98% purity, SQ8030/(218P021)) purchased from Beijing Solarbio Technology Co. Ltd (Beijing, China). Tetracycline (20220225) purchased from National Pharmaceutical Group Chemical Reagents Co., Ltd (Shanghai, China). Oleic acid (#0000115688), purchased from Sigma Co., Ltd (St Louis, MO, United States). CCK-8 Kit (BS350B) purchased from Biosharp Biotechnology Co., Ltd. (Hefei, China). Aspartate aminotransferase (ALT), alanine aminotransferase (AST), LDL-C, HDL-C, TC and TG assay kits were obtained from Nanjing Jiancheng Biological Engineering (Nanjing, China). Paraformaldehyde 4% solution and hematoxylin and eosin dye were obtained from Biosharp Technology Co., Ltd (Anhui, China). GAPDH was obtained from Servicebio Biotechnology Co., Ltd. (Wuhan, China). Primary antibodies against P-p38 MAPK and p38 MAPK were acquired from Cell Signaling Tech (Danvers, MA, United States). Primary antibodies against p-ERK1/2, ERK1/2, xCT and GPX4 were acquired from Abmart Shanghai Co., Ltd. (Shanghai, China). Primary antibodies against SLC3A2/CD98hc were acquired from ABclonal Technology Co., Ltd. (Wuhan, China).

### 2.2 Network analysis

#### 2.2.1 Screening the active metabolites and target genes of *Zanthoxylum bungeanum* maxim

The active metabolites in *Z. bungeanum* Maxim. Were searched by using Traditional Chinese Medicine Systems Pharmacology Database and Analysis Platform (TCMSP, https://old.tcmsp-e.com/tcmsp.php). Oral bioavailability (OB)≥30% and drug similarity (DL)≥0.18 were used as screening conditions to obtain the main active metabolites and their action targets. The molecular structure of active metabolites was obtained through literature search and Pubchem platform, and the pharmacokinetic absorption, distribution, metabolism and excretion (ADME) characteristics of Swiss ADME (http://www.swiss adme. ch/) (Daina et al., 2017) were used to screen active metabolites. Potential targets of active metabolites were predicted by Swiss Target Prediction (http://www.swisstargetprediction.ch/) (Daina et al., 2019). The name of the targets are normalized by using UnitProt database (UniProt Consortium, 2021).

#### 2.2.2 MAFLD related target retrieval

The ‘metabolic associated fatty liver disease’ was used as the key word to search and screen in the OMIM (https://omim.org/), Dis Genet (https://www.disgenet.org/), TTD (https://db.idrblab.net/ttd/) and Drug Bank (https://www.drugbank.com/), and remove duplicate targets to obtain related disease targets. The active components screened in 2.2.1 were crossed with MAFLD-related targets to obtain the main targets of *Z. bungeanum* Maxim. In the treatment of MAFLD, and the Venn diagram was drawn (Wang et al., 2024).

#### 2.2.3 Construct protein-protein interaction network (PPI) and screen key target

The potential gene targets of *Z. bungeanum* Maxim. For treating MAFLD were obtained by intersecting the predicted target of *Z. bungeanum* Maxim. Active metabolites and the targets of MAFLD. The obtained potential gene targets were added to the STRING database, the species were set as “*Homo sapiens*”, and the PPI network map was established. Import the result into Cytoscape 3.8.2 for visualization processing, use Analyze Network to analyze network topological features, and select core targets according to Degree value (Luo et al., 2024).

#### 2.2.4 GO functional enrichment and KEGG pathway enrichment analysis

GO gene enrichment analysis and KEGG pathway enrichment analysis were performed using Metascape platform (https://metascape.org/gp/index.html). The relevant data of molecular function (MF), biological process (BP), cell composition (CC) and KEGG pathway were established, and the results were visualized using Bioinformatics online tool (http://www.bioinformatics.com.cn) (Chen et al., 2024).

#### 2.2.5 Construction of active ingredient-MAFLD target-pathway network diagram of *Zanthoxylum bungeanum* maxim

CytoScape3.8.2 was used to construct the active ingredient-MAFLD target-pathway network diagram of *Z. bungeanum* Maxim. CytoScape3.8.2 built-in tools were used to analyze the topological characteristics of the network, and the core targets and important active components that exert drug efficacy were analyzed according to the network topology parameters (Liu et al., 2024).

#### 2.2.6 Molecular docking

The molecular docking between the active metabolites and the screened core targets was performed to verify the accuracy of the screened active metabolites of *Z. bungeanum* Maxim. On the potential core targets of MAFLD. The 2D structure of the active components of *Z. bungeanum* Maxim. Was searched by Pubchem website, and then the 3D structure of the active components was optimized by Chem3D software and saved as Mol2 format. Receptor proteins were downloaded from the RSCB PBD database (http://www.rcsb.org/pdb/home/home.do). PyMOL software was used to dehydrate and remove residues, and Autodock 1.5.6 software was used to hydrogenate the protein. The receptor protein and ligand small molecules were transformed into Pdbqt format, and the active components and core targets were molecularly docked using AutoDock Vina 1.1.2 software. According to the binding energy of the receptor and the ligand, the affinity was judged. The smaller the binding energy, the lower the affinity, and then the PyMOL software was used to visualize the active metabolites and target genes with higher molecular docking scores (Deng et al., 2024).

### 2.3 *In vivo* study

#### 2.3.1 Animal model establishment and grouping

Healthy SPF ICR mice, 4 weeks old, male, weighing 20 ± 2 g, purchased from SPF (Beijing) Biotechnology Co., Ltd., production license number: SCXK (Beijing) 2019–0,010. Feeding in a standard laboratory environment (temperature 20°C–26°C, humidity 40%–70%), light and dark cycles of 12 h, given standard animal feed, free feeding and drinking water. Adaptive feeding for 3 days before the experiment. All animal experiments were ethically approved by the Ethics Committee for Laboratory Animal Welfare of Chengdu University of Traditional Chinese Medicine (Project Ethics No. 2022–76) on 5 May 2022.

After adaptive feeding, all mice were randomly divided into 6 groups, with 8 mice in each group. They are control group (Control), model group (Model), quercetin low dose group (35 mg/kg), quercetin medium dose group (70 mg/kg), quercetin high dose group (140 mg/kg), positive control group (Metformin). The control group was fed a normal diet, and the other groups were fed a high-fat diet. At the beginning of the experiment, the control group was intraperitoneally injected with normal saline, and the other groups were intraperitoneally injected with tetracycline (150 mg/kg) to establish the MAFLD model. After 5 consecutive days, the quercetin treatment group was given different doses of quercetin (35, 70 and 140 mg/kg), the positive control group was given 200 mg/kg metformin, and the control group and the model group were given an equal volume of 2% Tween-80 once a day for 28 days.

#### 2.3.2 Histopathological examination

The liver tissue was fixed in 4% paraformaldehyde, dehydrated by alcohol and transparentized by xylene. Paraffin embedded and sectioned (thickness 3 μm). The sections were stained with hematoxylin and eosin (H&E) and observed under a microscope after dehydration, and the pathological changes were described (Ali et al., 2024).

#### 2.3.3 Oil red o staining

Oil red O staining was used to observe the accumulation of lipid droplets in liver tissue and hepatocytes. The liver tissue was made into frozen sections, and the cells were made into smears. The cells were fixed with 4% paraformaldehyde and stained in oil red O staining solution. Observed and photographed under an optical microscope (Lu and Wang, 2024).

#### 2.3.4 Biochemical index analysis

The levels of alanine aminotransferase (ALT), aspartate aminotransferase (AST), low density lipoprotein cholesterol (LDL-C), high density lipoprotein cholesterol (HDL-C), total cholesterol (TC) and triglyceride (TG) in serum or cells were detected by microcoder (Zhang J. et al., 2024). ALT and AST are indicators of liver parenchymatous injury, and AST and ALT are usually measured at the same time, and the ratio of the two can reflect different degrees of liver injury. LDL-C, HDL-C, TC and TG are the basic items of clinical blood lipid detection. Affected by many factors, the elevation of LDL-C is the main risk factor for the occurrence and development of atherosclerosis (Jacobson et al., 2015), A large number of epidemiological data show that serum HDL-C level is negatively correlated with the risk of atherosclerotic cardiovascular disease (ASCVD) (Gotto and Brinton, 2004), TC refers to the sum of cholesterol contained in various lipoproteins in the blood. The increase of TG may have a direct effect on atherosclerosis, and it is often used in combination with LDL-C and HDL-C.

#### 2.3.5 Lipid metabolism analysis

30 mg of liver tissue was weighed, add 200 μL of water and 20 μL of internal lipid standard, vortex at MP, add 800 μL of MTBE, vortex to mix, add 240 μL of pre-cooled methanol, vortex to mix, ultrasonic for 20 min in a low-temperature bath, leave at room temperature for 30 min, centrifugation at 14,000 g for 15 min at 10°C, take the upper layer of the organic phase, nitrogen gas blowing, and add 200 μL of 90% isopropanol/acetonitrile solution to dissolve, vortex thoroughly, take 90 μL of complex solution, centrifugation at 14,000 g for 15 min at 10°C. For mass spectrometry analysis, add 200 μL of 90% isopropanol/acetonitrile solution, vortex thoroughly, take 90 μL of the compound solution, centrifuge at 14,000 g 10°C for 15 min, take the supernatant into the sample for analysis. The separation was performed on a UHPLC Nexera LC-30A ultra performance liquid chromatography system. The chromatographic column was CSH C18. The chromatographic conditions were as follows: mobile phase A (acetonitrile/water = 6:4, v/v) + 0.1% formic acid +0.1 mM ammonium formate, and mobile phase B (acetonitrile/isopropanol = 1:9, v/v)) + 0.1% formic acid +0.1 ammonium formate, at a flow rate of 300 μL/min, and the temperature of the column chamber was 45°C. The samples were analyzed by random injection sequence method to avoid the effect of signal fluctuation. The samples were separated by UHPLC and analyzed by mass spectrometry using a Q Exactive series mass spectrometer (Thermo Scientific). Electrospray ionization (ESI) was used to detect positive and negative ions. The ESI source conditions were as follows: heater temperature 300°C, sheath gas flow rate of 45 arb, auxiliary gas flow rate of 15 arb, scanning gas flow rate of 1 arb, spray voltage of 3.0 KV, capillary temperature of 350°C, S-lens RF level of 50%, MS1 scanning range: 200–1800. Mass-to-charge ratios of lipid molecules and lipid fragments were collected as follows: 10 fragmentation profiles were collected after each full scan (MS2 scan, HCD). 70,000 resolution at M/Z 200 for MS1 and 17,500 resolution at M/Z 200 for MS2 were used for the identification of peaks, peak extraction, and lipid identification (secondary identification) of the lipid molecules and internal standards using LipidSearch. LipidSearch was used to identify the peaks of lipid molecules and internal standards, extract the peaks, and identify the lipids (secondary identification). The main parameters were: precursor tolerance: 5 ppm, product tolerance: 5 ppm, product ion threshold: 5% (Yang et al., 2025).

### 2.4 *In vitro* experiment

#### 2.4.1 Cell model establishment and grouping

Human hepatoma HepG2 cells (Shanghai Cell Bank of the Chinese Academy of Sciences, Shanghai, China) were used to establish oleic acid-induced MAFLD model. HepG2 cells were cultured in ATCC-modified low-limit Eagle medium supplemented with 10% fetal bovine serum and 1% penicillin-streptomycin in a 5% carbon dioxide incubator at 37 C.

The concentration of oleic acid modeling and quercetin administration was determined by detecting the cytotoxicity of oleic acid and quercetin. Cell viability was detected by Cell Counting Kit-8 (CCK-8). Cells were seeded into 96-well culture plates at a density of 3 × 10^4^ cells/well, 100 μL per well. The concentration gradient of oleic acid was 0.05, 0.1, 0.15, 0.2, 0.25, 0.5, 0.75, 1.0 mM, and the treatment time was 24 h. Quercetin concentration gradient was 5, 10, 20, 30, 40, 80 μM, treated for 48 h. 10 μL CCK-8 was added to each well and incubated for 1 h. SpectraMax iD3 microplate reader was used to determine the absorbance at 450 nm.

The cells were divided into 5 groups: control group (Control), model group (Model, containing 0.5 mM oleic acid), low-dose quercetin group (5 μM, containing 0.5 mM oleic acid and 5 μM quercetin), medium-dose quercetin group (10 μM, containing 0.5 mM oleic acid and 10 μM quercetin), high-dose quercetin group (20 μM, containing 0.5 mM oleic acid and 20 μM quercetin).

#### 2.4.2 Hepatocyte transcriptome sequencing

Total RNA was isolated from HepG2 cells using TriZol. The quantity and purity of total RNA were controlled by NanoDrop ND-1000 (NanoDrop, Wilmington, DE, United States), and the integrity of RNA was detected by Bioanalyzer 2,100 (Agilent, CA, United States). RNA in cell samples was sequenced using the Illumina Novaseq 6,000 sequencing platform (LC Bio Technology CO., Ltd. Hangzhou, China). The obtained FASTQ format file was further processed into reads, and the reads of all samples were compared with the cell reference genome using HISAT2 (https://daehwankimlab.github.io/hisat2/). StringTie (https://ccb.jhu.edu/software/stringtie/) software was used to estimate the expression level of all transcripts, and the Fragments Per Kilobase Million (FPKM) value was calculated as the expression level of each gene, and the difference between groups was analyzed. DESeq2 software was used to analyze the differential expression of genes in two different groups, and the enrichment analysis of GO function and KEGG pathway was performed on the differentially expressed genes (Yang et al., 2024).

#### 2.4.3 qRT-PCR

Total RNA was isolated from HepG2 using TriZol, and *Gapdh* was used as an internal reference. The relative expression levels of genes in each sample and group were calculated using 2^−ΔΔCT^ (Kong et al., 2025). The qRT-PCR primer information is shown in Table 1.

**TABLE 1:** qRT - PCR primer sequence table.

Gene	Primer	Sequences
*Epha2*	Forward primer	TAA​GAG​GGC​AGA​CTG​TGA​A
	Reverse primer	CCAGGAAAGCAAGGGTTT
*Dusp1*	Forward primer	GCG​TCA​AGA​CAT​TTG​CTG​AA
	Reverse primer	GTC​GTC​GGG​AAT​AAT​ACT​GGT​A
*Csf1*	Forward primer	CCGTGACTTTCCCTTCCT
	Reverse primer	GTTCACTGCCCTTCCCTA
*Golga4*	Forward primer	ACCACCGTACTGAAGTTC
	Reverse primer	GTCACCCAATGTCACTCT
*Best1*	Forward primer	CTA​ACC​TAG​AAG​TCA​GCA​AGC
	Reverse primer	TTC​ATC​ATC​TGG​CAG​TGT​TC
*Tmsb4x*	Forward primer	AGA​CCA​GAC​TTC​GCT​CGT​A
	Reverse primer	CCT​GCT​TGC​TTC​TCC​TGT​T
*Gapdh*	Forward primer	GAA​GGT​GAA​GGT​CGG​AGT​C
	Reverse primer	GAA​GAT​GGT​GAT​GGG​ATT​TC

#### 2.4.4 Western blotting test

Total protein was extracted from liver tissue using RIPA lysis buffer at low temperature. The protein concentration was quantitatively determined by BCA protein detection kit. Equal amounts of protein samples were subjected to sodium dodecyl sulfate-polyacrylamide gel electrophoresis (SDS-PAGE) and transferred onto polyvinylidene fluoride (PVDF) membranes. After blocking in 5% skimmed milk powder for 2 h, the cells were incubated with primary antibody at 4 °C overnight. After washing three times with TBST, the cells were incubated with HRP conjugated Goat Anti-Rabbit IgG for 1 h. TBST was washed three times again and observed with ECL chemiluminescence detection kit. The results were quantified using ImageJ. GAPDH was used as an internal control (Deng et al., 2025).

### 2.5 Data processing and statistical analysis

The results were expressed as mean ± standard deviation. Data processing and statistical analysis were performed using IBM SPSS Statistics 25.0 (IBM Corp., Armonk, N.Y., United States). One-way analysis of variance (One-way ANOVA) was used when the homogeneity of variance was satisfied. The least significant difference (LSD) test was used for pairwise comparison between groups, and the non-parametric test (Kruskal Wallis test) was used otherwise. P < 0.05 was considered statistically significant. The histogram was drawn using GraphPad Prism 8.0 software (GraphPad, Boston, United States).

## 3 Results

### 3.1 Results of network analysis

#### 3.1.1 Acquisition of potent metabolites and potential targets and molecular docking validation results for the treatment of MAFLD by *Zanthoxylum bungeanum* maxim

**FIGURE 1:**
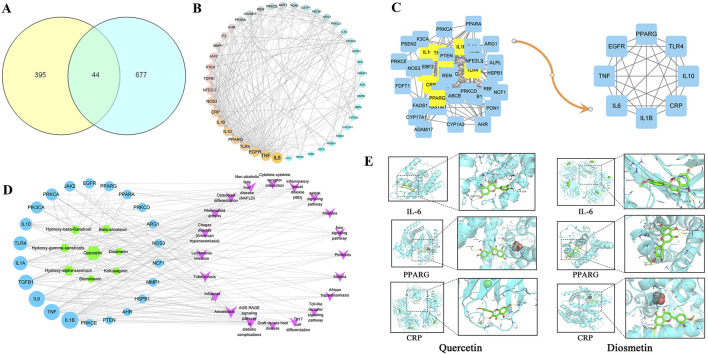
Target screening map. **(A)** Venn diagram of cross target of *Zanthoxylum bungeanum* Maxim. And MAFLD, **(B)** PPI network, **(C)** Core target screening map, **(D)** Active metabolites-MAFLD target-pathway network diagram, **(E)** Visualization of molecular docking.

A total of 8 active metabolite and 439 potential targets in *Z. bungeanum* Maxim. Were screened out by TCMSP database and target prediction. A total of 721 disease targets related to MAFLD were obtained from OMIM, Dis genet, TTD and Drug bank disease gene databases. There are 44 common targets between the active components of *Z. bungeanum* Maxim. And MAFLD (Figure 1A). The PPI network diagram was drawn in Cytoscape 3.8.2, and the topological characteristics were analyzed (Figure 1B). The nodes in the network graph represent proteins, and the degree value is represented by the number of edges connected to the same node. After optimizing the network, the larger the degree value, the larger the node area, the darker the color, and the thickness of the line represents the comprehensive score of the node in the PPI network. Eight core targets were identified, including interleukin-6 (IL-6), tumor necrosis factor (TNF), epidermal growth factor receptor (EGFR), peroxisome proliferator-activated receptor gamma (PPARG), Toll-like receptor 4 (TLR4), C-reactive protein (CRP), interleukin-1β (IL-1β) and interleukin-10 (IL-10) (Figure 1C).

The component-target-pathway network diagram was constructed using CytoScape3.8.2. (Figure 1D). The active components with larger degree values were quercetin and hydroxy-α-sanshool, and the targets with larger degree values were IL-1β, TNF, IL-6 and TGF-βI. It is indicated that these components and targets may be the key components and proteins of *Z. bungeanum* Maxim. In the treatment of MAFLD. Molecular docking was carried out between the active components and the core targets of screening, the docking results are shown in Table 2. The results showed that the binding energy of 91% components to their targets was ≤ −4.25 kcal/mol, especially the binding energy of quercetin and diosmetin to IL-6, PPARG and CRP was ≤ −7.0 kcal/mol, suggesting that these components had strong binding activity to the target. Finally, we used PyMOL software to visualize the molecular docking results of quercetin and diosmetin with IL-6, PPARG and CRP (Figure 1E). Therefore, based on the above predictions, we can make a preliminary assessment of the efficacy of the active constituents of *Z. bungeanum* Maxim. Against MAFLD.

**TABLE 2:** Binding energies of metabolites to core targets.

Metabolites	Score (Kcal/mol)							
	IL6	TNF	EGFR	PPARG	TLR4	CRP	IL1B	IL10
Kokusaginin	−6.1	−4.6	−4.7	−7.5	−4.3	−6.6	−5.5	−5.2
Skimmianin	−5.9	−4.3	−4	−6.3	−4.9	−6.8	−4.8	−5.3
Diosmetin	−7.1	−5.3	−5.3	−8.3	−5.6	−8.2	−6.9	−6.7
Beta-sitosterol	−6.6	−5	−5.1	−6.7	−6.4	−6.7	−7.2	−6.8
Quercetin	−7.8	−5.2	−5.3	−8.6	−5.2	−8.8	−6.7	−6.7
Hydorxy-gamma-sanshools	−4	−5	−2.5	−5.8	−4.1	−4.8	−3.8	−4.6
Hydroxy-beta-Sanshool	−5.5	−4.5	−4.1	−4.8	−4.6	−4.7	−3.7	−4.7
Hydroxy-alpha-sanshool	−4.5	−4.1	−3.5	−4.5	−4.1	−5.1	−4.8	−4.6

#### 3.1.2 GO functional enrichment and KEGG pathway enrichment analysis

**FIGURE 2:**
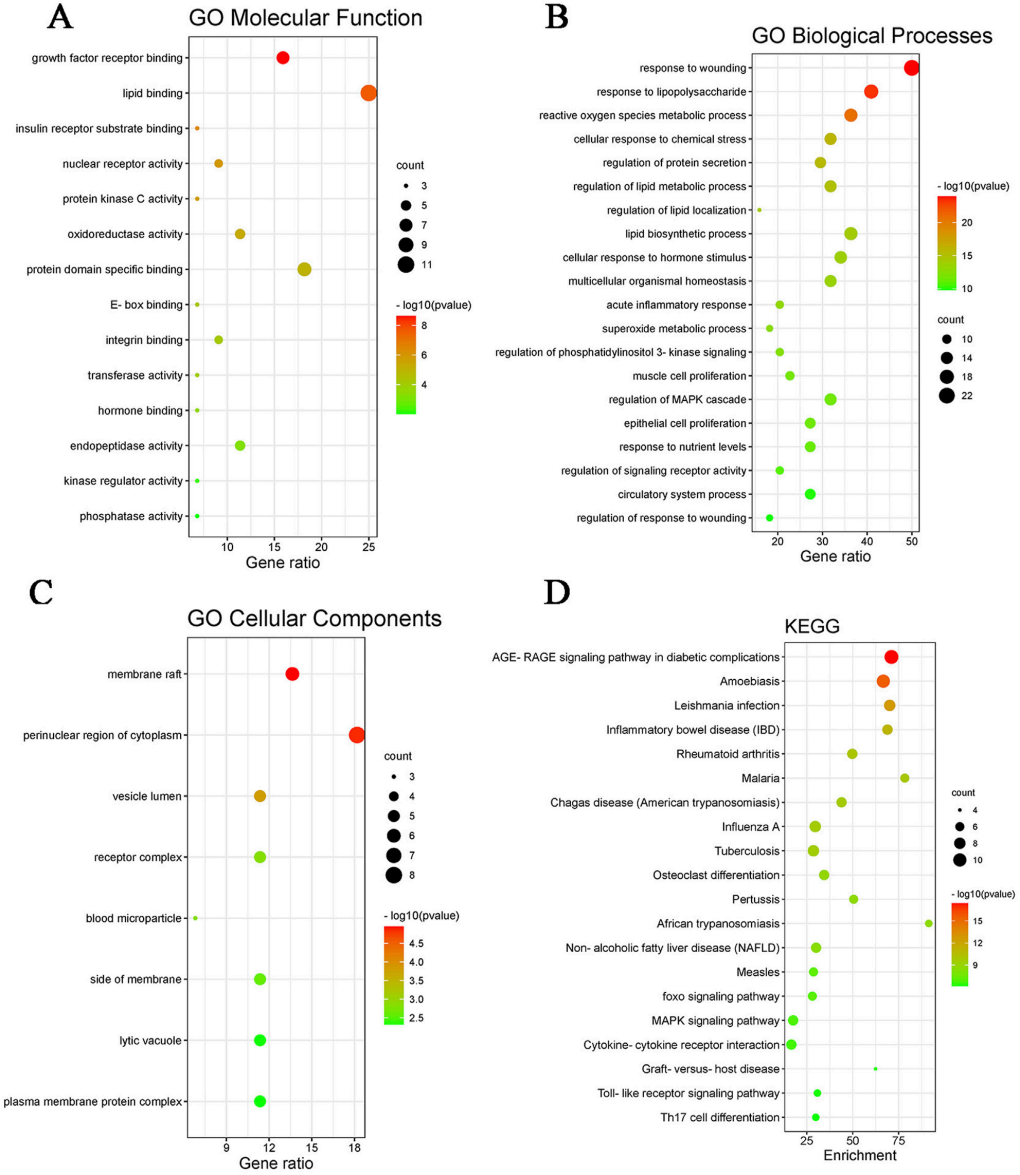
GO functional enrichment and KEGG pathway enrichment map. **(A)** GO molecular function map, **(B)** GO biological processes map, **(C)** GO cellular component map, **(D)** KEGG pathway enrichment map.

After preliminary evaluation, we hypothesized that quercetin may be a key active substance in the therapeutic effects of *Z. bungeanum* Maxim. In MAFLD, but its possible mechanism of action is not clear. Therefore, We used Meta scape platform to conduct enrichment analysis of MF(molecular function), BP(biological process) and CC(cellular component) of 44 potential action targets obtained. it was found that MF mainly involved lipid binding and insulin receptor substrate binding; BP mainly includes regulation of lipid metabolic process and lipid biosynthetic process. CC is mainly involved in membrane rafts and cytoplasmic perinuclear regions. We selected the top 20 pathways based on P-values to further investigate these findings and visualized them using the Bioinformatics online tool. The results of KEGG enrichment analysis mainly revealed AGE-RAGE signaling pathway, MAFLD, NAFLD, MAPK signaling pathway, *etc.* (Figure 2). These findings strongly suggest that quercetin, the main ingredient in *Z. bungeanum* Maxim., may exert its effect on MAFLD by participating in signaling pathways related to lipid metabolism. However, the mechanism needs to be further explored in *ex vivo* and *in vivo* experiments.

### 3.2 Results of *in vivo* experiments

#### 3.2.1 Effects of quercetin on liver histopathology and lipid accumulation in MAFLD mice

**FIGURE 3:**
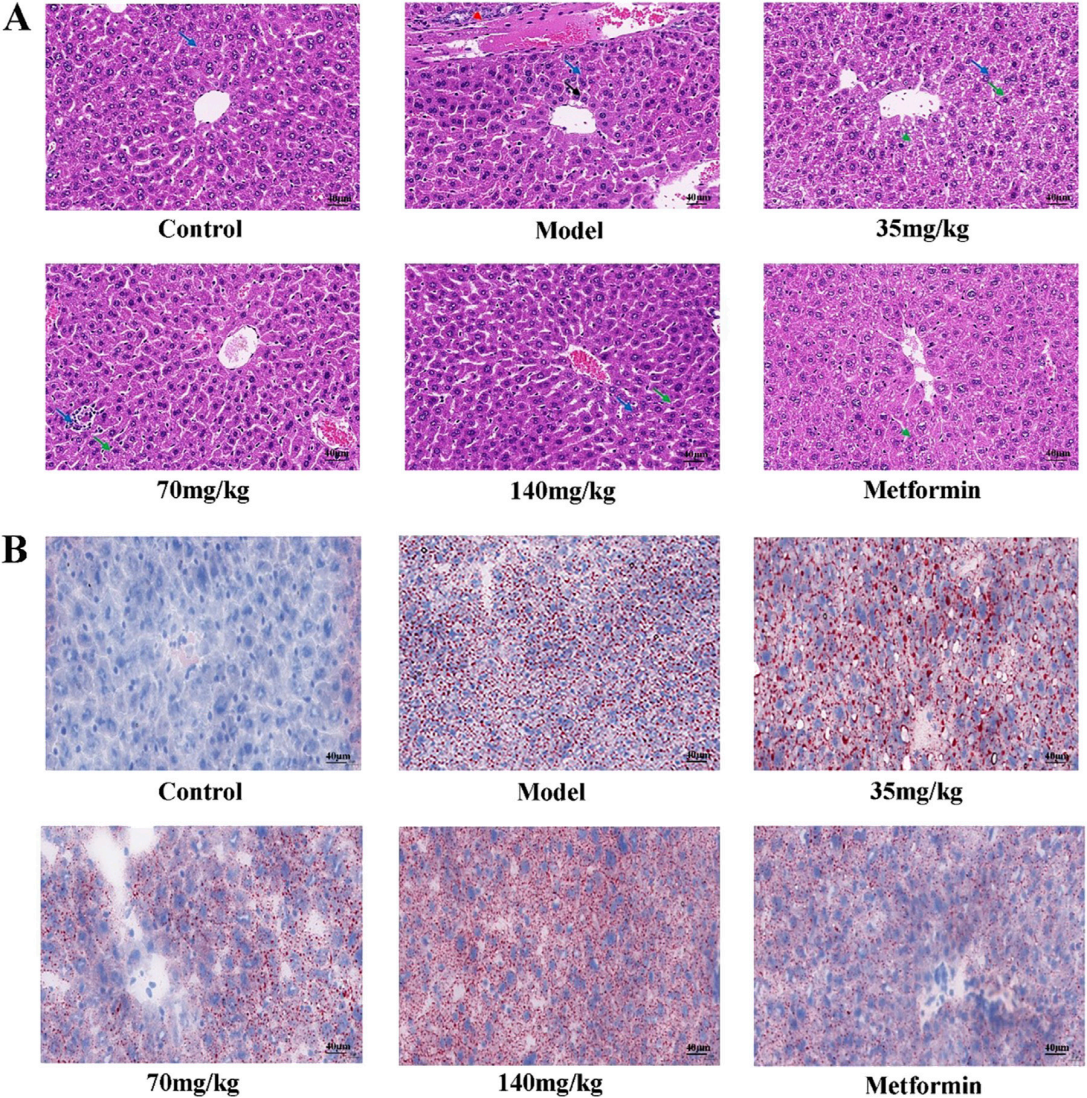
**(A)** Pathological morphology of mouse liver (×400 magnification) (n = 3). Solid blue arrows (hepatocyte necrosis), red dotted arrows (bile duct epithelial cell degeneration or necrosis), solid black arrows (hepatocyte atrophy), and solid green arrows (hepatocyte vesicular steatosis) **(B)** Mouse liver was stained with oil red O (×400 magnification) (n = 3).

The liver tissue morphology of mice in each group was observed by HE staining. It was found that the liver tissue morphology of mice in the control group was basically normal, and the liver of mice in the model group showed a large number of hepatocyte ballooning, hepatocyte atrophy, necrosis and interstitial inflammation. The pathological damage of the liver in the quercetin treatment group was improved to varying degrees, the number of necrotic cells was reduced, and the inflammatory response was alleviated. The number of hepatocyte necrosis in the liver of the positive group was significantly reduced, and the interstitial inflammatory response disappeared (Figure 3A). A large number of red lipid droplets were observed in the liver cells of the model group by oil red O staining, indicating that there was a large amount of lipid accumulation. The accumulation of lipid droplets in the quercetin treatment group and the positive group was improved (Figure 3B).

#### 3.2.2 Effects of quercetin on body weight and serum biochemical indexes in MAFLD mice

**FIGURE 4:**
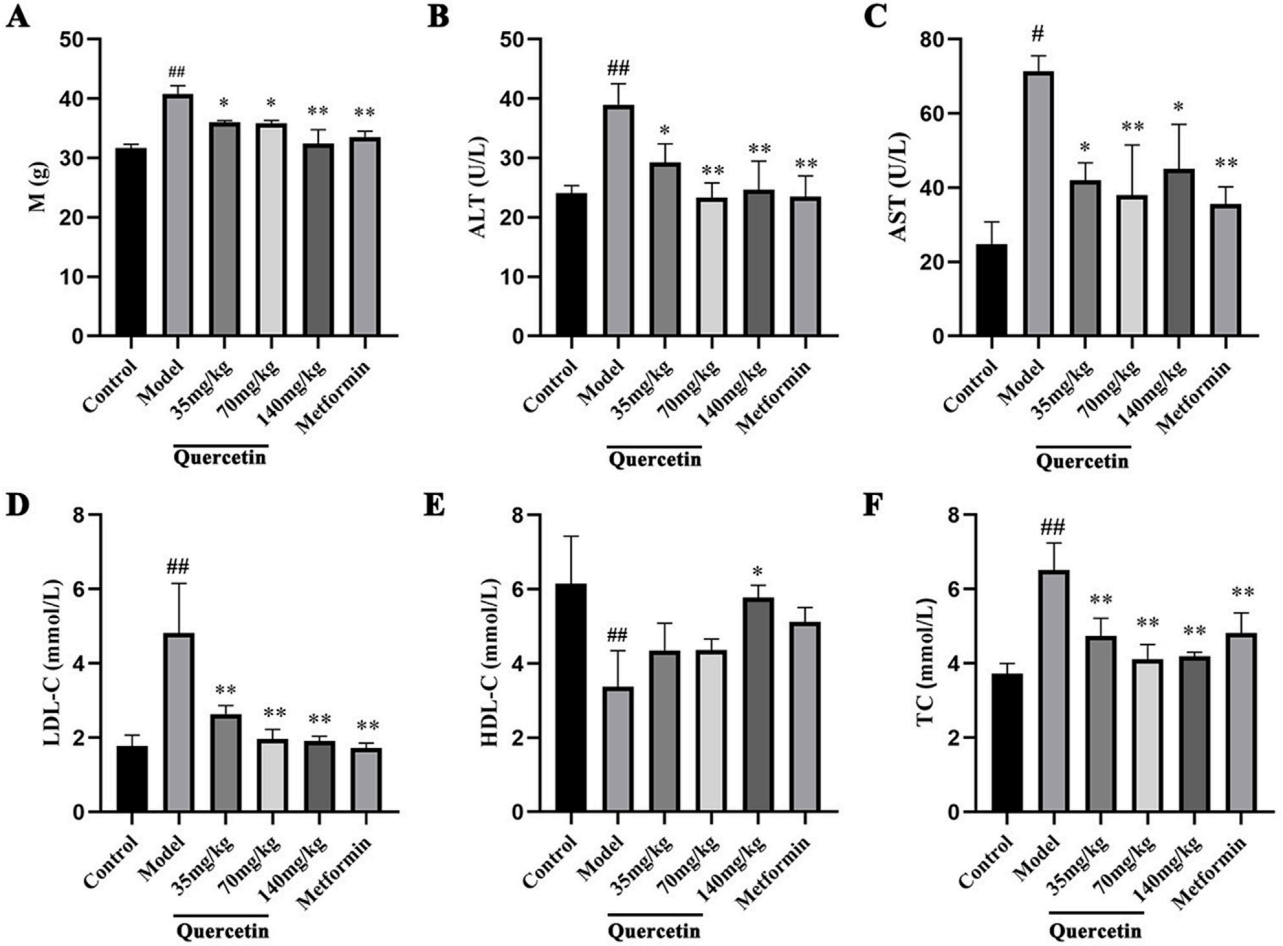
Effect of quercetin on MAFLD mice. **(A)** The body weight of ICR mice, **(B–F)** The serum levels of ALT, AST, LDL-C, HDL-C and TC in ICR mice. Values were represented the mean ± SD (n ≥ 3). ^#^P < 0.05, ^##^P < 0.01 vs. Control, ^*^P < 0.05, ^**^P < 0.01 vs. Model.

Compared with the control group, the body weight of the model group was significantly increased (p < 0.01). Compared with the model group, the body weight of mice in the quercetin treatment group and the positive group was significantly lower (p < 0.05), suggesting that quercetin may improve the weight gain of MAFLD mice (Figure 4A). Compared with the control group, the contents of ALT, AST, LDL-C and TC in the serum of MAFLD model mice were significantly increased, and the content of HDL-C was decreased (p < 0.05). After quercetin treatment, the contents of ALT, AST, LDL-C and TC in the serum were reversed to varying degrees. It is suggested that quercetin may improve steatosis and liver injury in MAFLD mice (Figures 4B–F).

#### 3.2.3 Effect of quercetin on lipid metabolism in MAFLD mice

**FIGURE 5:**
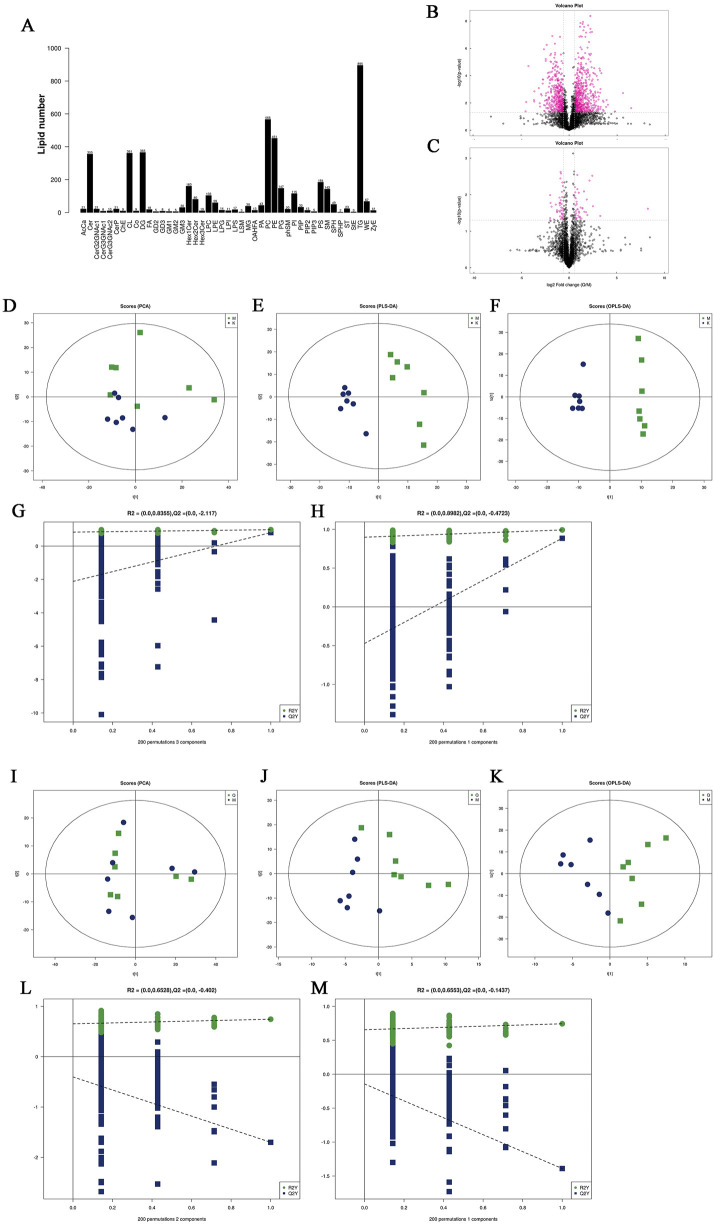
**(A)** Statistics of lipid subclasses and lipid molecules **(B, C)** Volcanic plot **(D)** PCA score plots of differential lipid molecules between model group and control group. **(E, F)** PLS-DA score plots and OPLS-DA score plots differential lipid molecules of differential lipid molecules between model group and control group. **(I)** PCA score plots of differential lipid molecules between quercetin group and model group. **(J, K)** PLS-DA score plots and OPLS-DA score plots differential lipid molecules of differential lipid molecules between quercetin group and model group **(G, H)**, **(L, M)** Cross-validation plot of the PLS-DA and OPLS-DA models with 200 times permutation tests. K: control group, M: model group, Q: quercetin group.

Lipid subclasses and lipid molecules in mouse liver were detected by lipidomics. A total of 45 lipids were identified, including 4,529 lipid molecules (Figure 5A). We used univariate statistical analysis to analyze the differences of these lipid molecules in the form of volcano maps (Figures 5B, C), in which the fold change (FC) > 1.5, p-value <0.05 was upregulated lipid molecules, FC < 0.67, p-value <0.05 was downregulated lipid molecules. Compared with the control group, there were 693 upregulated lipid molecules and 480 downregulated lipid molecules in the liver of MAFLD model mice. Compared with the model group, there were 41 upregulated lipid molecules and 44 downregulated lipid molecules in the liver of the quercetin treatment group. Principal component analysis (PCA), partial least squares discriminant analysis (PLS-DA) and orthogonal partial least squares discriminant analysis (OPLS-DA) were performed on the detected lipid molecules (Figure 5D–M). Among the PCA model parameters obtained by 7-fold cross-validation, the R^2^X between the model group and the control group was 0.506, and the R^2^X between the quercetin group and the model group was 0.597. PLS-DA and OPLS-DA models were tested by permutation test, and no over-fitting was observed.

Significantly different lipid molecules that meet OPLS-DA VIP >1 and p-Value <0.05 are considered as potential biomarkers. A total of 179 significantly different lipid metabolites were screened between the model group and the control group, including 18 categories of substances, including 55 diacylglycerols (DG), 37 phosphatidylethanolamines (PE), 18 phosphatidylcholines (PC), 13 ceramides (Cer), 11 cardiolipin (CL), 10 TG, 7 sphingomyelins (SM), 5 phosphatidylinositols (PI), 5 hexosylceramides (Hex1Cer), 4 Lys phosphatidylethanolamines (LPE), 4 cholesterol esters (ChE), 1 glycerol monoester (MG), two phosphatidylglycerols (PG), 1 pregnenolone ester (Co.), 1 fatty acid (FA), 1 phosphatidylserine (PS), 1 phosphatidylserine (PA) and 1 zymosterol (ZyE). We listed the differential lipid metabolites with OPLS-DA VIP >3 and p-Value <0.05, as shown in Table 3. A total of 29 significantly different lipid metabolites were screened between the quercetin administration group and the model group, including 7 types of substances, including 16 PE types, 4 C L types, 2 Lys phosphatidylcholine (LPC), 2 LPE, 2 DG, 2 Hex1Cer types, and 1 SM type. The specific differential lipid metabolites are shown in Table 4.

**TABLE 3:** Differential lipid metabolites in the liver between model group and control group.

No.	Lipidlon	Ion	Class	Molecular formula	CalMz	Rt (min)	VIP	Fold change	p-Value
1	ChE (18:1)	M + H	ChE	C_45_H_82_O_2_N_1_	668.634	16.671	15.139	8.115	0.048
2	DG (18:2/18:2)	M + H	DG	C_39_H_72_O_5_N_1_	634.541	9.029	13.993	0.413	0.008
3	DG (18:2/22:6)	M + H	DG	C_43_H_72_O_5_N_1_	682.541	8.352	13.002	0.422	0.005
4	PC (16:0/18:2)	M + H	PC	C_43_H_81_O_10_N_1_P_1_	802.560	7.687	8.275	0.652	0.010
5	DG (18:0/22:6)	M + H	DG	C_43_H_76_O_5_N_1_	686.572	10.044	7.728	9.335	0.000
6	PC (14:0/22:6)	M-H	PC	C_43_H_73_O_8_N_1_P_1_	762.508	7.546	7.018	0.596	0.018
7	DG (34:1e)	M + H	DG	C_37_H_72_O_4_Na_1_	603.532	16.507	6.576	2.116	0.011
8	DG (18:3/18:2)	M + H	DG	C_39_H_70_O_5_N_1_	632.525	8.202	6.251	0.238	0.004
9	SM (d42:2)	M + H	SM	C_48_H_94_O_8_N_2_P_1_	857.675	10.767	5.840	0.647	0.002
10	SM (d40:1)	M + H	SM	C_46_H_92_O_8_N_2_P_1_	831.660	10.851	5.371	1.539	0.014
11	Hex1Cer(d14:0/22:6)	M + H	Hex1Cer	C_43_H_72_O_10_N_1_	762.516	7.546	5.110	0.720	0.005
12	Cer(d18:1/24:1)	M + H	Cer	C_43_H_82_O_5_N_1_	692.620	12.129	5.081	0.801	0.046
13	PE (16:0/22:6)	M-H	PE	C_43_H_73_O_8_N_1_P_1_	762.508	7.510	5.050	0.740	0.002
14	DG (20:3/18:2)	M + H	DG	C_41_H_74_O_5_N_1_	660.556	9.601	4.860	1.641	0.041
15	ChE (2:0)	M + H	ChE	C_29_H_49_O_2_	429.373	6.009	4.746	9.450	0.002
16	DG (36:4e)	M + H	DG	C_39_H_71_O_4_	603.535	16.413	4.646	2.098	0.012
17	DG (34:1e)	M + H	DG	C_37_H_72_O_4_Na_1_	603.532	16.326	4.640	2.107	0.012
18	DG (18:0/18:2)	M + H	DG	C_39_H_76_O_5_N_1_	638.572	10.896	4.613	2.122	0.046
19	DG (22:3/18:2)	M + H	DG	C_43_H_78_O_5_N_1_	688.587	10.465	4.595	2.833	0.001
20	DG (34:3e)	M + H	DG	C_37_H_68_O_4_Na_1_	599.501	14.125	4.588	1.213	0.004
21	DG (36:6e)	M + H	DG	C_39_H_67_O_4_	599.503	14.114	4.560	1.212	0.003
22	Cer (d18:1/18:0)	M + H	Cer	C_37_H_72_O_5_N_1_	610.542	9.935	4.108	2.444	0.000
23	Cer (m17:1/19:0 + O)	M + H	Cer	C_37_H_72_O_5_N_1_	610.542	9.940	4.063	2.444	0.000
24	TG (18:1/18:1/18:1)	M + H	TG	C_57_H_108_O_6_N_1_	902.817	16.442	4.032	3.139	0.012
25	TG (16:0/18:1/18:1)	M + H	TG	C_55_H_106_O_6_N_1_	876.801	16.398	3.941	2.057	0.038
26	DG (38:6e)	M + H	DG	C_41_H_71_O_4_	627.535	7.465	3.938	1.503	0.005
27	DG (12:1e/24:2)	M + H	DG	C_39_H_72_O_4_Na_1_	627.532	7.459	3.938	1.503	0.005
28	CL (82:11)	M-H	CL	C_91_H_154_O_17_P_2_	790.534	8.576	3.753	0.787	0.010
29	PE (16:0/19:0)	M + H	PE	C_40_H_81_O_8_N_1_P_1_	734.569	8.535	3.753	0.789	0.021
30	TG (18:1/18:1/18:2)	M + H	TG	C_57_H_106_O_6_N_1_	900.801	16.250	3.724	2.525	0.043
31	PE (18:1/18:2)	M + H	PE	C_41_H_76_O_8_N_1_P_1_Na_1_	764.520	7.529	3.691	0.874	0.022
32	Cer (m40:1 + O)	M + H	Cer	C_41_H_80_O_5_N_1_	666.604	12.212	3.679	1.606	0.016
33	TG (18:0/18:1/18:1)	M + H	TG	C_57_H_110_O_6_N_1_	904.833	16.805	3.591	4.902	0.006
34	PE (18:0/20:4)	M-H	PE	C_43_H_77_O_8_N_1_P_1_	766.539	8.985	3.566	1.431	0.000
35	Cer (d18:1/22:0)	M + H	Cer	C_41_H_80_O_5_N_1_	666.604	12.212	3.554	1.572	0.017
36	PE (18:1/20:3)	M + H	PE	C_43_H_78_O_8_N_1_P_1_Na_1_	790.536	7.607	3.533	0.855	0.016
37	Cer (d40:1)	M + H	Cer	C_41_H_80_O_5_N_1_	666.604	16.309	3.525	1.612	0.039
38	DG (34:4e)	M + H	DG	C_37_H_67_O_4_	575.503	6.527	3.508	0.535	0.000
39	DG (34:0e)	M + H	DG	C_37_H_74_O_4_Na_1_	605.548	16.746	3.332	2.636	0.004
40	PC (8:1e/10:1)	M + H	PC	C_26_H_51_O_7_N_1_P_1_	520.340	1.509	3.329	0.607	0.027
41	PE (18:1/20:2)	M + H	PE	C_43_H_80_O_8_N_1_P_1_Na_1_	792.551	8.656	3.306	0.827	0.019
42	Cer (m36:1 + O)	M + H	Cer	C_37_H_72_O_5_N_1_	610.542	10.342	3.304	2.143	0.002
43	PE (16:1/18:1)	M-H	PE	C_39_H_73_O_8_N_1_P_1_	714.508	8.052	3.246	0.756	0.006
44	Hex1Cer (d32:2)	M + H	Hex1Cer	C_39_H_72_O_10_N_1_	714.516	8.033	3.246	0.756	0.006
45	CL (78:10)	M-H	CL	C_87_H_148_O_1_7P_2_	763.510	7.504	3.245	0.766	0.001
46	PE (18:0/18:1)	M + H	PE	C_41_H_80_O_8_N_1_P_1_Na_1_	768.551	9.001	3.228	1.329	0.000
47	PE (16:1e/22:4)	M-H	PE	C_43_H_77_O_7_N_1_P_1_	750.544	9.388	3.144	2.718	0.000
48	DG (16:1/22:6)	M + H	DG	C_41_H_70_O_5_N_1_	656.525	7.927	3.143	0.349	0.003
49	DG (18:0/18:1)	M + H	DG	C_39_H_78_O_5_N_1_	640.587	11.925	3.040	3.352	0.000

**TABLE 4:** Differential lipid metabolites in the liver between quercetin group and model group.

No.	Lipidlon	Ion	Class	Molecular formula	CalMz	Rt (min)	VIP	Fold change	p-Value
1	PE (38:5e)	M-H	PE	C_43_H_77_O_7_N_1_P_1_	750.544	9.388	3.068	0.834	0.011
2	LPC (18:2)	M + H	LPC	C_27_H_51_O_9_N_1_P_1_	564.331	3.175	2.992	309.998	0.024
3	LPE (18:0)	M-H	LPE	C_23_H_47_O_7_N_1_P_1_	480.310	2.453	2.506	1.594	0.020
4	Hex1Cer (d33:0 + O)	M + H	Hex1Cer	C_39_H_78_O_9_N_1_	704.567	7.364	2.300	0.876	0.043
5	PE (18:0e)	M + H	PE	C_23_H_49_O_7_N_1_P_1_	482.324	2.452	2.190	1.338	0.005
6	CL (72:7)	M-H	CL	C_81_H_143_O_17_P_2_	1,449.981	14.667	2.139	0.825	0.045
7	PE (36:2e)	M + H	PE	C_41_H_80_O_7_N_1_P_1_Na_1_	752.556	9.514	1.971	0.847	0.011
8	PE (38:4p)	M + H	PE	C_43_H_79_O_7_N_1_P_1_	752.559	9.844	1.971	0.847	0.011
9	LPC (18:2)	M + H	LPC	C_26_H_51_O_7_N_1_P_1_	520.340	2.349	1.849	9.726	0.038
10	LPE (16:0)	M-H	LPE	C_21_H_43_O_7_N_1_P_1_	452.278	1.849	1.825	1.598	0.037
11	PE (16:0e)	M + H	PE	C_21_H_45_O_7_N_1_P_1_	454.293	1.854	1.771	1.379	0.005
12	PE (36:4p)	M + H	PE	C_41_H_75_O_7_N_1_P_1_	724.528	8.373	1.755	0.830	0.004
13	PE (37:1e)	M + H	PE	C_42_H_85_O_7_N_1_P_1_	746.606	9.305	1.724	0.792	0.028
14	DG (38:8e)	M + H	DG	C_41_H_66_O_4_Na_1_	645.485	13.396	1.720	0.763	0.040
15	PE (36:5e)	M-H	PE	C_41_H_73_O_7_N_1_P_1_	722.513	8.353	1.644	0.850	0.027
16	PE (38:1)	M + H	PE	C_43_H_84_O_8_N_1_P_1_Na_1_	796.583	9.749	1.515	0.818	0.016
17	PE (40:4)	M-H	PE	C_45_H_81_O_8_N_1_P_1_	794.571	9.733	1.483	0.817	0.010
18	CL (82:7)	M-H	CL	C_91_H_162_O_17_P_2_	794.565	9.727	1.483	0.817	0.010
19	PE (38:4p)	M + H	PE	C_43_H_79_O_7_N_1_P_1_	752.559	9.354	1.292	0.746	0.043
20	PE (37:1e)	M + H	PE	C_42_H_84_O_7_N_1_P_1_Na_1_	768.588	8.215	1.215	0.814	0.030
21	PE (39:4e)	M + H	PE	C_44_H_83_O_7_N_1_P_1_	768.590	8.205	1.202	0.819	0.044
22	PE (40:4p)	M + H	PE	C_45_H_83_O_7_N_1_P_1_	780.590	10.330	1.195	0.775	0.010
23	PE (40:5e)	M-H	PE	C_45_H_81_O_7_N_1_P_1_	778.576	10.281	1.173	0.800	0.019
24	CL (79:2)	M-H	CL	C_88_H_166_O_17_P_2_	778.581	10.269	1.173	0.800	0.019
25	CL (78:3)	M-H	CL	C_87_H_162_O_17_P_2_	770.565	10.171	1.161	0.607	0.012
26	PE (40:8)	M-H	PE	C_45_H_73_O_8_N_1_P_1_	786.508	6.642	1.104	0.821	0.031
27	Hex1Cer (d38:8)	M + H	Hex1Cer	C_45_H_72_O_10_N_1_	786.516	6.642	1.104	0.821	0.031
28	SM (d44:5)	M + H	SM	C_49_H_92_O_6_N_2_P_1_	835.669	11.370	1.081	5.429	0.003
29	DG (38:3e)	M + H	DG	C_41_H_76_O_4_Na_1_	655.564	9.751	1.006	0.738	0.012

**FIGURE 6:**
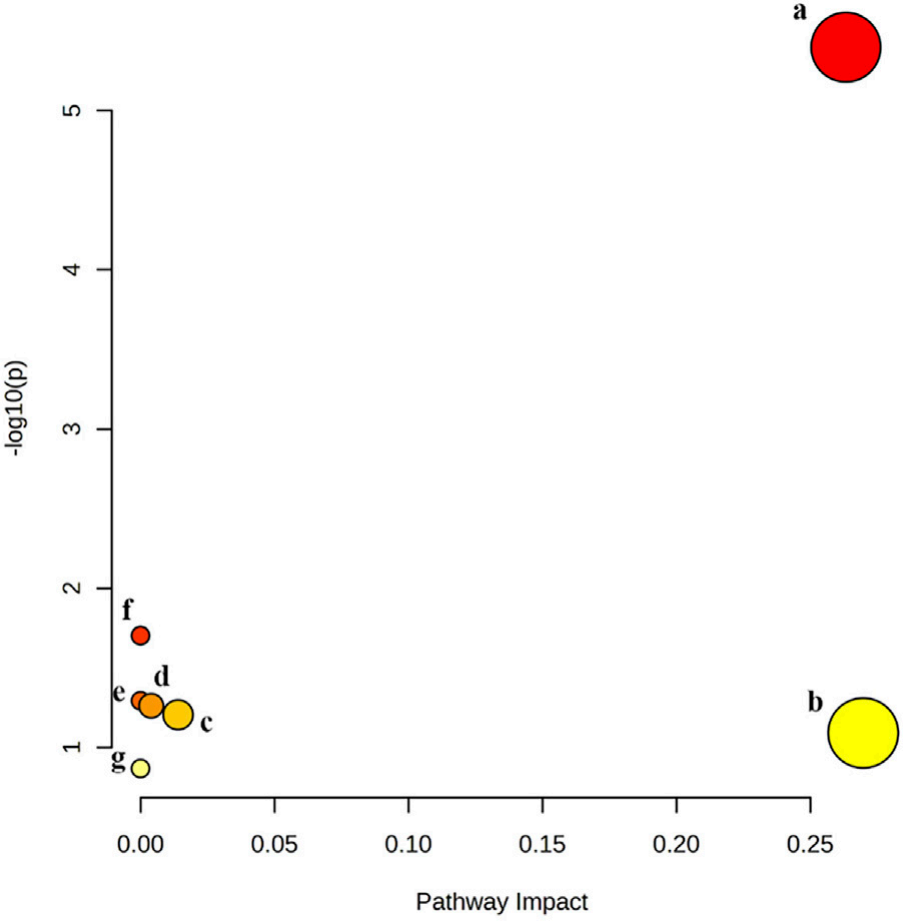
Six metabolic pathways related to changed biomarkers. **(a)** glycerophospholipid metabolism; **(b)** linoleic acid metabolism; **(c)** alpha-Linolenic acid metabolism; **(d)** glycosylphosphatidylinositol (GPI)-anchor biosynthesis; **(e)** glycerolipid metabolism; **(f)** sphingolipid metabolism; **(g)** arachidonic acid metabolism.

Pathway analysis of significant differential metabolites was performed by MetaboAnalysis 5.0 website (https://www.metaboanalyst.ca/). The results showed that quercetin treatment of MAFLD may be involved in seven metabolic pathways, namely, Glycerophospholipid metabolism, Linoleic acid metabolism, alpha-Linolenic acid metabolism, Glycosylphosphatidylinositol (GPI)-anchor biosynthesis, Glycerolipid metabolism, Sphingolipid metabolism, Arachidonic acid metabolism (Figure 6). Among them, Glycerophospholipid metabolism is the most likely metabolic pathway involved in quercetin treatment of MAFLD.

### 3.3 Results of *in vitro* experiment

#### 3.3.1 Oleic acid-induced lipid accumulation in HepG2 cells

**FIGURE 7:**
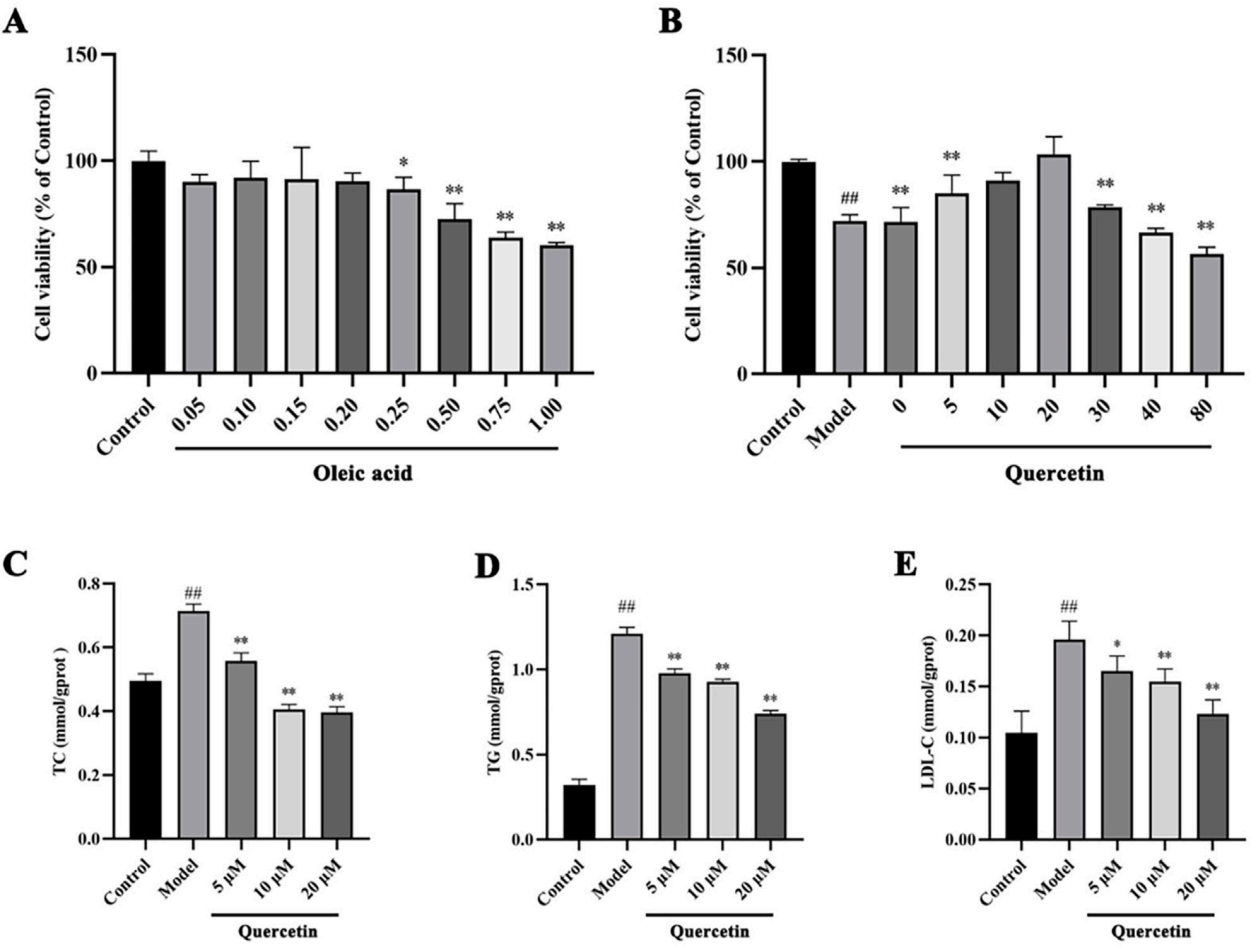
The effects of oleic acid and quercetin on HepG2 cells **(A)** Effect of oleic acid on the viability of HepG2 cells **(B)** Effect of quercetin on the viability of HepG2 cells induced by oleic acid **(C–E)** Effect of quercetin on the level of lipid markers in HepG2 cells induced by oleic acid. Values were represented the mean ± SD (n ≥ 3). ^#^P < 0.05, ^##^P < 0.01 vs. Control, ^*^P < 0.05, ^**^P < 0.01 vs. Model.

**FIGURE 8:**
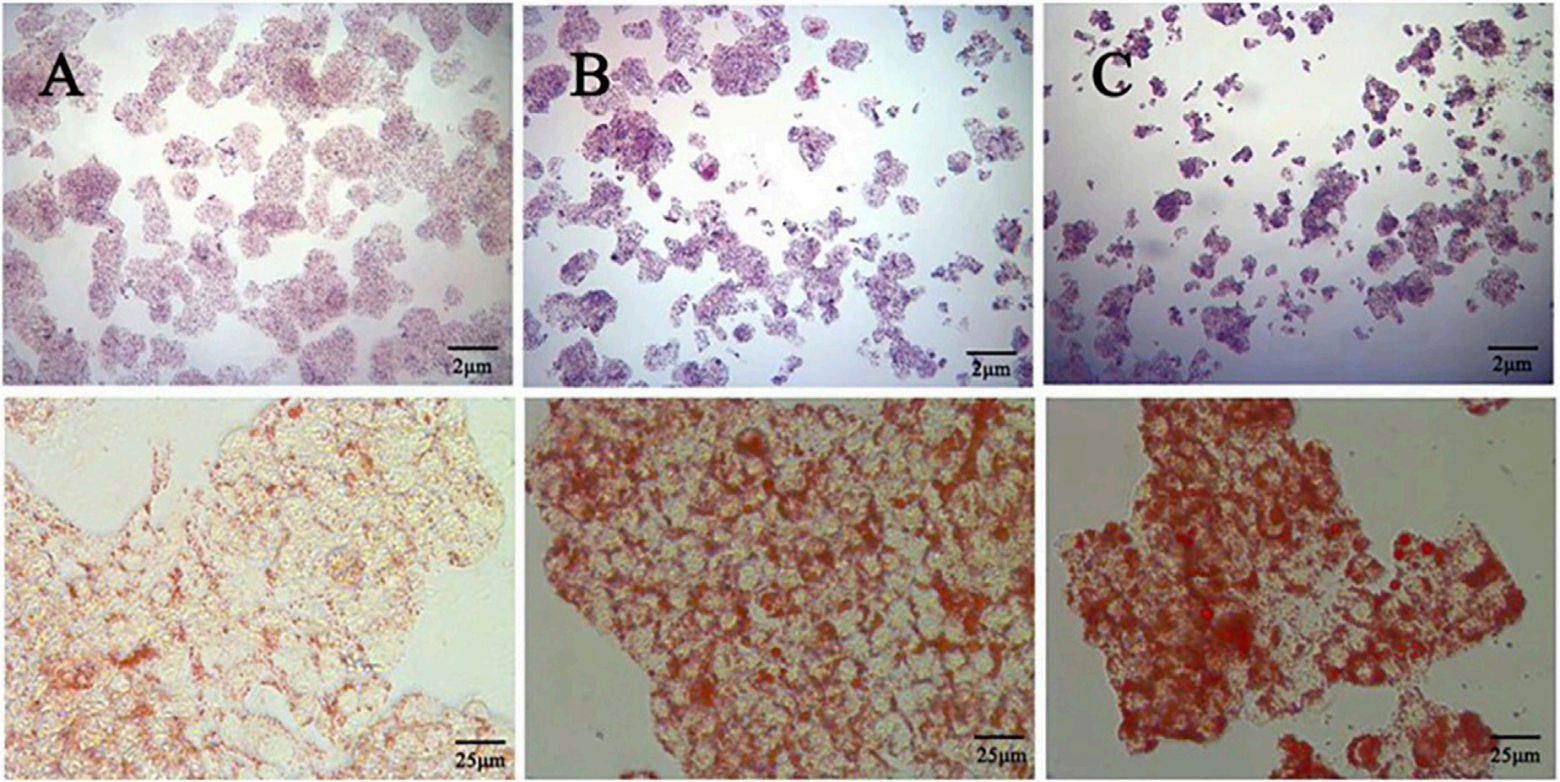
Comparison of oil red O staining between the control group and the OA induced group (400x) **(A)** Control group **(B)** 0.5 mM oleic acid group **(C)** 0.75 mM oleic acid group.

The effect of different concentrations of oleic acid on the viability of HepG2 cells was detected by CCK 8. Compared with the control group, oleic acid had no effect on cell viability when it was lower than 0.2 mM (p > 0.05), and had a significant effect on cell viability when it was higher than 0.25 mM (p < 0.05) (Figure 7A). Oil red O staining was used to detect the lipid accumulation of HepG2 cells induced by oleic acid. Compared with the control group, the accumulation of lipid droplets was obvious after induction with 0.5 mM and 0.75 mM oleic acid, suggesting a large amount of lipid accumulation. However, 0.75 mM oleic acid induced significant changes in cell morphology. Therefore, we chose 0.5 mM oleic acid to establish the MAFLD model of HepG2 cells (Figure 8).

#### 3.3.2 Quercetin improved the number of lipid droplets in HepG2 cells induced by oleic acid

**FIGURE 9:**
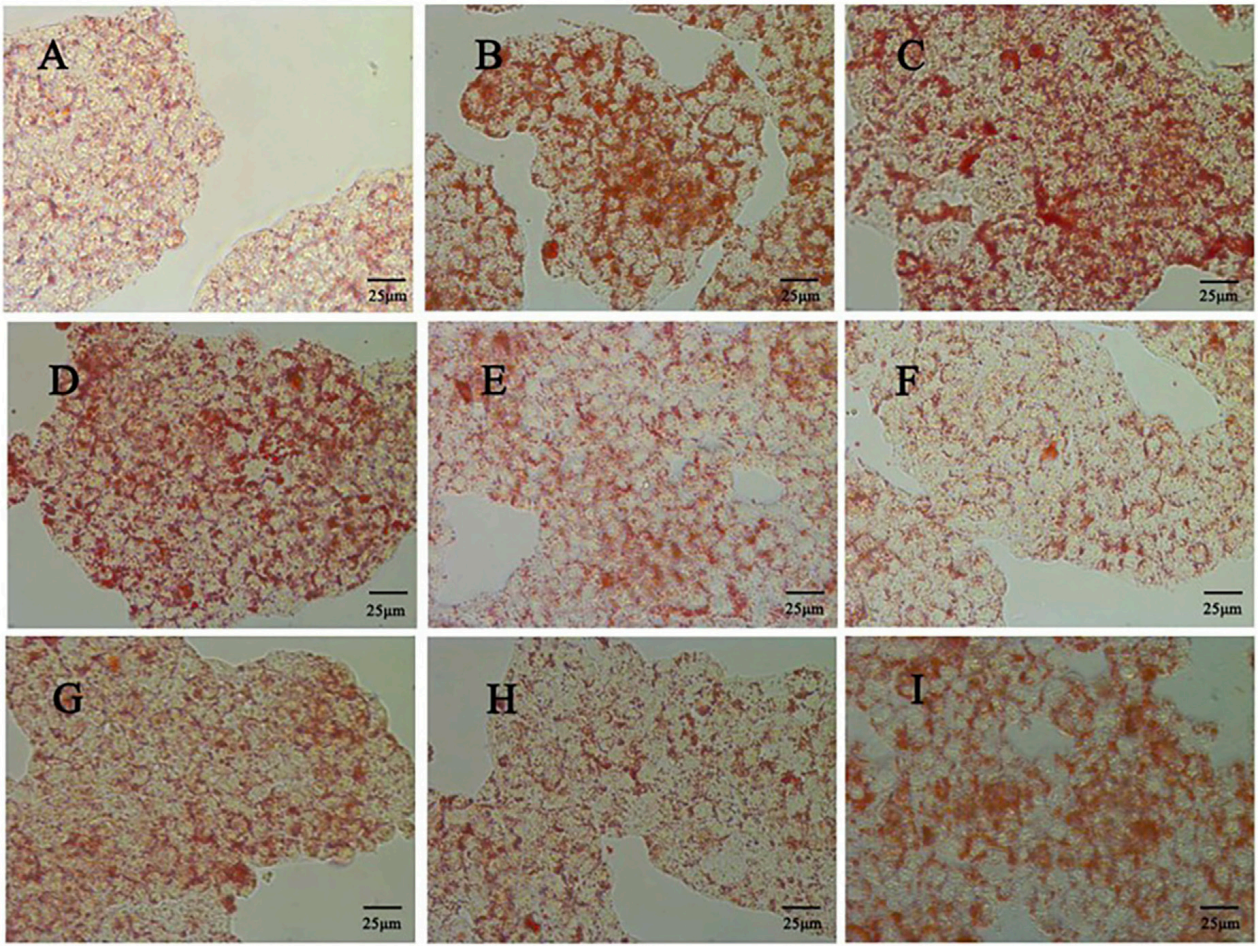
Effect of different doses of quercetin on lipid droplet accumulation in HepG2 cells (400x) **(A)** Control group **(B)** Model group; **(C)** 0 μΜ quercetin group **(D)** 5 μΜ quercetin group; **(E)** 10 μΜ quercetin group **(F)** 20 μΜ quercetin group; **(G)** 30 μΜ quercetin group **(H)** 40 μΜ quercetin group **(I)** 80 μΜ quercetin group.

Firstly, CCK 8 was used to detect the effect of quercetin on the viability of HepG2 cells. Compared with the control group, the cell viability of the model group was significantly decreased (p < 0.01). Compared with the model group, 5–20 μM quercetin significantly increased cell viability in a dose-dependent manner (Figure 7B). Secondly, oil red O staining showed that compared with the model group, 10 μM and 20 μM quercetin could significantly reduce the area of lipid droplets induced by oleic acid, and the reduction effect of 20 μM dose was more obvious. Quercetin above 30 μM could reduce the number of lipid droplets, but the morphology of lipid droplets increased, indicating that higher doses of quercetin may affect cells (Figure 9). In Figures 7C–E, compared with the control group, the TC, TG and LDL-C of the model group were significantly increased (p < 0.01); compared with the model group, quercetin treatment significantly reduced the levels of these three lipid markers (p < 0.05).

#### 3.3.3 Quercetin improves transcriptomic sequencing of MAFLD cell model

**FIGURE 10:**
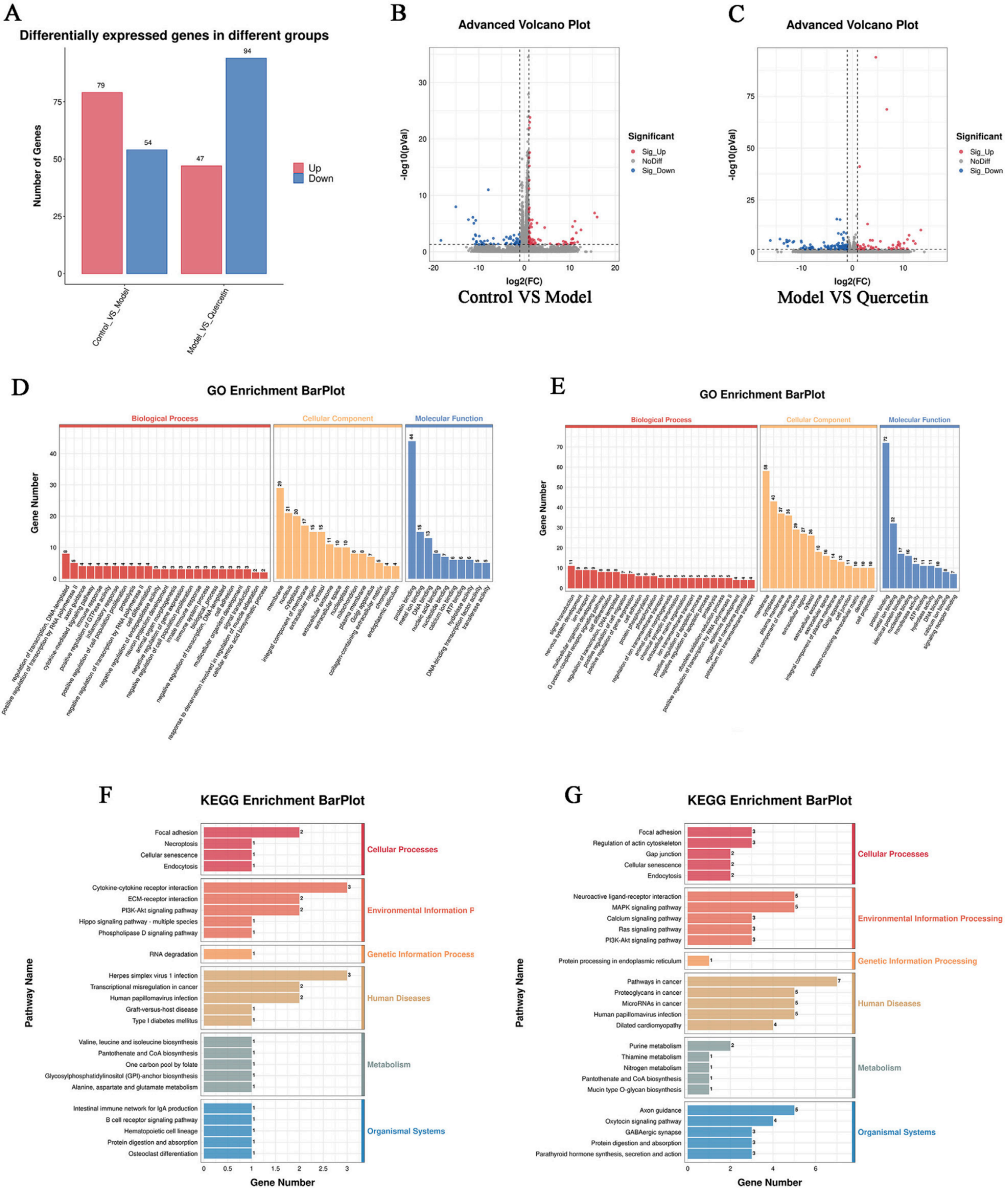
**(A)** Statistical map of up and down frequency modulation of differentially expressed genes, **(B)** Differential expression volcano map of control group and model group **(C)** Differential expression volcano map of model group and quercetin group. In the differential expression volcano map, the upregulated expression genes were represented by red dots, and the downregulated expression genes were represented by blue dots, **(D, F)** GO and KEGG enrichment analysis of differential genes between model group and control group **(E, G)** GO and KEGG enrichment analysis of differential genes between quercetin group and model group.

In order to explore the potential molecular mechanism of quercetin in improving MAFLD in cell models, we performed RNA sequencing on cells in the control group, model group, and 20 μM quercetin group. The sequencing quality factor Q30 of all samples reached more than 97.3%, indicating that the quality of sequencing data was good. Quantitative analysis of differential gene expression based on FPKM, as shown in Figure 10A. Compared with the control group, there were 133 differentially expressed genes (DEGs) in the model group, of which 79 genes were upregulated and 54 genes were downregulated. Compared with the model group, there were 141 differential genes in the quercetin group, of which 47 genes were upregulated and 94 genes were downregulated (Figures 10B, C).

GO enrichment analysis of differential genes showed that the main functions of differential genes in the model group and the control group were transcriptional regulation, positive regulation of transcription by RNA polymerase II, cytokine-mediated signal transduction pathway and immune response during biological processes. The differentially expressed genes mainly enriched in cell components include cell membrane and its organic composition, nucleus, cytoplasm, etc. In the molecular functional classification, including protein binding, metal ion binding, DNA binding, nucleic acid and nucleotide binding (Figures 10D, E). The main enriched functions of differential genes in quercetin group and model group were signal transduction, nervous system development, ion transport, multicellular organism development and immune response in biological processes. The differentially expressed genes mainly enriched in cell components included cell membrane and its organic composition, cytoplasm and cytoplasmic membrane, nucleus, etc*.* In the molecular functional classification, including protein binding, metal ion binding (Figures 10F, G).

#### 3.3.4 Quercetin decreased the expression levels of *Epha2, Dusp1, Csf1, Golga4, Best1* and *Tmsb4x* genes in HepG2 cells induced by oleic acid

The relative expression levels of *Epha2, Dusp1, Csf1, Golga4, Best1* and *Tmsb4x* genes were consistent with the change trend of the corresponding abundance value (FPKM) in RNA-Seq analysis. Compared with the control group, the expression levels of *Epha2, Dusp1, Csf1, Golga4, Best1* and *Tmsb4x* genes in the model group were significantly upregulated (p < 0.01). After quercetin treatment, the expression levels of *Epha2, Dusp1, Csf1, Golga4, Best1* and *Tmsb4x* genes were significantly downregulated (p < 0.01) (Table 5).

**TABLE 5:** Validation of qRT-PCR for candidate differentially expressed genes **(** $\bar{x}\pm s$ , n ≥ 3).

Group	Dose (μM)	*Epha2*	*Dusp1*	*Csf1*	*Golga4*	*Best1*	*Tmsb4x*
Control	-	0.28 ± 0.05	0.25 ± 0.05	0.41 ± 0.05	0.45 ± 0.04	0.45 ± 0.04	0.27 ± 0.05
Model	-	1.07 ± 0.01^##^	1.20 ± 0.09^##^	0.74 ± 0.29	1.28 ± 0.24^#^	1.28 ± 0.24^#^	0.59 ± 0.16
Quercetin	20	0.46 ± 0.01**	0.36 ± 0.11**	0.45 ± 0.00	0.26 ± 0.05**	0.26 ± 0.05**	0.56 ± 0.25

Values were represented the mean ± SD (n = 3). ^#^P < 0.05. ^##^P < 0.01 vs. Control. *P < 0.05. **P < 0.01 vs. Model.

#### 3.3.5 Quercetin can improve oleic acid-induced MAFLD by up-regulating p-p38 and pERK1/2 and down-regulating GPX4, SCL3A2 and xCT

**FIGURE 11:**
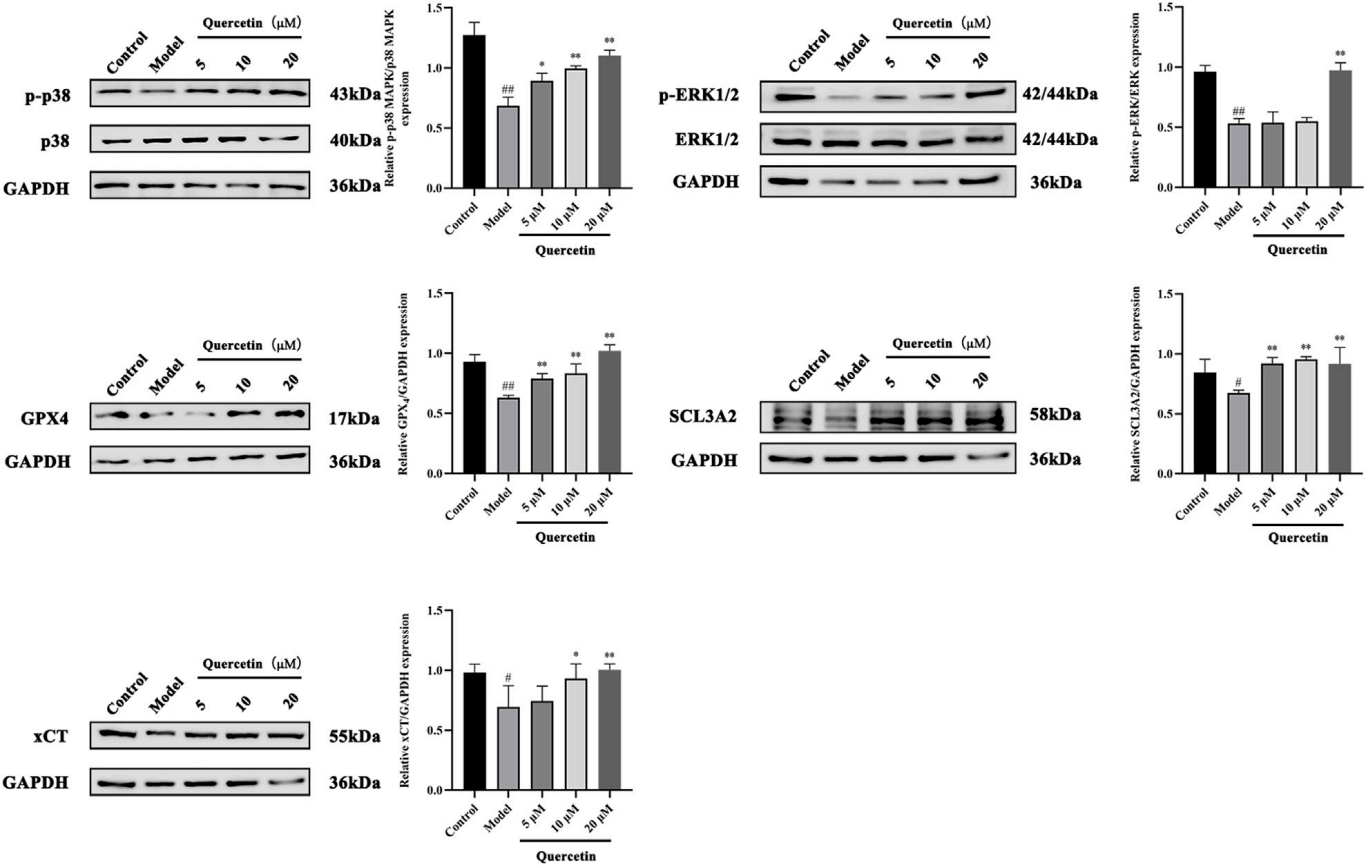
Effect of quercetin on expression of related proteins in mouse liver tissue. Values were represented the mean ± SD (n = 3). ^#^P < 0.05, ^##^P < 0.01 vs. Control, ^*^P < 0.05, ^**^P < 0.01 vs. Model.

MAPK signaling pathway plays an important role in the metabolism of MAFLD. Western blot analysis showed that compared with the control group, the expression of phosphorylated p38 MAPK and ERK1/2 protein in HepG2 cells treated with oleic acid was significantly decreased (p < 0.01), while quercetin treatment reversed this situation and upregulated the expression of phosphorylated p38 MAPK and ERK1/2 protein. Compared with the control group, the expression of GPX4, SCL3A2 and xCT protein in HepG2 cells induced by oleic acid was significantly decreased (p < 0.01), and the expression of GPX4, SCL3A2 and xCT protein was increased after quercetin treatment (p < 0.05) (Figure 11). It is suggested that quercetin may inhibit ferroptosis through the p38 MAPK/ERK signaling pathway, thereby alleviating the progression of MAFLD.

## 4 Discussion

MAFLD has become a leading cause of chronic liver disease worldwide. Disturbed lipid metabolism is the main predisposing factor for MAFLD, which has been reported to affect up to 70% of overweight people and more than 90% of morbidly obese people (Bourganou et al., 2025). However, there is still no satisfactory strategy to treat MAFLD induced by obesity. Ideal drugs for the treatment of MAFLD not only inhibit hepatic steatosis, but also ameliorate metabolic diseases associated with obesity.

Zanthoxylum plants have been shown to have a wide range of biological activities, including *Z. bungeanum* Maxim (Huang Y. et al., 2023).、*Zanthoxylum rhetsa* (Imphat et al., 2021; Rahman et al., 2002)*、Zanthoxylum khasianum*, etc. Santhanam et al. found that ethyl acetate extract of *Z. rhetsa* significantly inhibited the increase of pro-inflammatory cytokines in HDF cells induced by ultraviolet radiation b (Santhanam et al., 2018). Barman et al. ‘s study suggested that *Z. rhetsa* ethanol extract had significant free radical scavenging activity, and oral glucose tolerance test found that the extract could effectively reduce the blood glucose level of diabetic mice. Recent studies have shown that *Z. bungeanum* Maxim. Can attenuate high-fat diet (HFD)-induced MAFLD by improving fat accumulation (Peng et al., 2024).

In this study, we predicted the important active components that play a role in the treatment of MAFLD by *Z. bungeanum* Maxim. Including quercetin, geranylgeranyl, hydroxy-α-sanshool and hydroxy-β-sanshool based on network analysis, among which quercetin corresponded to the most potential targets, network analysis hypothesizes that quercetin may be the main active ingredient in the therapeutic effects of *Z. bungeanum* Maxim. On MAFLD. Quercetin is a secondary metabolite of plants with a variety of health benefits. Numerous studies have found quercetin to have some anti-inflammatory effects in MAFLD mice (Jiang et al., 2025; Lu et al., 2024; Markowska et al., 2024). The combination of quercetin and metformin has also been found to reduce cirrhosis by stimulating autophagy and reducing inflammatory cytokines via the cAMP/AMPK/SIRT1 signaling pathway (Afshari et al., 2023). A study by Cao P. et al. Found that quercetin prevents MAFLD via AMPK-mediated mitochondrial autophagy (Cao et al., 2023), and it has also been suggested that quercetin, by down-regulating the mTOR/YY1 signaling pathway converts cholesterol to bile acids, which leads to an increase in CYP7A1 activity, restores cholesterol homeostasis, and exerts hepatoprotective effects against T2DM-associated MAFLD (Yang et al., 2023).

In recent years, the “histology” approach has been increasingly applied to the study of drug mechanism of action, especially the combination of multi-omics techniques has provided new ideas for the elucidation of drug mechanism of action and drug discovery. In the present study, network analysis and molecular docking results predicted that quercetin may be the main active ingredient in *Z. bungeanum* Maxim. Exerting therapeutic effects in MAFLD, in addition, by establishing a mouse model and a cell model of MAFLD, and by detecting serum biochemical indexes, pathological HE staining and oil red O staining, it was found that quercetin could regress the MAFLD mice serum ALT, AST, LDL-C, HDL -C and TC levels, hepatic pathological changes were alleviated, and hepatic lipid accumulation was improved. Further analysis of hepatic lipid metabolism in MAFLD mice using lipidomics technology revealed that quercetin treatment of MAFLD may improve hepatic lipid metabolism disorders in mice through the Glycerophospholipid metabolic pathway. In addition, we evaluated the effect of quercetin on oleic acid-induced HepG2 cells by detecting TC, TG, LDL-C content and lipid accumulation in the cells. Differential genes and signaling pathways were screened by transcriptome sequencing and further validated by Western blot, and we obtained that quercetin may improve MAFLD through p38 MAPK/ERK signaling pathway.

The predicted results of network analysis in this study indicated that the core targets of *Z. bungeanum* Maxim. With therapeutic effects on MAFLD were IL-1β, TNF, IL-6 and TGF-βI. The molecular docking results further predicted that quercetin might be the main active substance in the therapeutic effects of Zanthoxylum bungeanum Maxim. On MAFLD. Quercetin is a flavonoid widely found in the plant kingdom, and studies have shown that quercetin has hepatoprotective effects against MAFLD, especially against hepatic steatosis and hepatitis (Chen et al., 2021b). A study found that quercetin reversed MAFLD symptoms by reducing oleic acid-induced secretion of inflammatory factors IL-8 and TNF-α in HepG2 cells. This is consistent with our network analysis predicting that the core targets of quercetin for MAFLD are IL-1β, TNF, IL-6 and TGF-βI, among others (Chen et al., 2021a). Subsequently, we used tetracycline combined with high-fat diet to establish a mouse MAFLD model and oleic acid to establish a HepG2 cell model. The liver is an important organ for lipid metabolism, and when hepatic fatty acid synthesis or uptake exceeds the liver’s capacity for oxidation or its output, lipid droplets accumulate in the liver parenchyma and triglyceride levels increase, causing MAFLD when lipids account for more than 5% of the liver’s wet weight (Gutiérrez-Cuevas et al., 2021). Tetracycline, an antibiotic that induces steatosis (Zhong et al., 2020), has been found to induce the transport of lipids to the liver, which in turn triggers the infiltration of hepatic fat and the formation of vesicular fatty liver disease. As fatty acids in the liver continue to rise, the level of oxidative stress also continues to rise, which results in a series of attacks on functional proteins, impeding the normal metabolism of fatty acids, and leading to the abnormal accumulation of triglycerides, which in turn exacerbates the formation of fatty liver disease. As fatty acid levels rise in the liver, oxidative stress levels also rise, causing a series of functional proteins to be attacked, preventing the normal metabolism of fatty acids and leading to an abnormal accumulation of triglycerides, which in turn exacerbates fatty liver formation (Steinberg et al., 2025). Therefore, we used tetracycline combined with a high-fat diet to model MAFLD. Similarly, *in vitro* models of steatosis are often used to explore the role of a drug in the treatment of fatty liver disease. Oleic acid is a monounsaturated fatty acid found in animals and plants, and the synthesis of palmitic acid by acyl coenzyme A is extended to stearic acid, which is desaturated to give oleic acid, which occupies about 40%–50% of the total free fatty acids, and is an important constituent of triglycerides stored in the cytoplasm. In some studies, 0.1 mM oleic acid was used to establish MAFLD model of HepG2 cells for *in vitro* experiments (Kaixuan et al., 2022). However, by combining the results of CCK-8 and oil red O staining, it was observed that the viability of HepG2 cells was significantly inhibited when the concentration of oleic acid was 0.5mM, and the accumulation of cell lipid drops was significantly increased, and the cell morphology did not change significantly. Therefore, in this study, the steatosis of HepG2 cells induced by 0.5 mM oleic acid was used to establish an *in vitro* model of MAFLD.

The p38 mitogen-activated protein kinase (p38 MAPK) is an important inflammatory factor and the basis of oxidative stress, and is involved in the regulation of nuclear factor E2-related factor 2 (Nrf2) and NF-κB in liver and metabolic disorders (Wang et al., 2023). We enriched the gene targets obtained by transcriptome sequencing to the MAPK signaling pathway may be involved in the treatment of MAFLD by quercetin, and further validated the related proteins on the MAPK signaling pathway by Western blot, and found that quercetin inhibits iron death through the p38 MAPK/ERK signaling pathway, thus alleviating the extension of MAFLD disease. It has been shown that quercetin significantly ameliorates hepatic dysfunction and host metabolic disorders in MAFLD mice (Shi et al., 2022) and inhibits MAFLD through AMPK-mediated mitochondrial phagocytosis (Cao et al., 2023). Quercetin also exhibits hepatoprotective activity in early-stage MAFLD rats by modulating fatty acids, inflammation, oxidative stress, and related metabolites (Xu et al., 2019). Flavonoids have been shown to improve hepatic steatosis by regulating glycerophospholipid metabolism in the treatment of MAFLD. Du Siyu et al. found that total flavonoids in Garcinia cambogia tea could reduce the *de novo* synthesis of fatty acids and regulate glycerophospholipid metabolism by targeting the PPAR signaling pathway, thereby improving hepatic steatosis (Du et al., 2024). Co-loaded liposomes prepared from Antarctic krill oil and quercetin have also been shown to be protective against oleic acid-induced lipoatrophy and oxidative stress in HepG2 cells (Li et al., 2024). In addition, quercetin-rich buckwheat tartare extracts can prevent alcoholic liver disease by regulating hepatic glycerophospholipid metabolism (Cao et al., 2022). Similarly, our study found that quercetin is metabolized *via* the Glycerophospholipid metabolic pathway regulates hepatic lipid metabolites in MAFLD mice, thereby ameliorating liver injury.

In the present study, quercetin was hypothesized to be the main active metabolite of *Z. bungeanum* Maxim. For the treatment of MAFLD, and its potential mechanism of action was further elucidated by *in vitro* experiments based on *in vivo* experiments. However, some limitations need to be recognized. First, the results of the network analysis predicted that quercetin might be the primary active substance for the pharmacological effects of *Z. bungeanum* Maxim., but quercetin itself belongs to the class of pan-assay interfering compound (PAINS) substances, which are substances with nonspecific, pharmacologically irrelevant *in vitro* and computer-simulated data. Therefore, it may non-specifically interfere with multiple assay systems in vitro experiments, and more rigorous control systems are needed to validate the specificity of its action (e.g., designing *in vitro* experiments using other non-PAINS active substances in peppercorns for multi-faceted validation). In addition, the mechanism of action of quercetin needs to be verified *in vivo* experiments because of such properties of quercetin. In this study, the mechanism of action of quercetin was only predicted *in vivo* by lipidomics techniques, but the mechanism was not further verified *in vivo* experiments. To address this issue, future studies should compare the efficacy of quercetin with other non-PAINS bioactive compounds derived from *Z. bungeanum* Maxim. And focus primarily on *in vivo* experimental data to further confirm its therapeutic specificity and to better interpret and validate its pharmacological findings. Second, although lipidomic analysis revealed seven key pathways for quercetin enrichment, we focused only on the glycerophospholipid metabolism pathway for subsequent validation. Other interesting pathways, such as linoleic acid metabolism and α-linolenic acid metabolism, deserve further exploration. Finally, in addition to lipidomics, the metabolomics dataset generated in this study still has untapped potential to provide insights into the mechanisms of MAFLD progression and interventions.

## 5 Conclusion

In conclusion, our study showed that quercetin, as a major active ingredient in *Z. bungeanum* Maxim., may regulate hepatic lipid metabolites via glycerophospholipid metabolism pathway in MAFLD mice, thereby ameliorating hepatic lipid accumulation and liver injury. Meanwhile, our results also indicated that quercetin was able to ameliorate MAFLD by reducing oleic acid-induced lipid accumulation in HepG2 cells and slow down the progression of MAFLD disease by inhibiting iron death through the p38 MAPK/ERK signaling pathway. Our study reveals the potential mechanism by which quercetin isolated from *Z. bungeanum* Maxim. Ameliorates MAFLD, and these results provide a theoretical basis for the mechanism of *Z. bungeanum* Maxim. Treatment for MAFLD.

## Data Availability

The transcriptomics data presented in this study have been submitted to the (NCBI) BioProject repository, with the accession number: PRJNA1241200. The original data of this article can be found in the article/Supplementary Material.

## Abbreviations

MAFLD metabolic-associated fatty liver disease; ALT aspartate aminotransferase; AST alanine aminotransferase; LDL-C low-density lipoprotein cholesterol; HDL-C high-density lipoprotein cholesterol; TC cholesterol; TG triglyceride; MF molecular function; BP biological process; CC cell composition; CCK-8 Cell Counting Kit-8; IL-6 interleukin-6; TNF tumor necrosis factor; p38 MAPK p38 mitogen-activated protein kinase.
