# Supplementary figures and images for: The mechanism study of quercetin isolated from Zanthoxylum bungeanum maxim. inhibiting ferroptosis and alleviating MAFLD through p38 MAPK/ERK signaling pathway based on lipidomics and transcriptomics
Source: Front Pharmacol. 2025 Mar 31;16:1517291. doi: 10.3389/fphar.2025.1517291 (PMC11994740; doi:10.3389/fphar.2025.1517291)

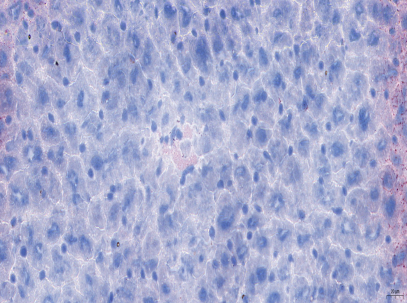

Supplement: Supplementary file 1 [file DataSheet1.zip › Raw data/Animal oil was stained with red O/Animal oil was stained with red O/Control.tif]

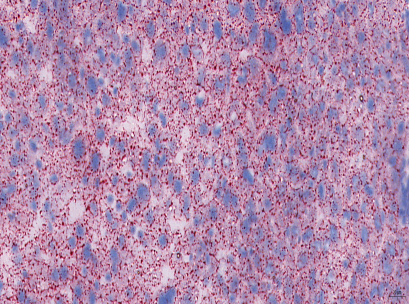

Supplement: Supplementary file 1 [file DataSheet1.zip › Raw data/Animal oil was stained with red O/Animal oil was stained with red O/High.tif]

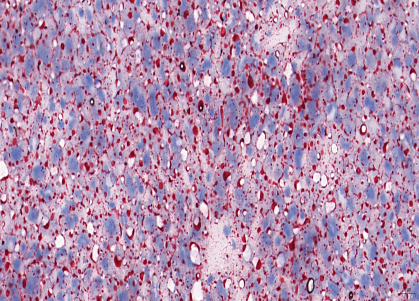

Supplement: Supplementary file 1 [file DataSheet1.zip › Raw data/Animal oil was stained with red O/Animal oil was stained with red O/LOW.tif]

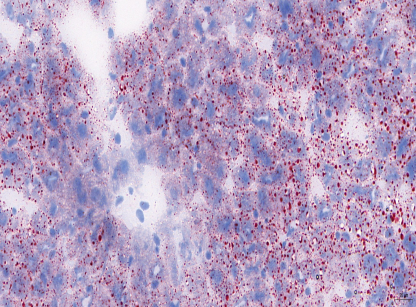

Supplement: Supplementary file 1 [file DataSheet1.zip › Raw data/Animal oil was stained with red O/Animal oil was stained with red O/Medium.tif]

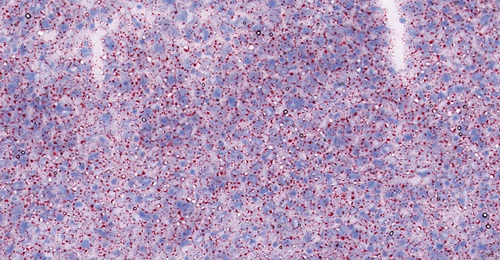

Supplement: Supplementary file 1 [file DataSheet1.zip › Raw data/Animal oil was stained with red O/Animal oil was stained with red O/Model.jpg]

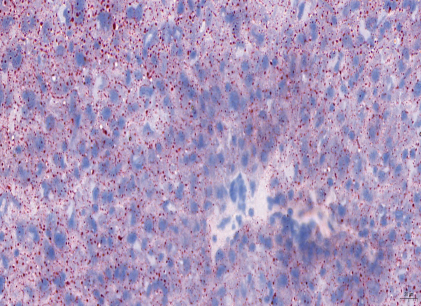

Supplement: Supplementary file 1 [file DataSheet1.zip › Raw data/Animal oil was stained with red O/Animal oil was stained with red O/positive.tif]

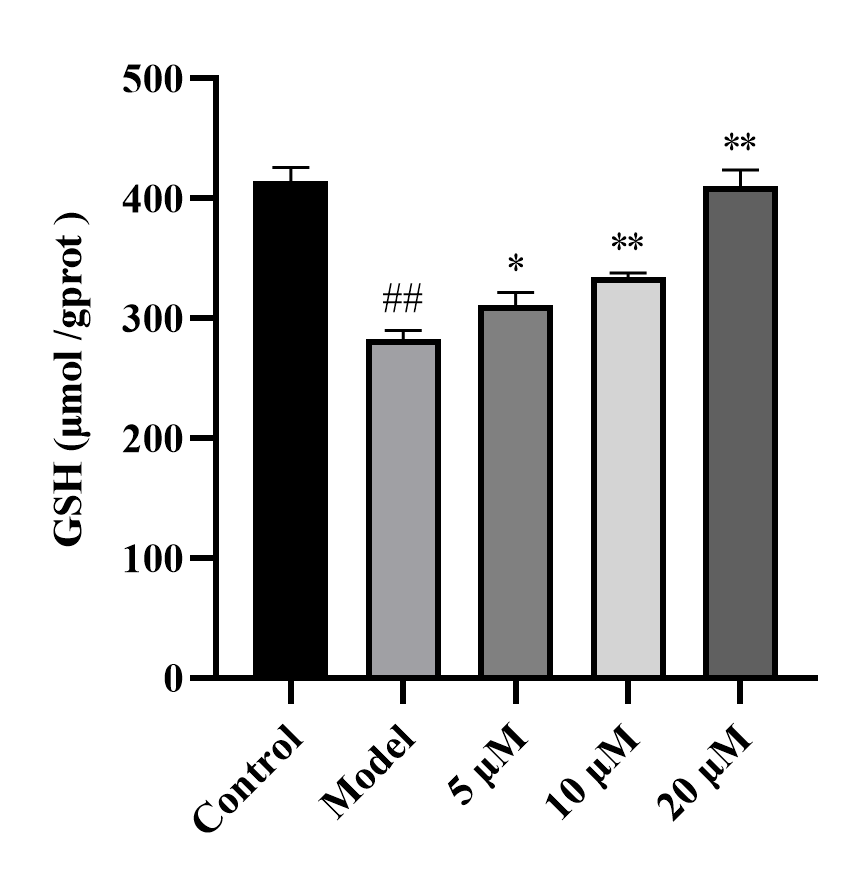

Supplement: Supplementary file 1 [file DataSheet1.zip › Raw data/Cell lipid oxidation index/GSH.png]

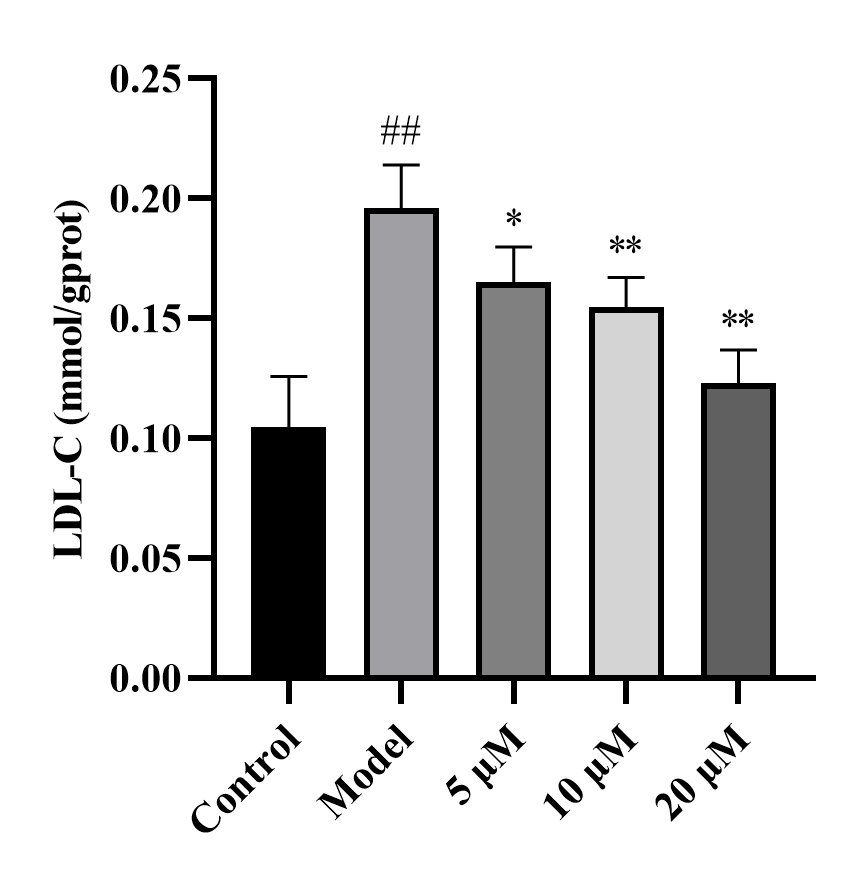

Supplement: Supplementary file 1 [file DataSheet1.zip › Raw data/Cell lipid oxidation index/LDL-C.png]

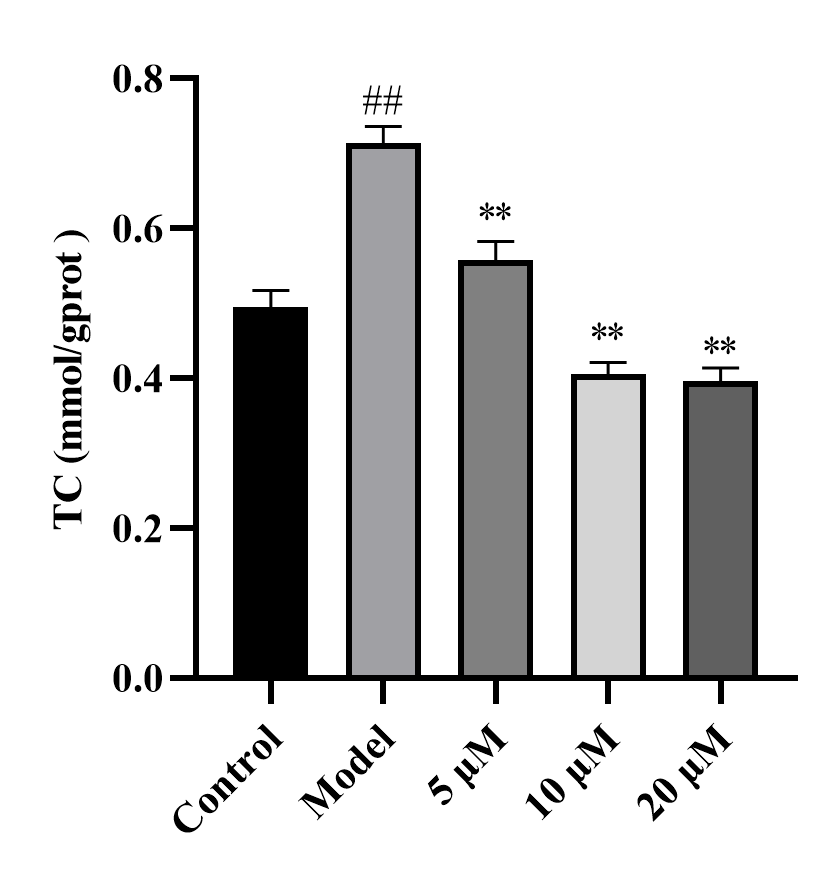

Supplement: Supplementary file 1 [file DataSheet1.zip › Raw data/Cell lipid oxidation index/TC.png]

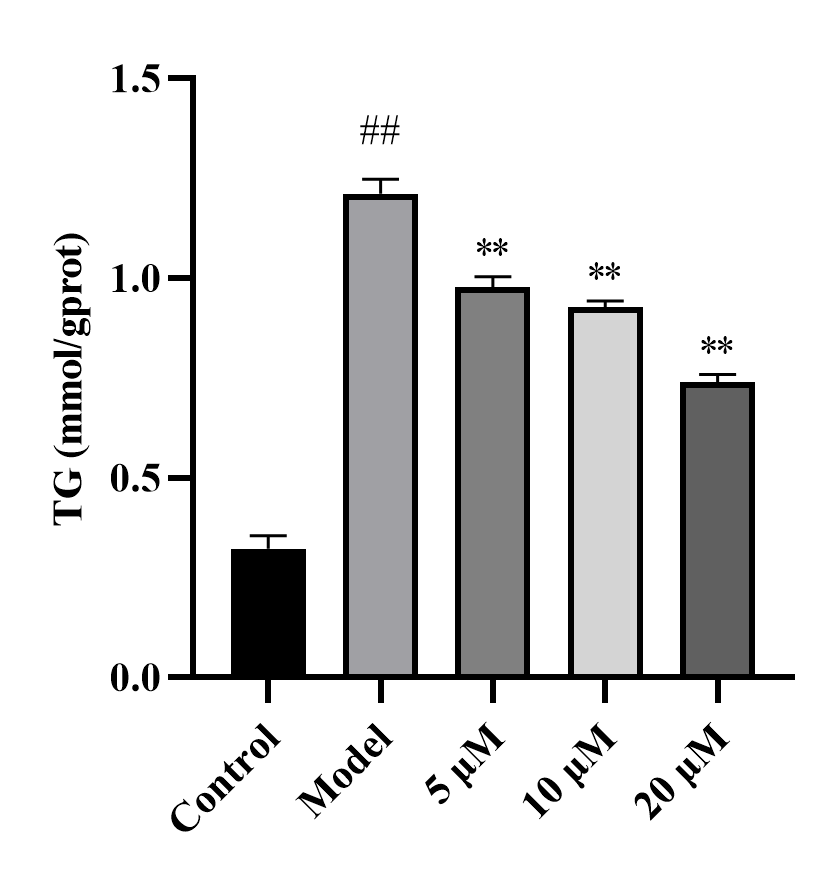

Supplement: Supplementary file 1 [file DataSheet1.zip › Raw data/Cell lipid oxidation index/TG.png]

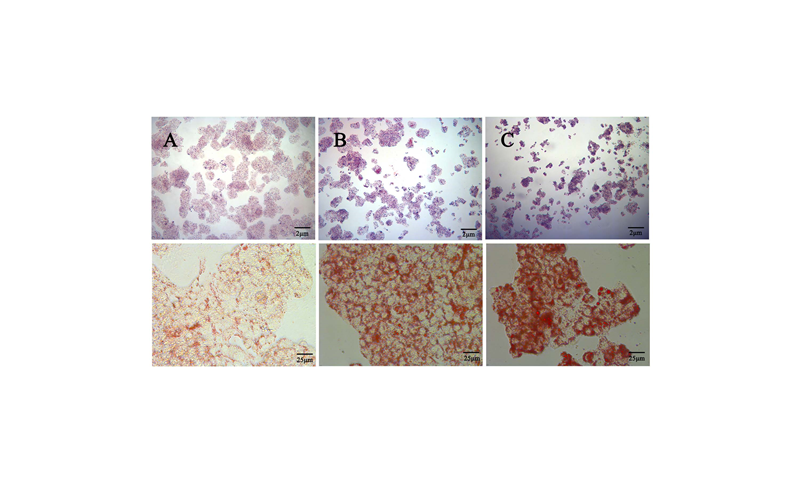

Supplement: Supplementary file 1 [file DataSheet1.zip › Raw data/Cell oil red O staining/Oleic acid - Oil red O.png]

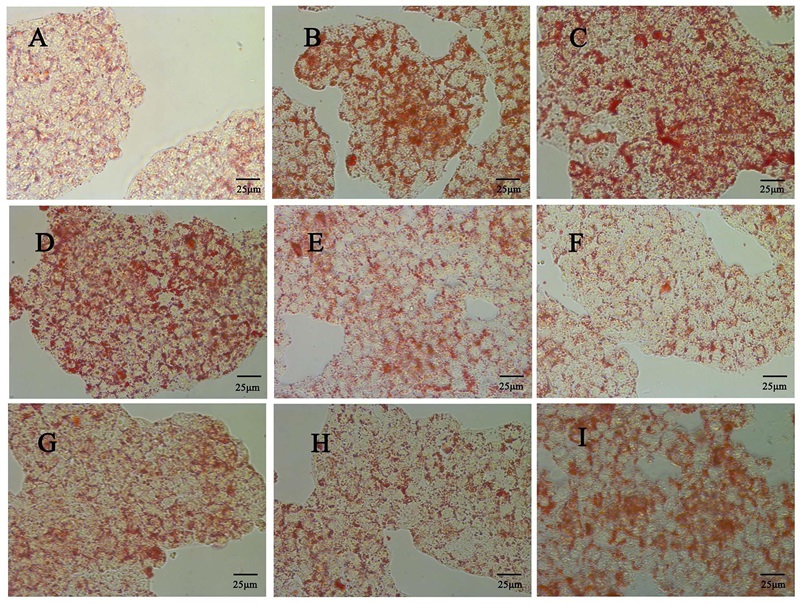

Supplement: Supplementary file 1 [file DataSheet1.zip › Raw data/Cell oil red O staining/Quercetin - Oil red O.jpg]

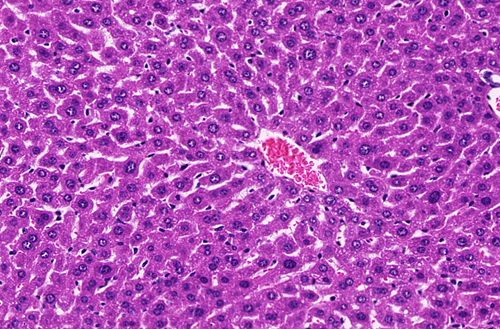

Supplement: Supplementary file 1 [file DataSheet1.zip › Raw data/HE/140.png]

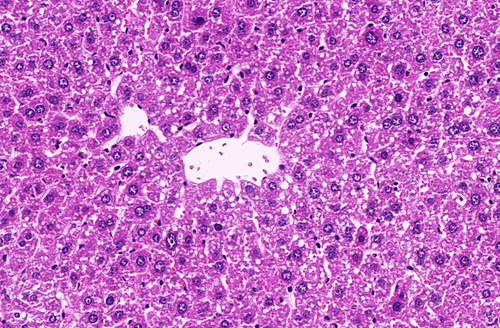

Supplement: Supplementary file 1 [file DataSheet1.zip › Raw data/HE/35.png]

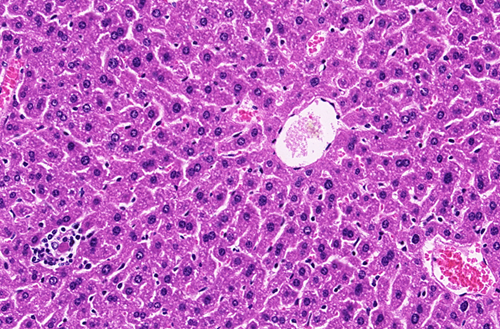

Supplement: Supplementary file 1 [file DataSheet1.zip › Raw data/HE/70.png]

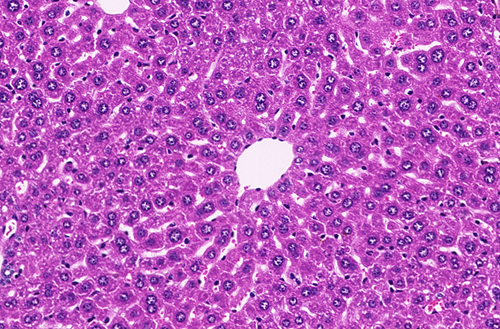

Supplement: Supplementary file 1 [file DataSheet1.zip › Raw data/HE/control.png]

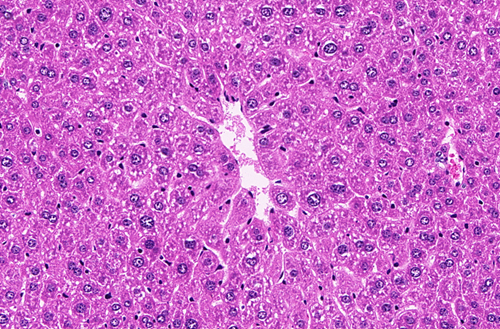

Supplement: Supplementary file 1 [file DataSheet1.zip › Raw data/HE/Metformin.png]

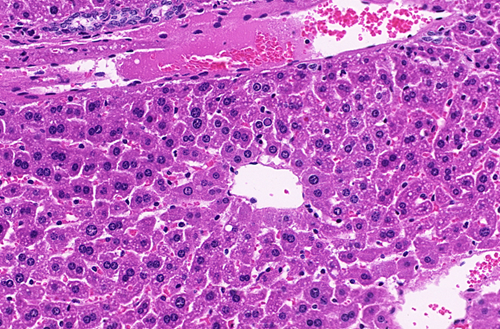

Supplement: Supplementary file 1 [file DataSheet1.zip › Raw data/HE/model.png]

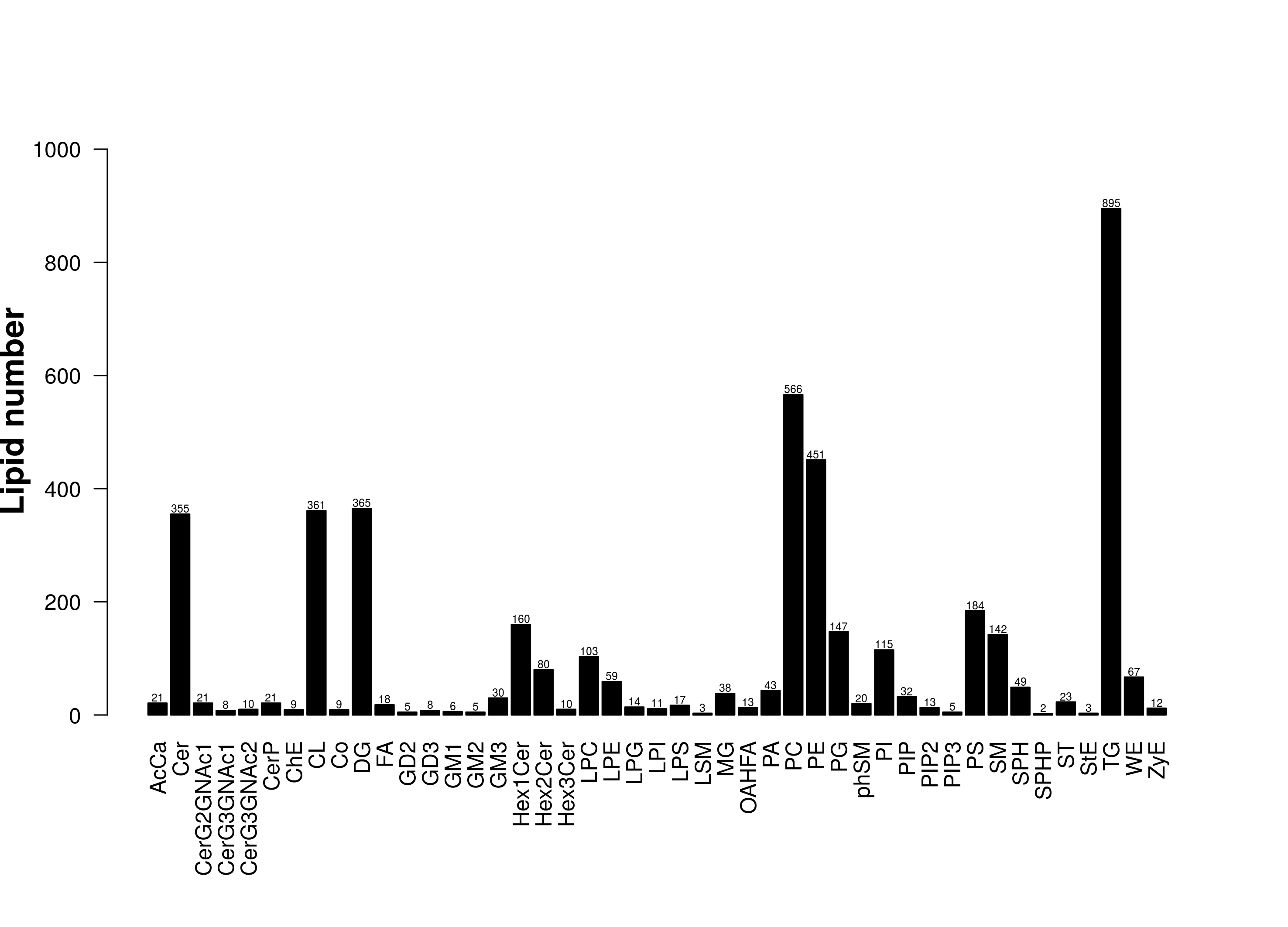

Supplement: Supplementary file 1 [file DataSheet1.zip › Raw data/Lipidomics/LipidNumber.png]

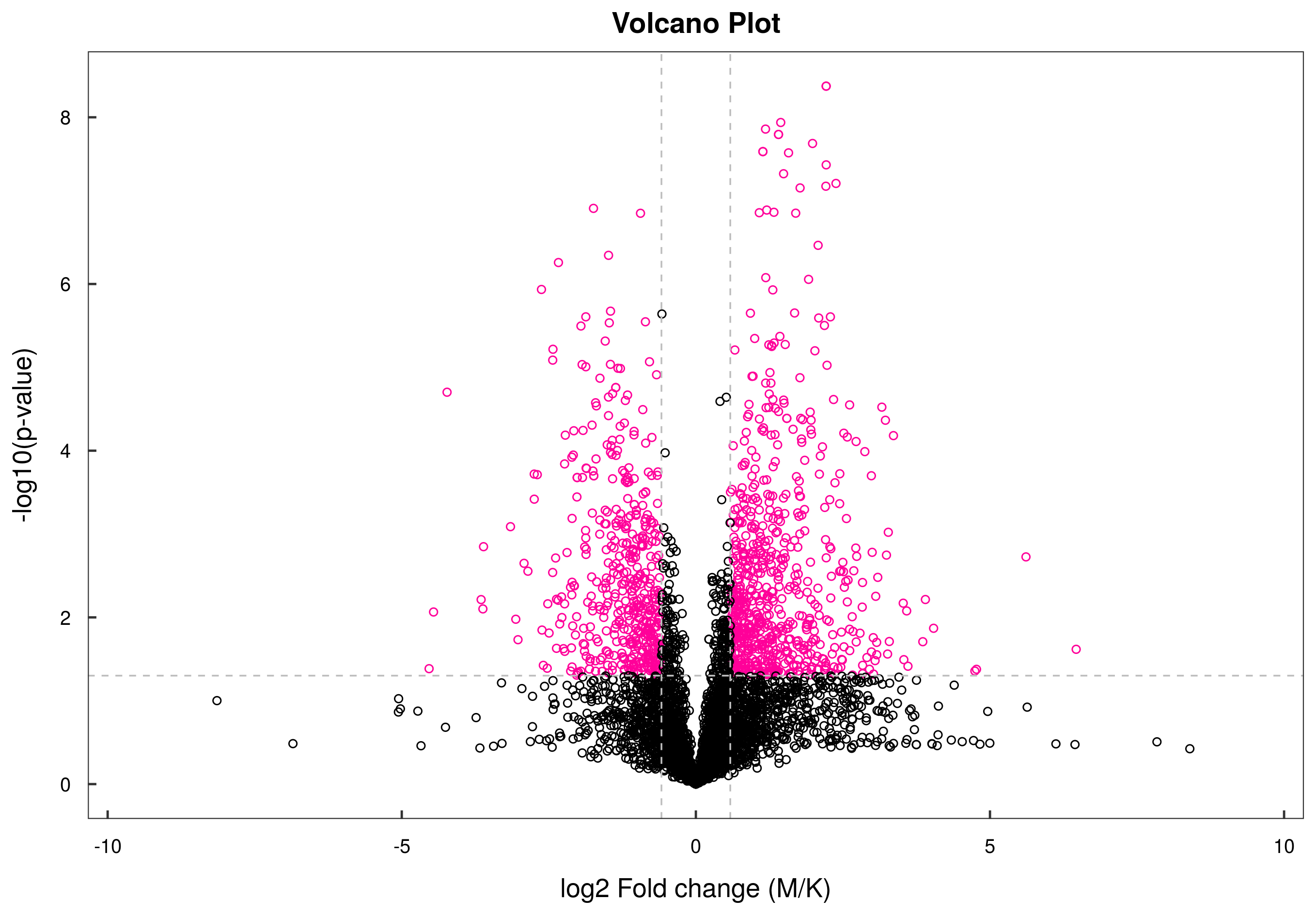

Supplement: Supplementary file 1 [file DataSheet1.zip › Raw data/Lipidomics/MK.png]

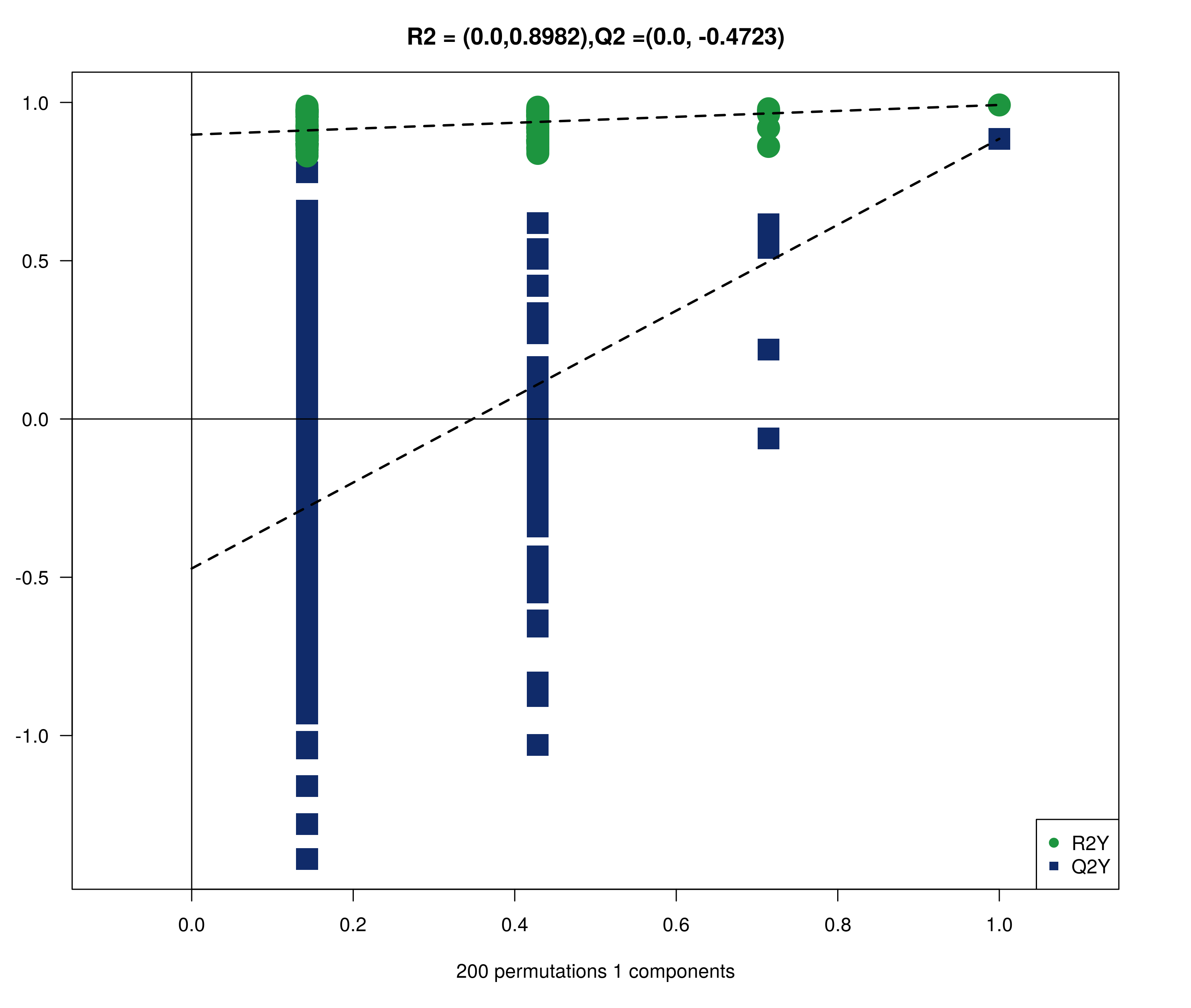

Supplement: Supplementary file 1 [file DataSheet1.zip › Raw data/Lipidomics/M_vs_K-OPLS-DA-Permutation.png]

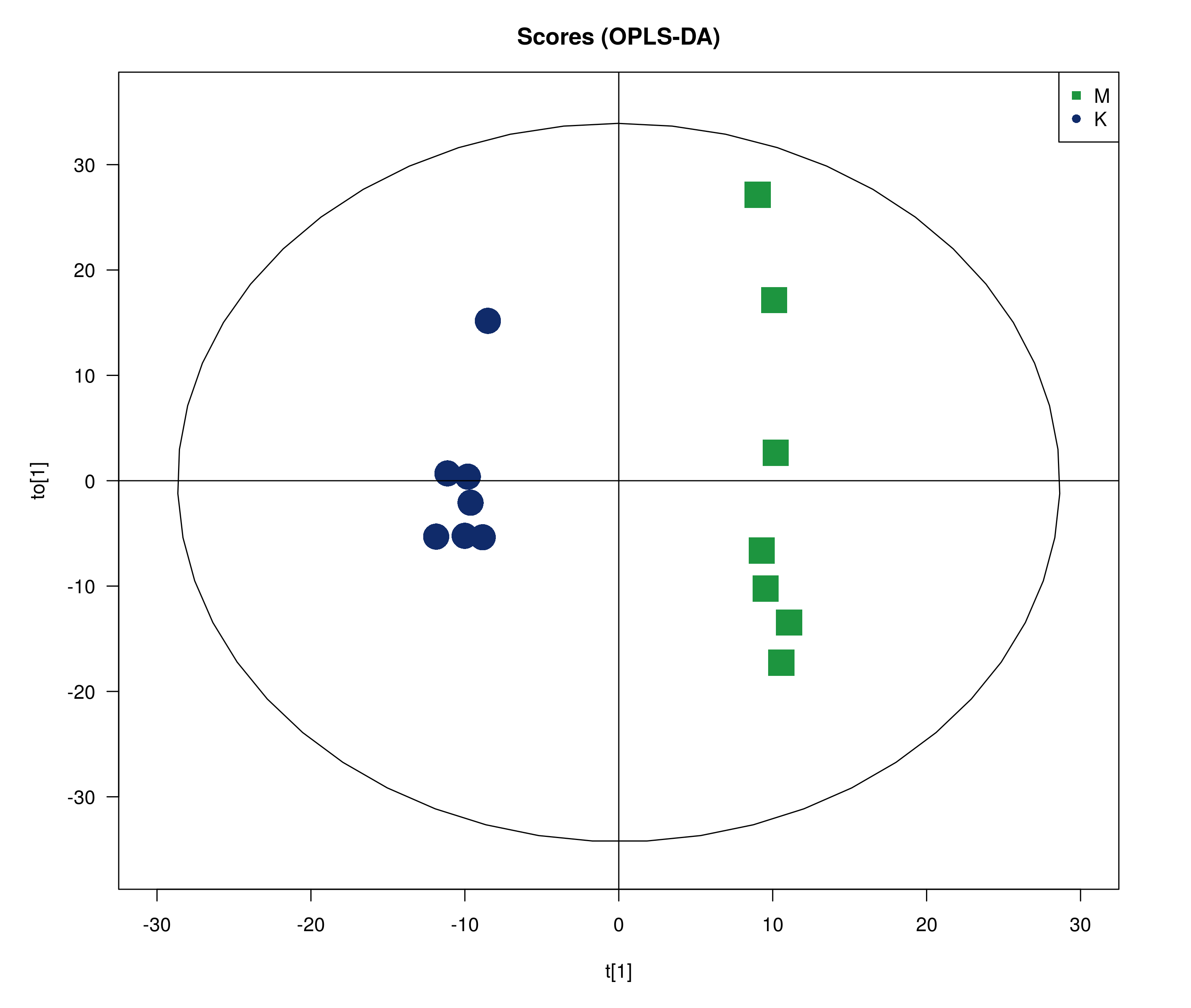

Supplement: Supplementary file 1 [file DataSheet1.zip › Raw data/Lipidomics/M_vs_K-OPLS-DA.png]

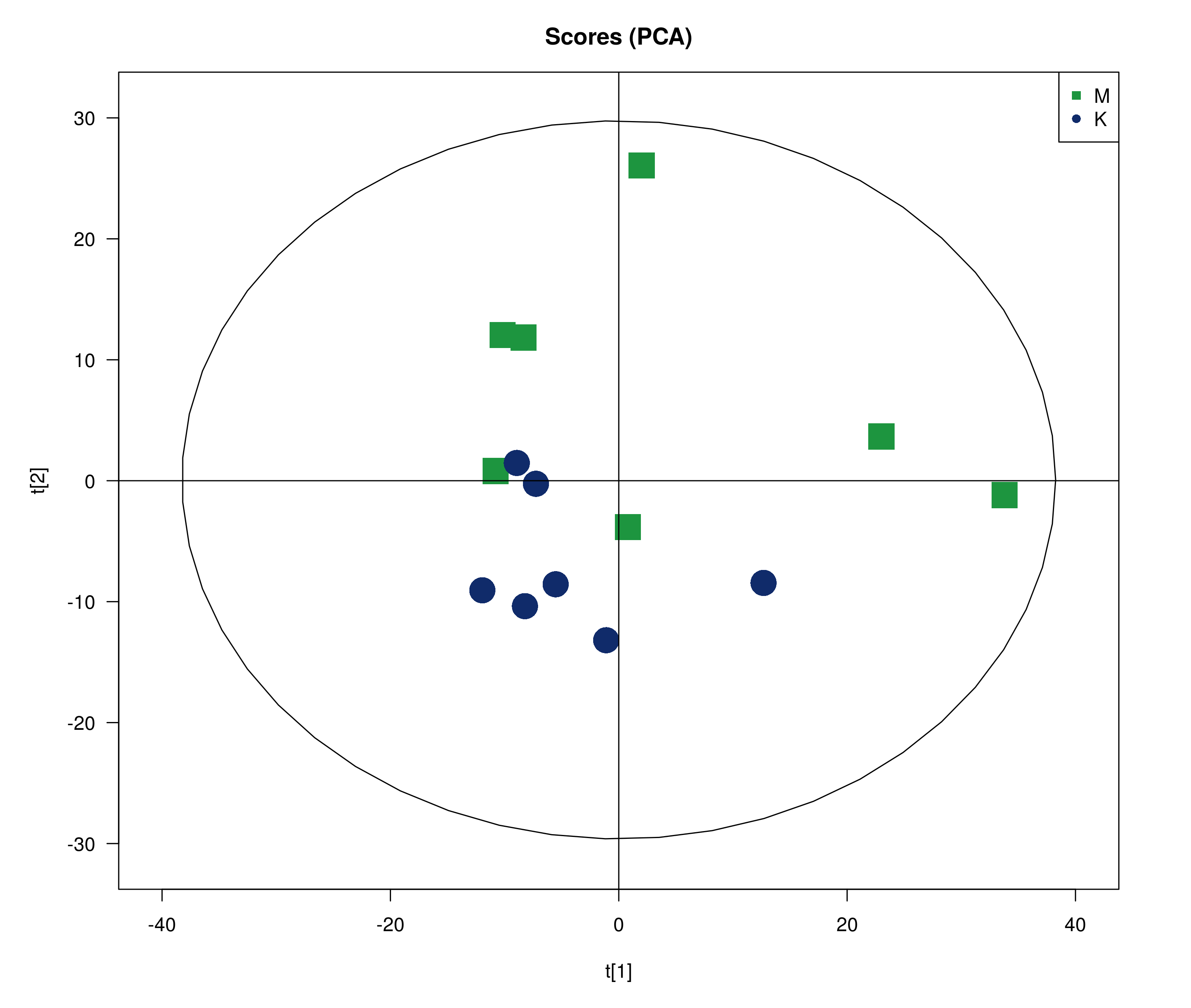

Supplement: Supplementary file 1 [file DataSheet1.zip › Raw data/Lipidomics/M_vs_K-PCA.png]

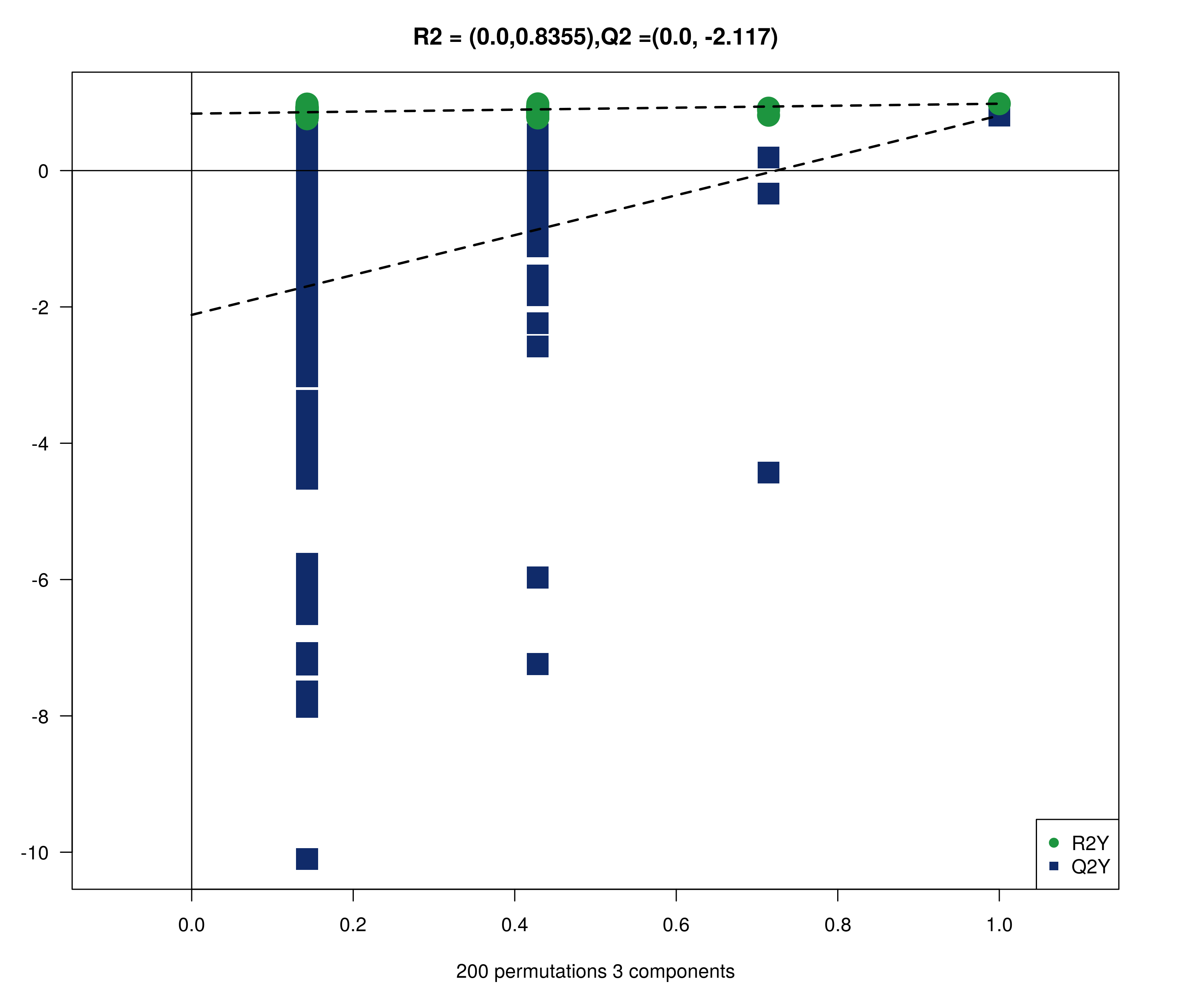

Supplement: Supplementary file 1 [file DataSheet1.zip › Raw data/Lipidomics/M_vs_K-PLS-DA-Permutation.png]

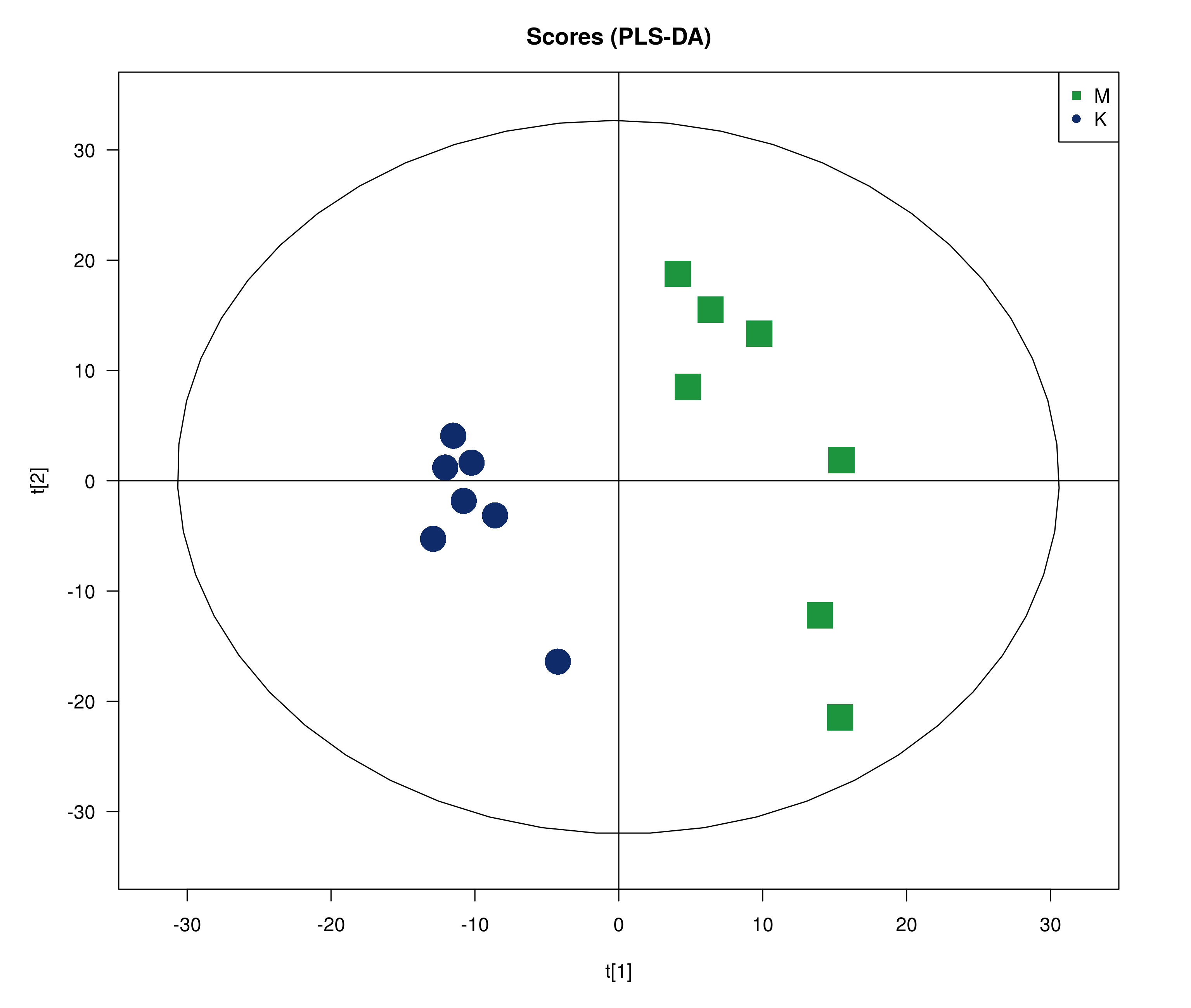

Supplement: Supplementary file 1 [file DataSheet1.zip › Raw data/Lipidomics/M_vs_K-PLS-DA.png]

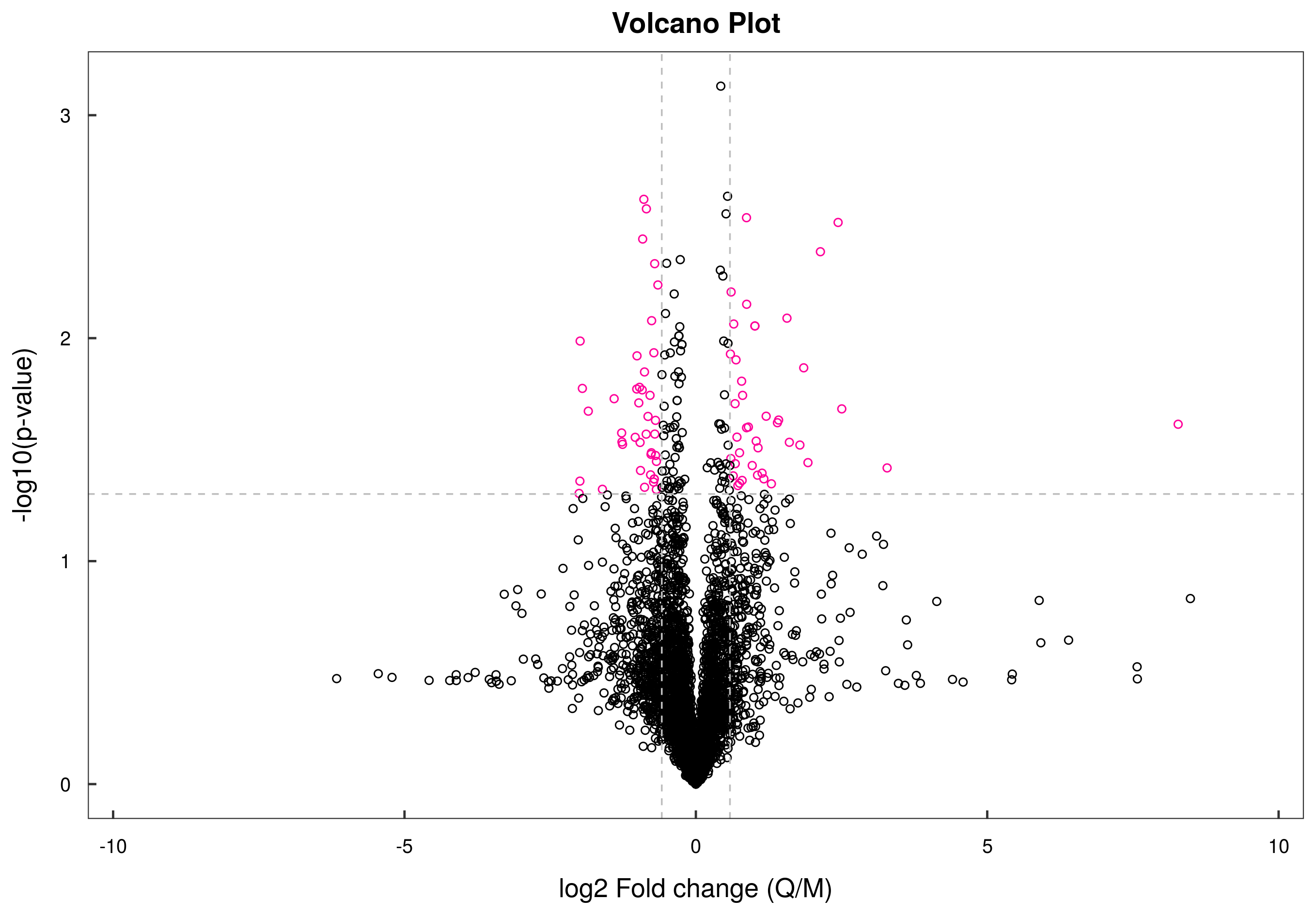

Supplement: Supplementary file 1 [file DataSheet1.zip › Raw data/Lipidomics/QM.png]

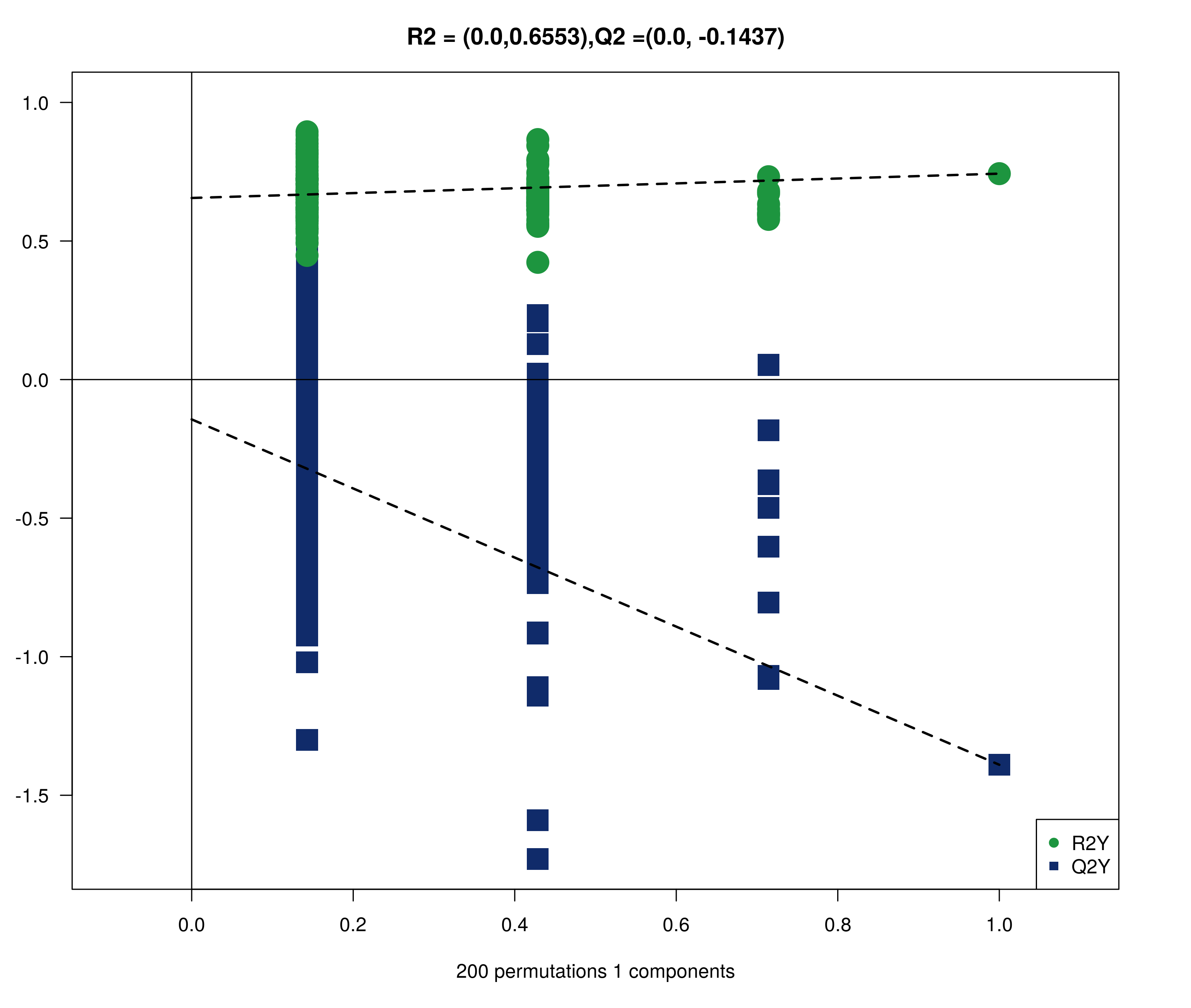

Supplement: Supplementary file 1 [file DataSheet1.zip › Raw data/Lipidomics/Q_vs_M-OPLS-DA-Permutation.png]

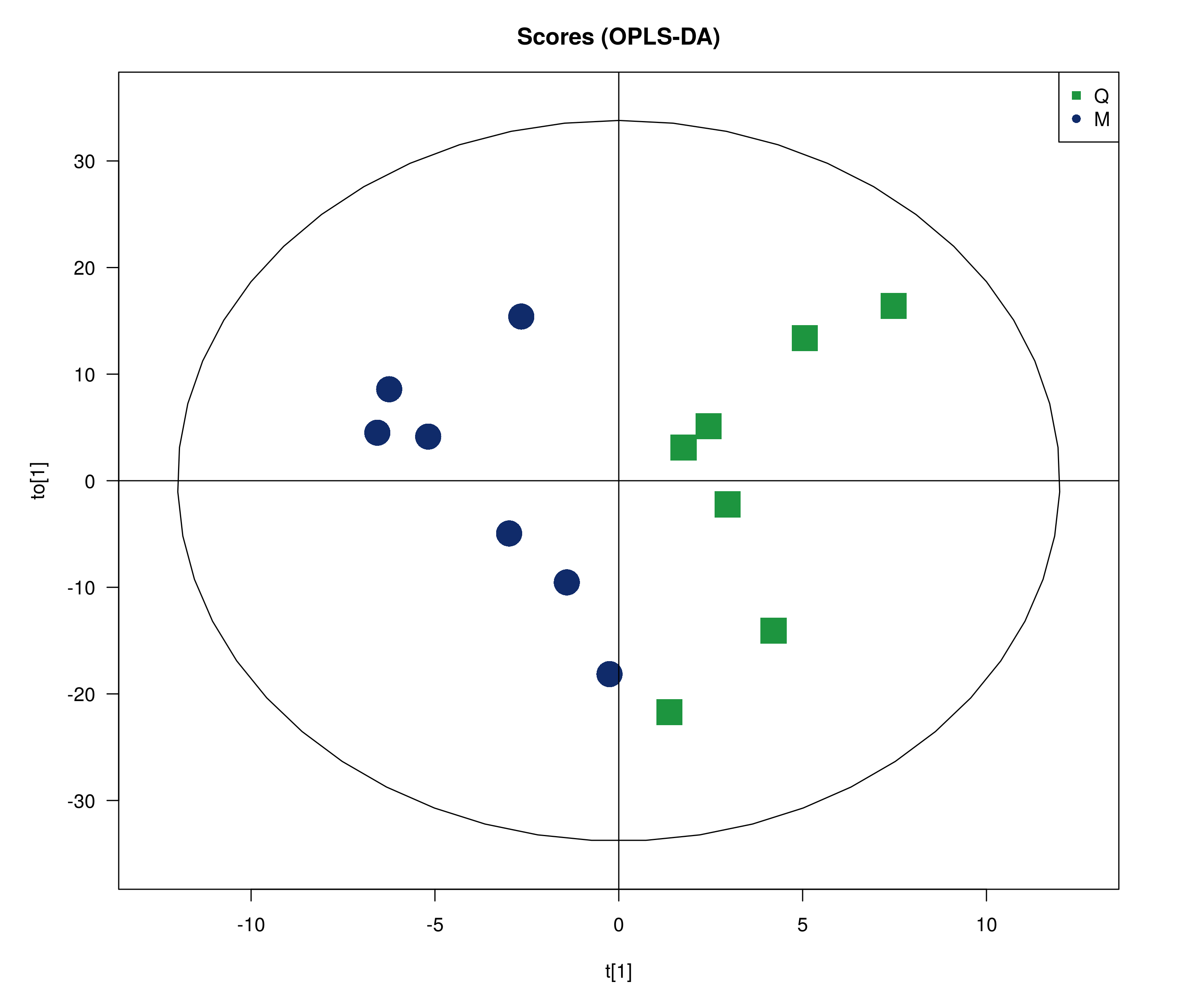

Supplement: Supplementary file 1 [file DataSheet1.zip › Raw data/Lipidomics/Q_vs_M-OPLS-DA.png]

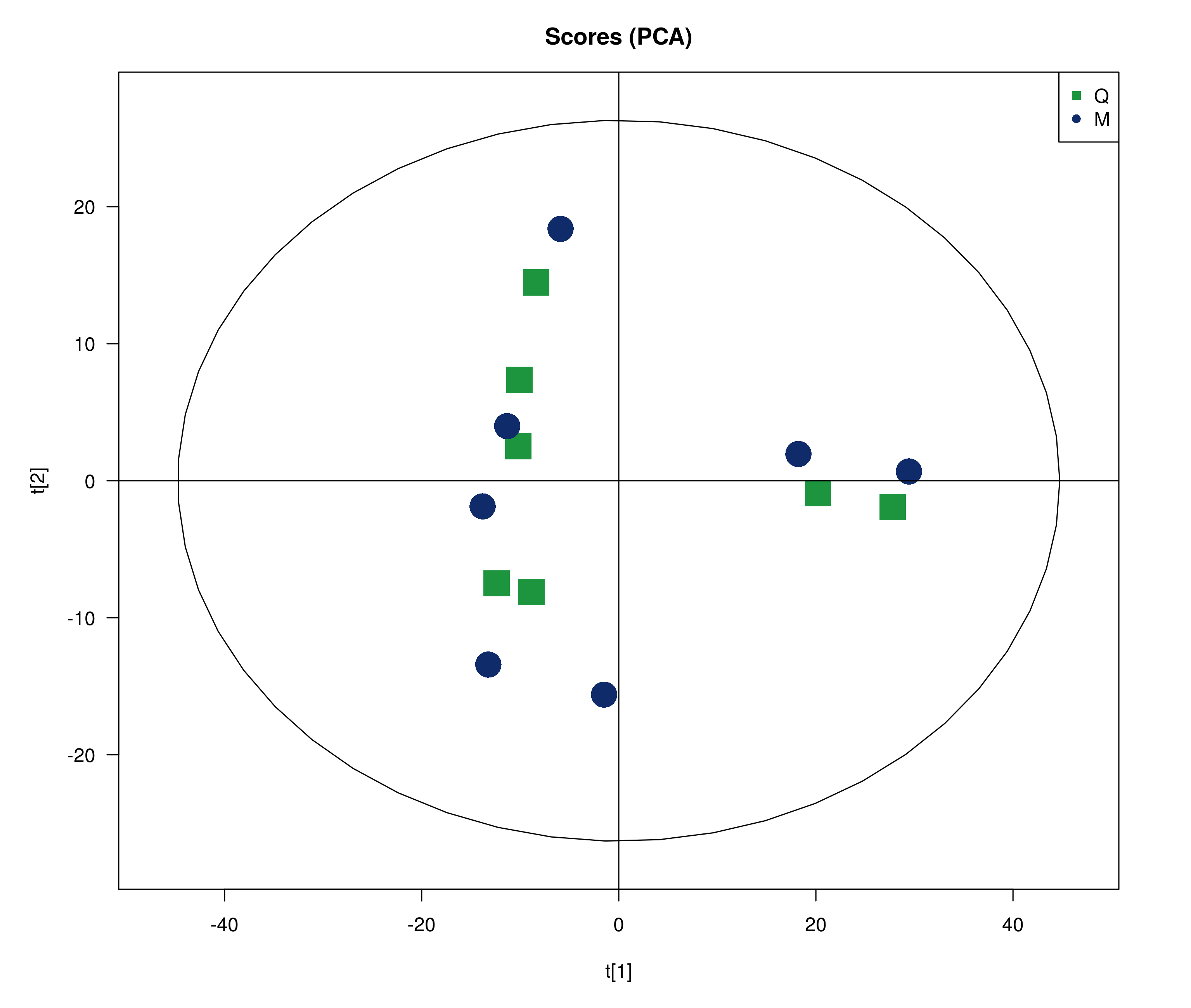

Supplement: Supplementary file 1 [file DataSheet1.zip › Raw data/Lipidomics/Q_vs_M-PCA.png]

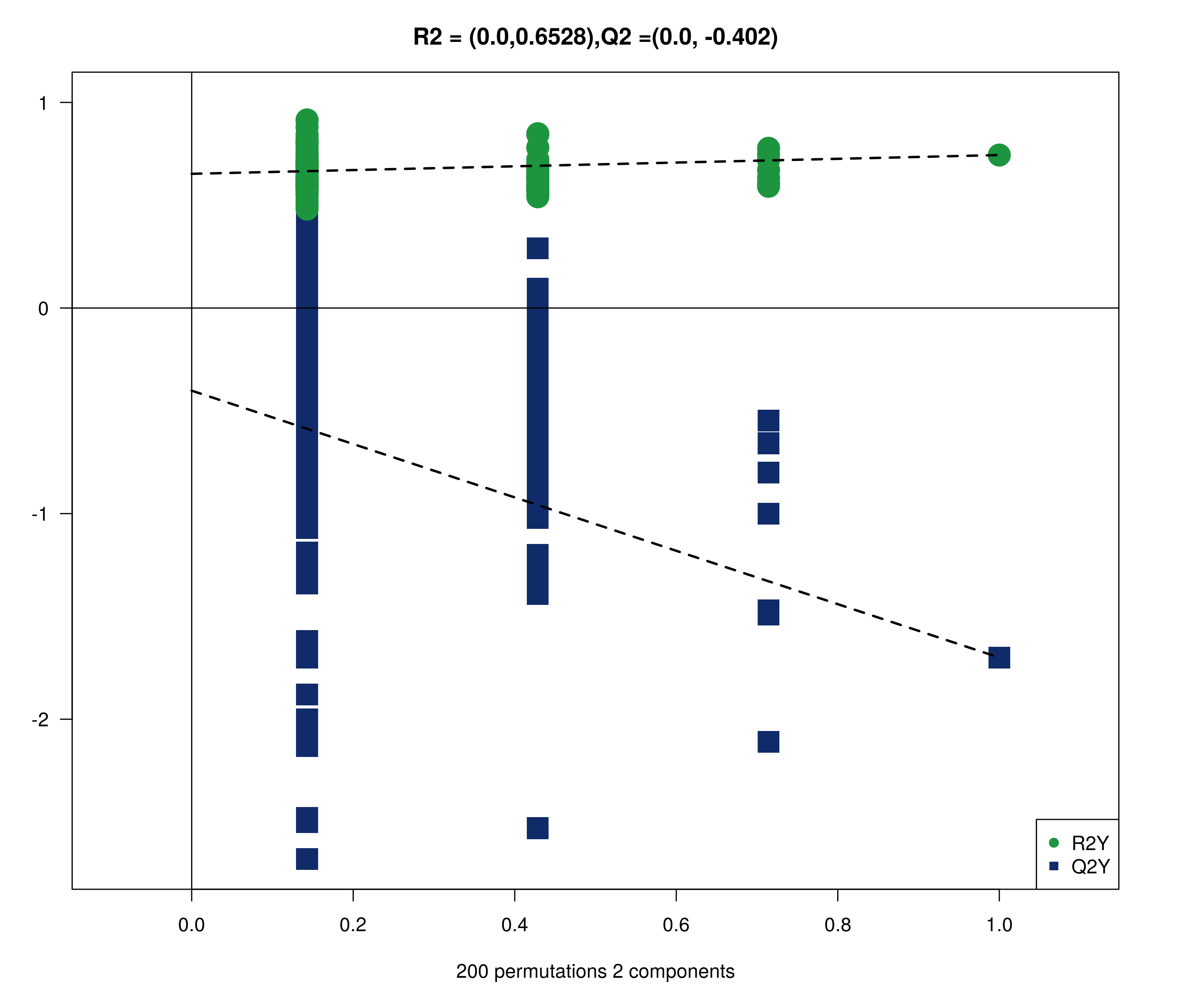

Supplement: Supplementary file 1 [file DataSheet1.zip › Raw data/Lipidomics/Q_vs_M-PLS-DA-Permutation.png]

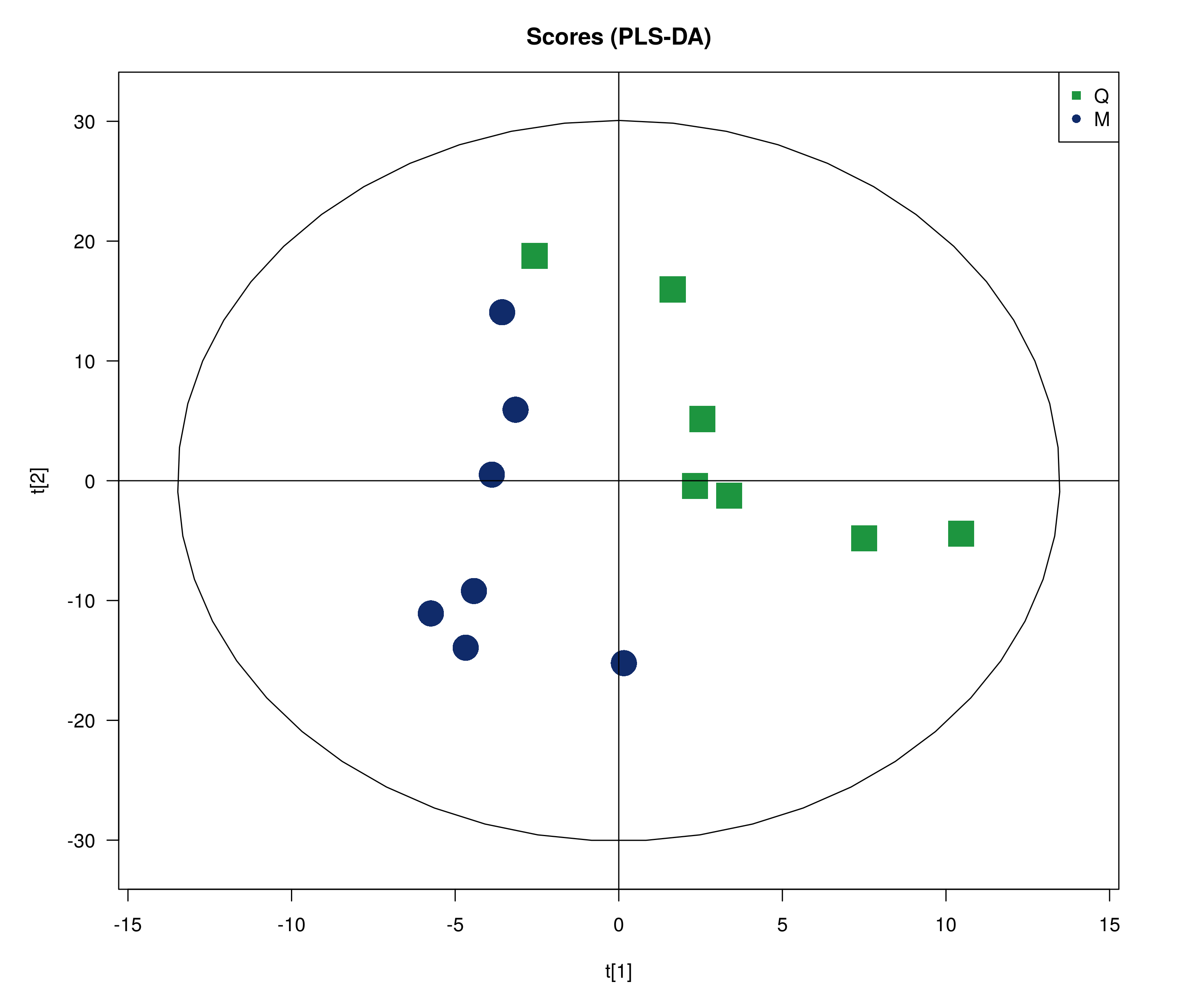

Supplement: Supplementary file 1 [file DataSheet1.zip › Raw data/Lipidomics/Q_vs_M-PLS-DA.png]

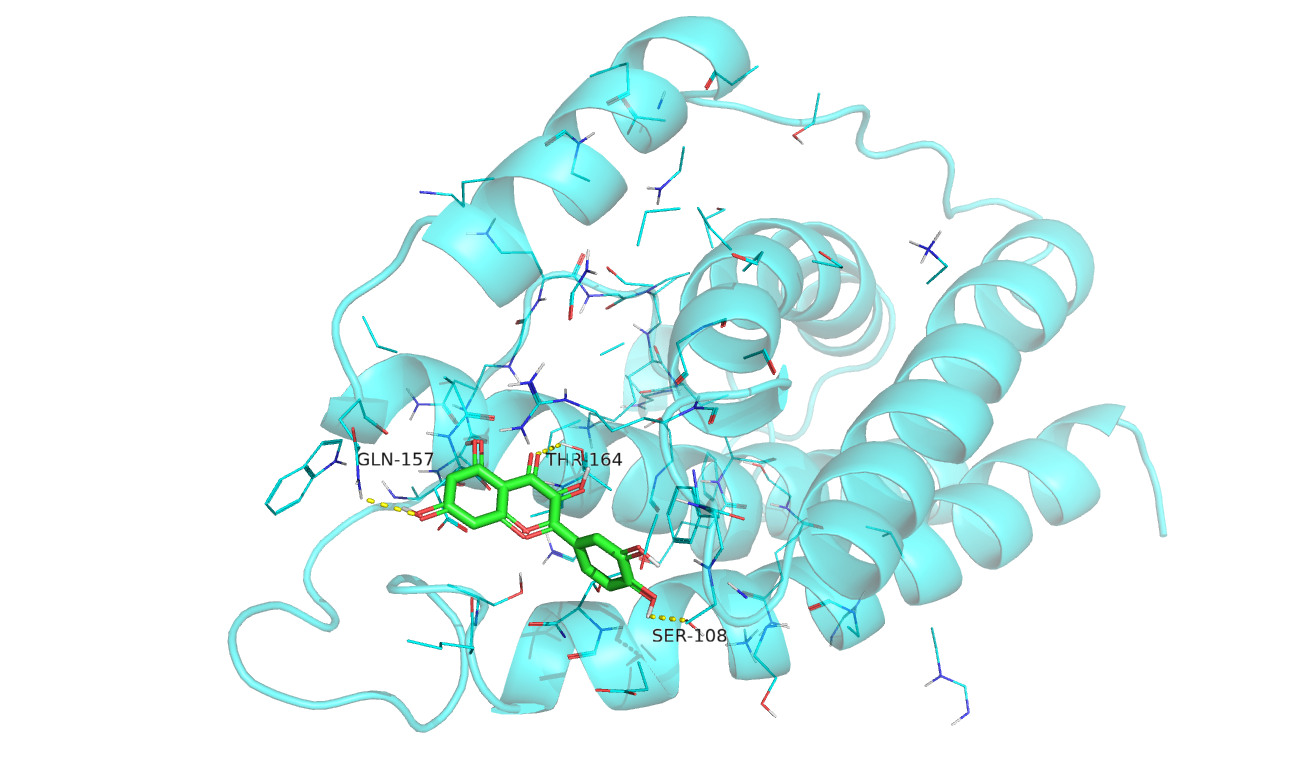

Supplement: Supplementary file 1 [file DataSheet1.zip › Raw data/Molecular docking/Molecular docking/visualization/quercetin IL6/quercetin IL6.png]

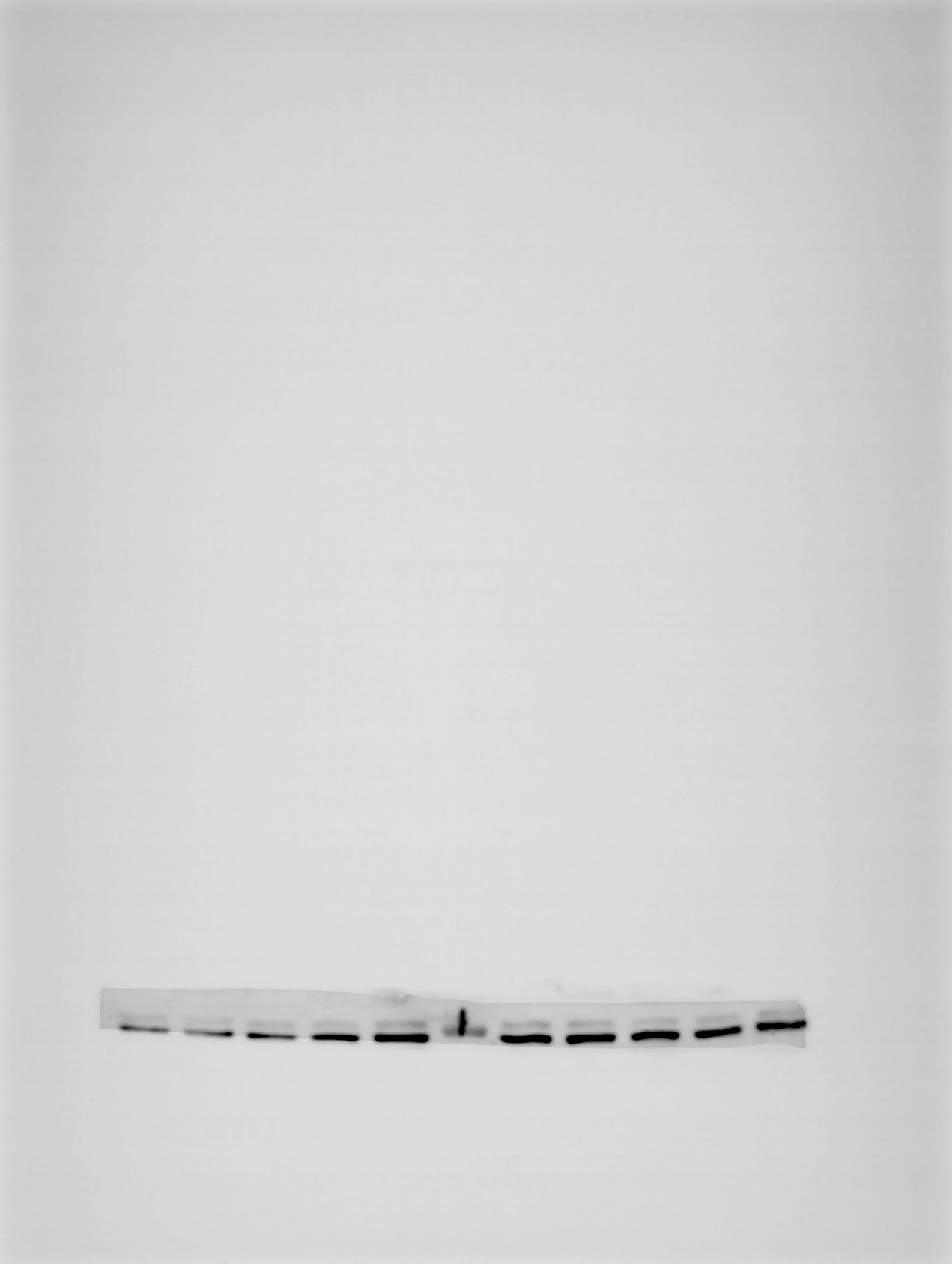

Supplement: Supplementary file 1 [file DataSheet1.zip › Raw data/WB data/Pictures/ERK/ERK-5+M.png]

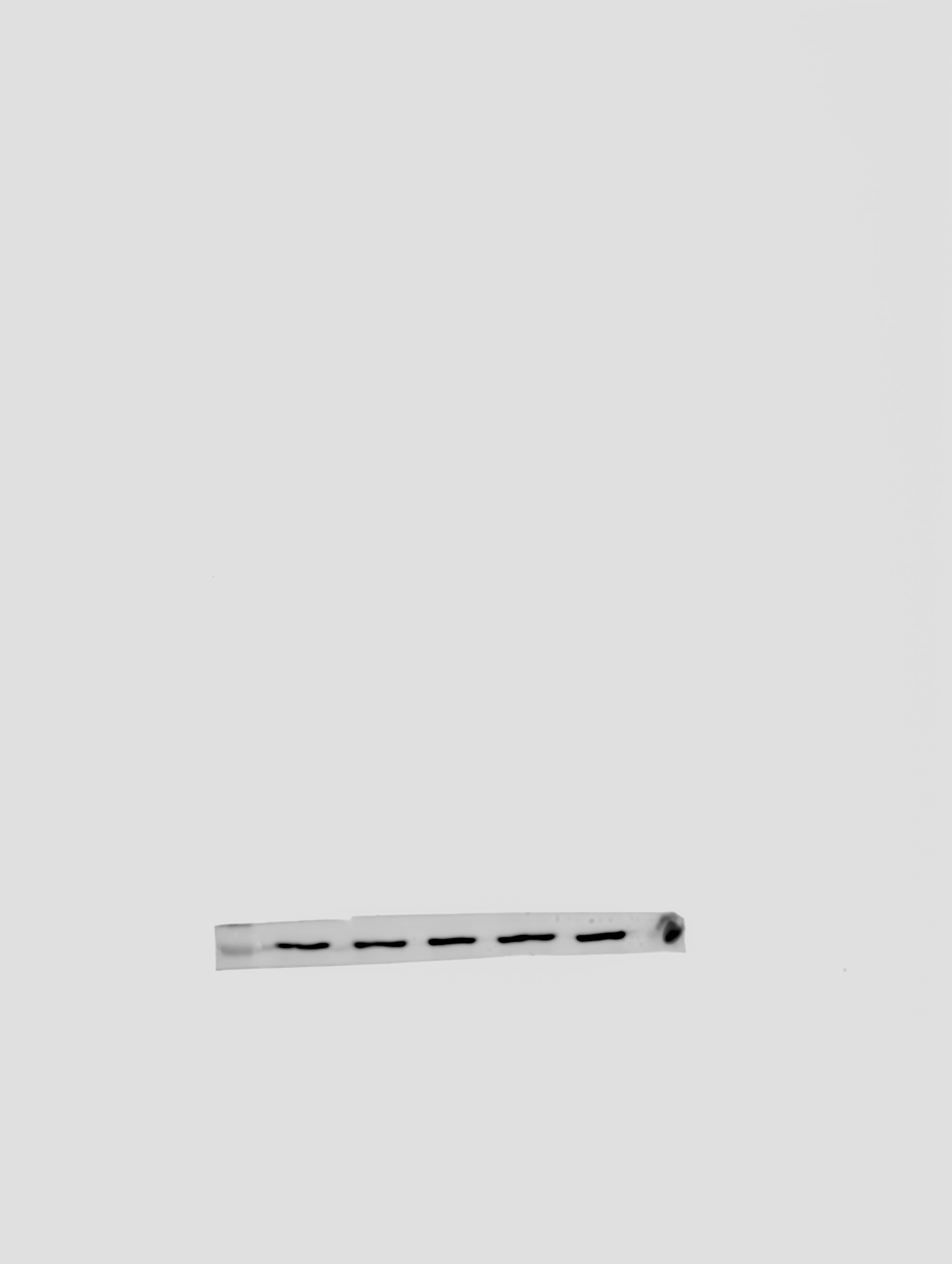

Supplement: Supplementary file 1 [file DataSheet1.zip › Raw data/WB data/Pictures/GPX4/GAPDH-1+M.png]

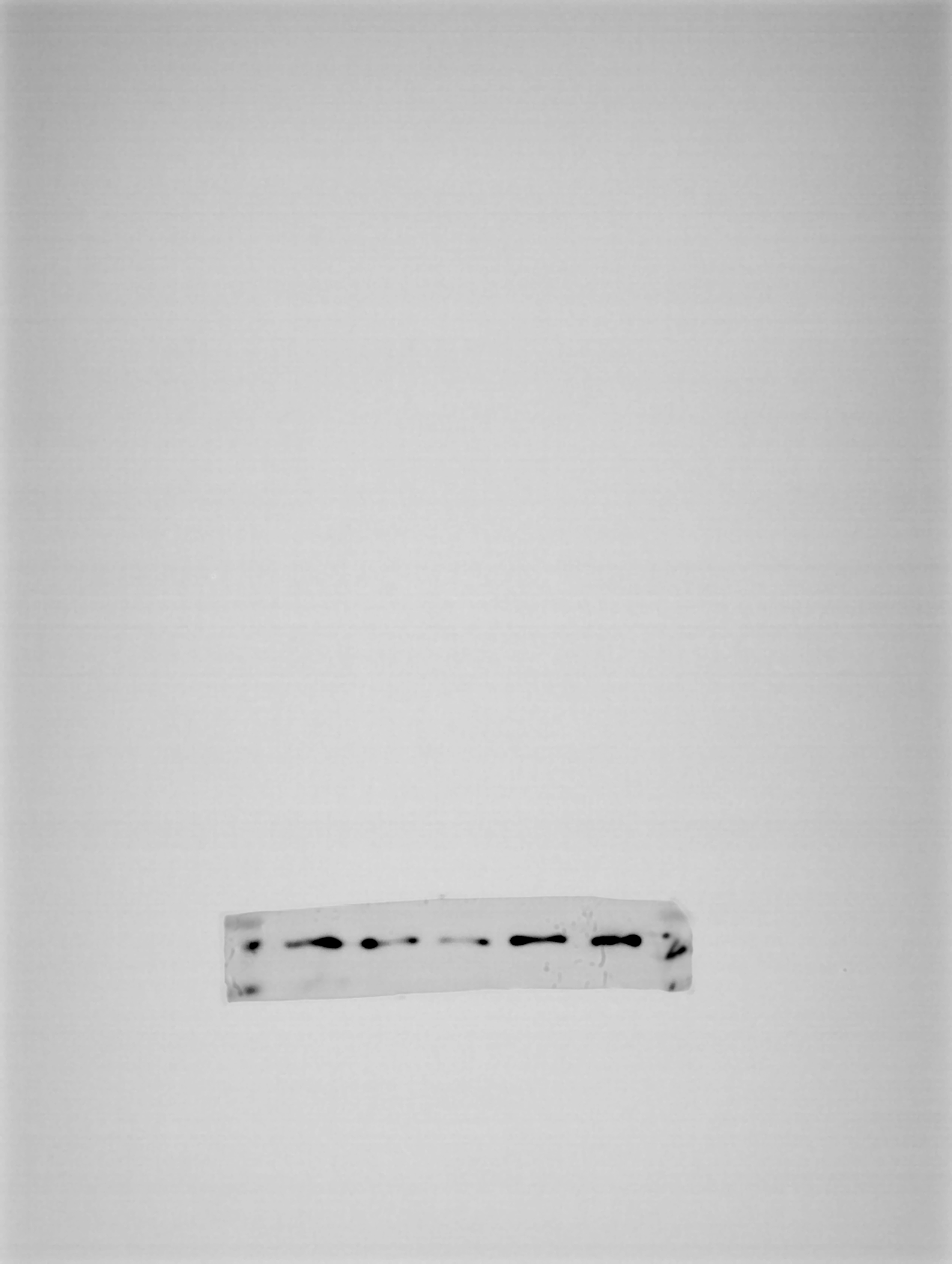

Supplement: Supplementary file 1 [file DataSheet1.zip › Raw data/WB data/Pictures/GPX4/GPX4-1+M.png]

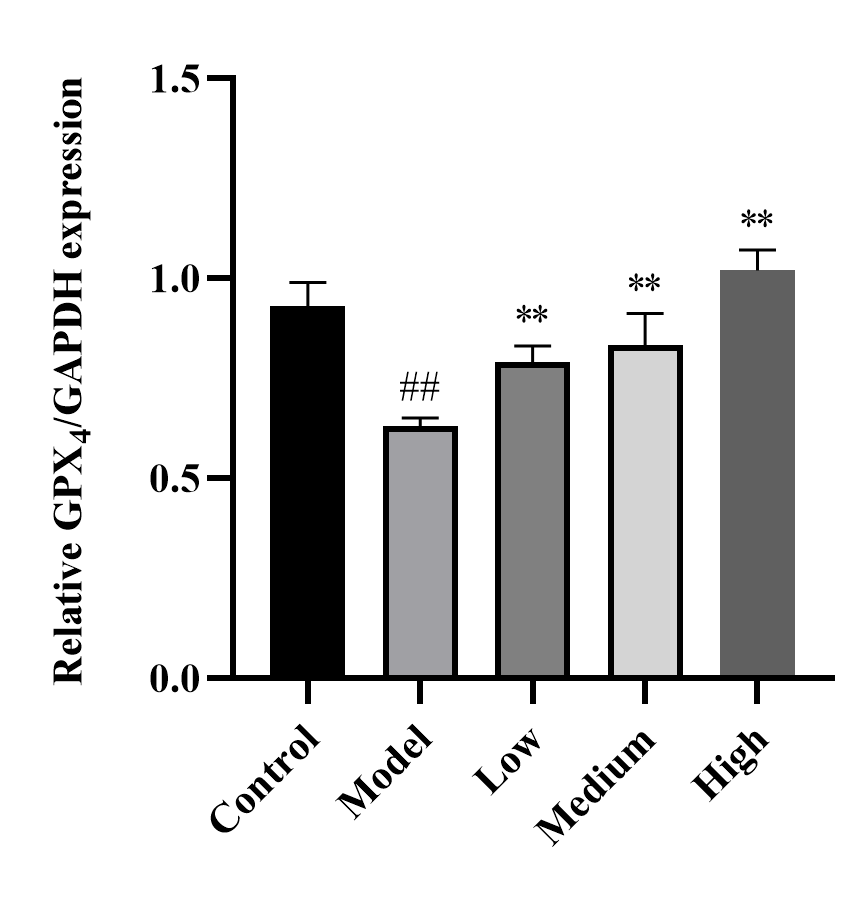

Supplement: Supplementary file 1 [file DataSheet1.zip › Raw data/WB data/Pictures/GPX4.png]

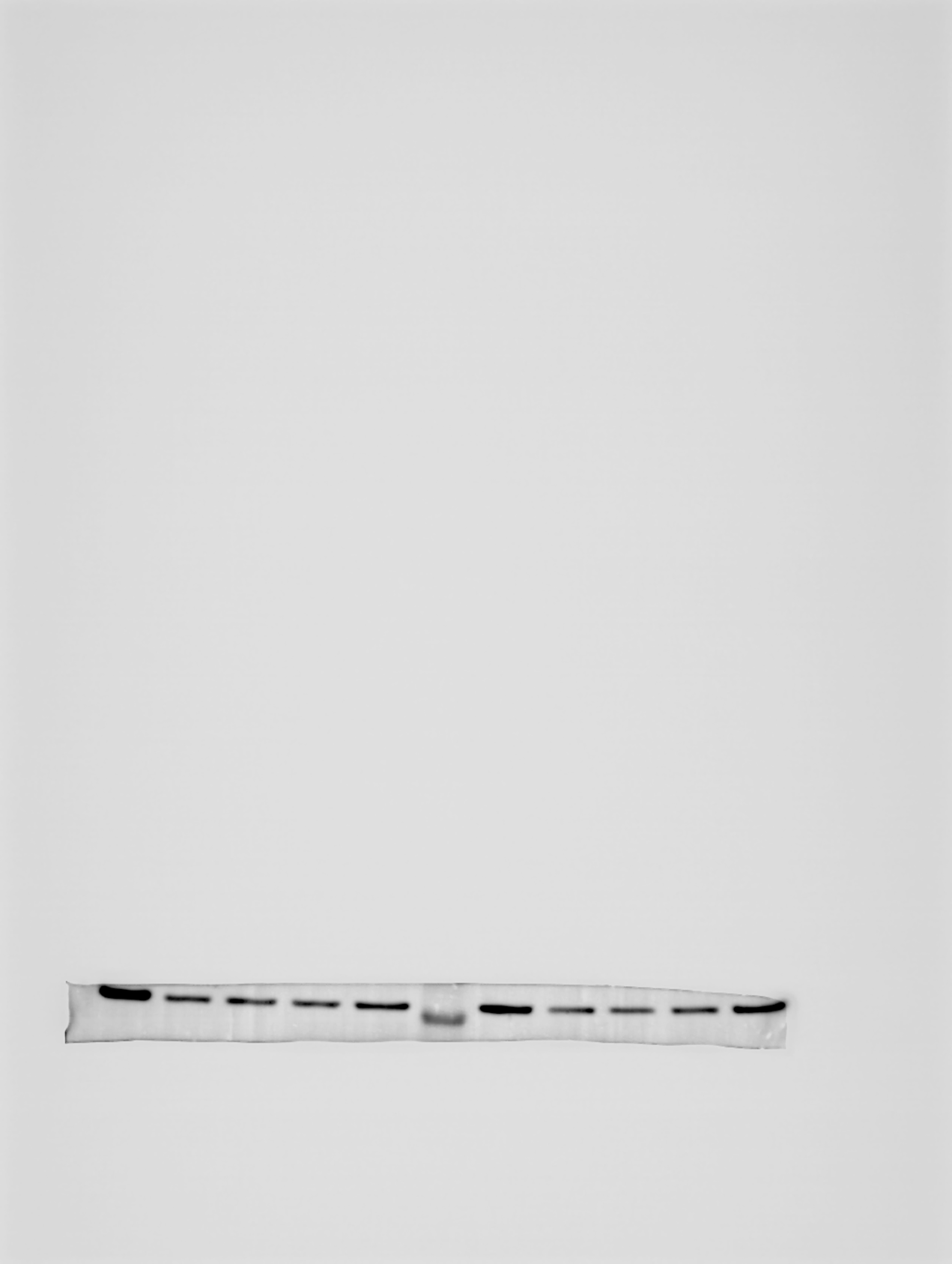

Supplement: Supplementary file 1 [file DataSheet1.zip › Raw data/WB data/Pictures/P-ERK/GAPDH-6+M.png]

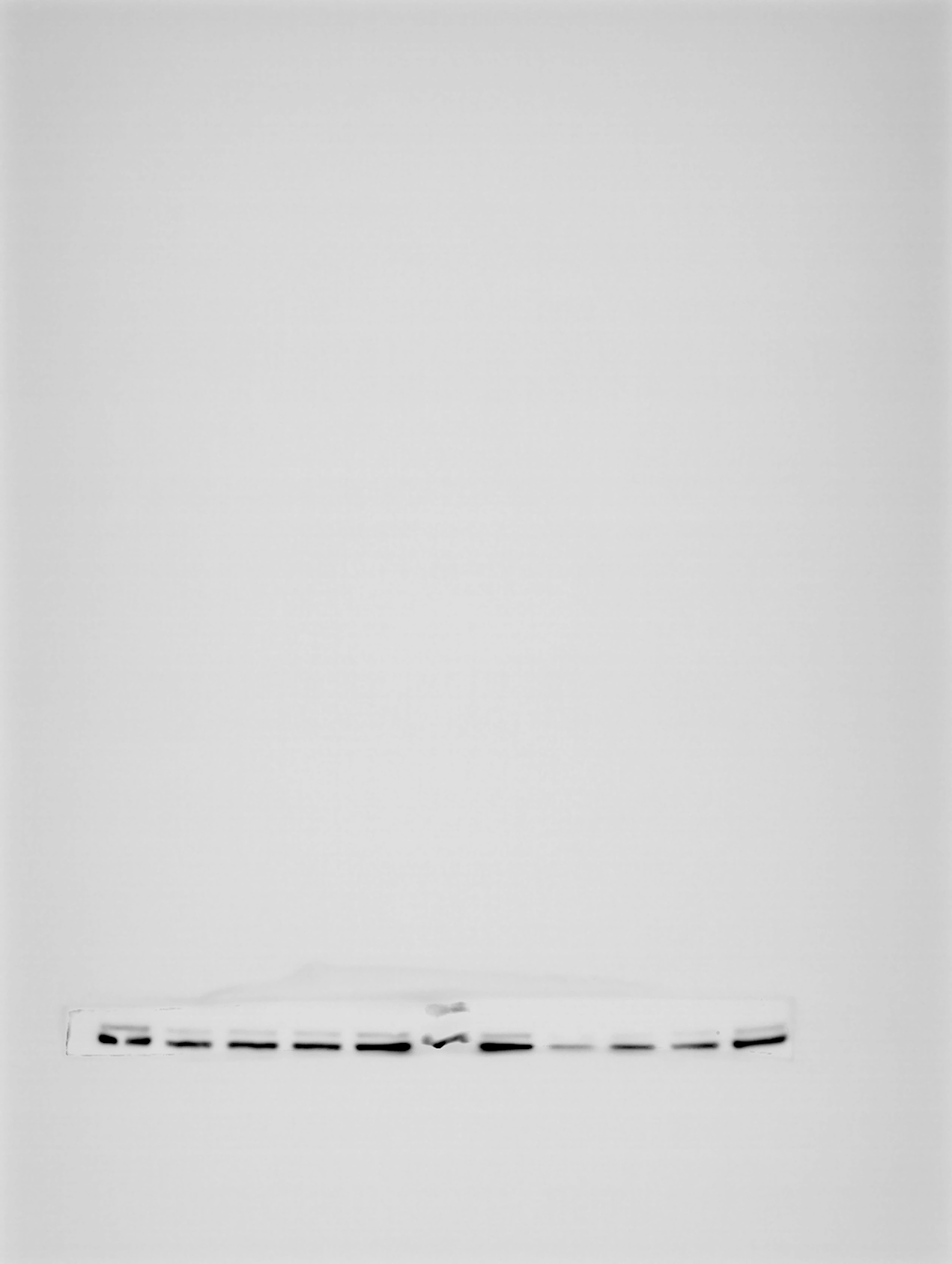

Supplement: Supplementary file 1 [file DataSheet1.zip › Raw data/WB data/Pictures/P-ERK/P-ERK-6+M.png]

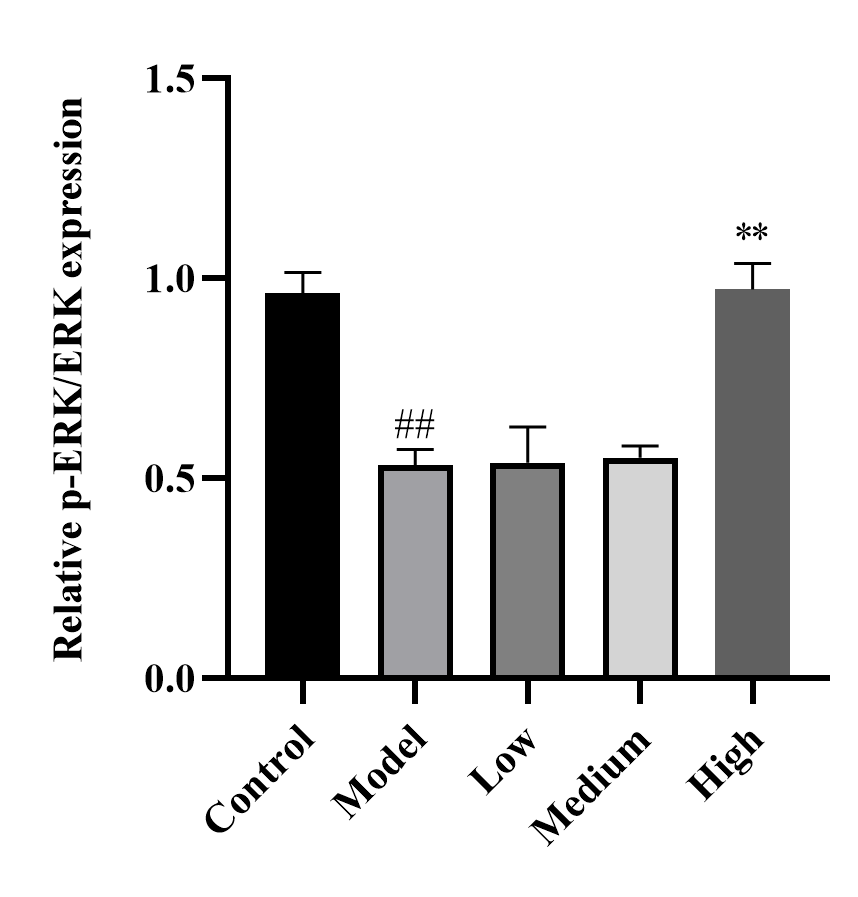

Supplement: Supplementary file 1 [file DataSheet1.zip › Raw data/WB data/Pictures/P-ERK_ERK.png]

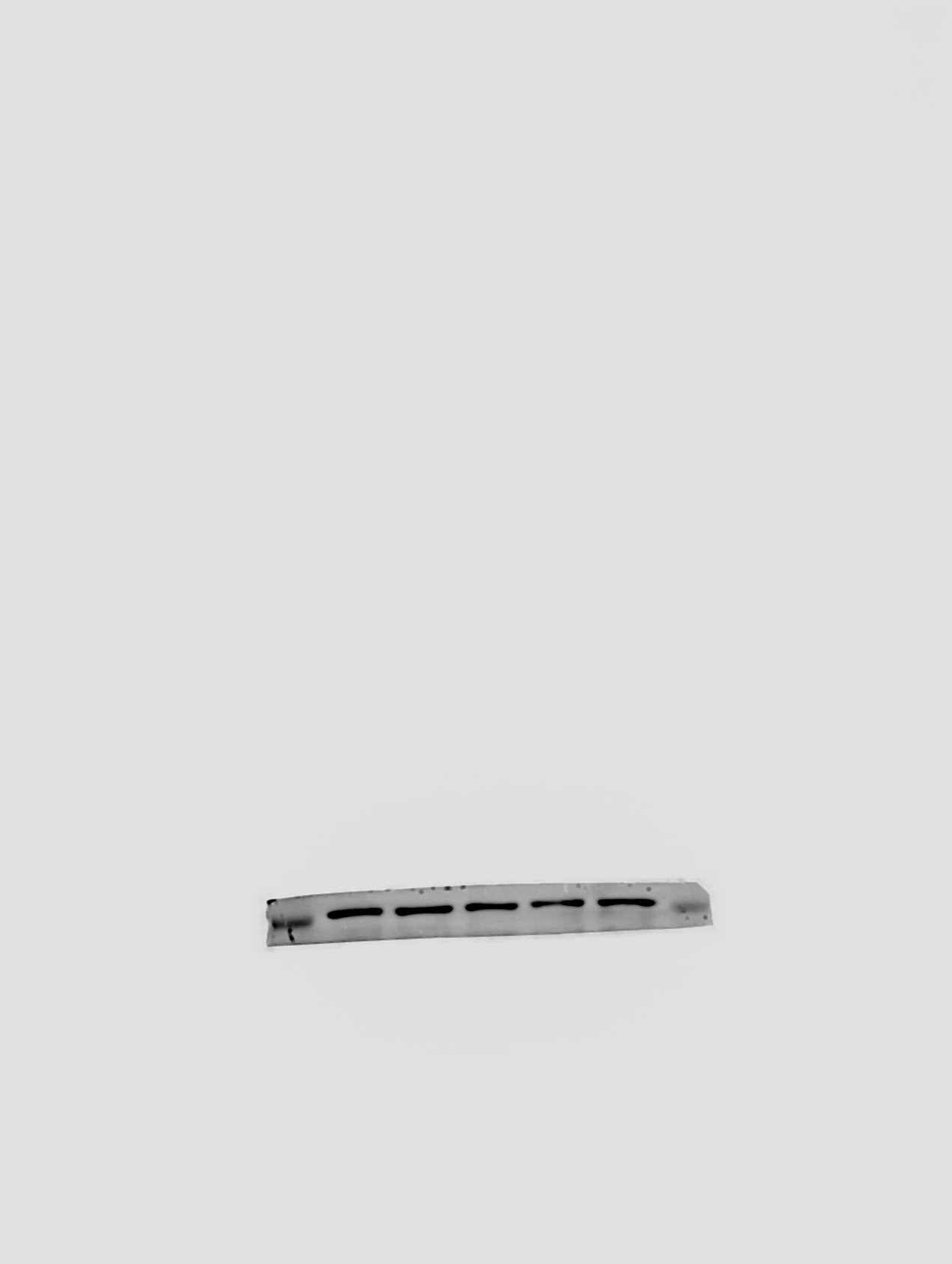

Supplement: Supplementary file 1 [file DataSheet1.zip › Raw data/WB data/Pictures/P-P38/GAPDH1+M.png]

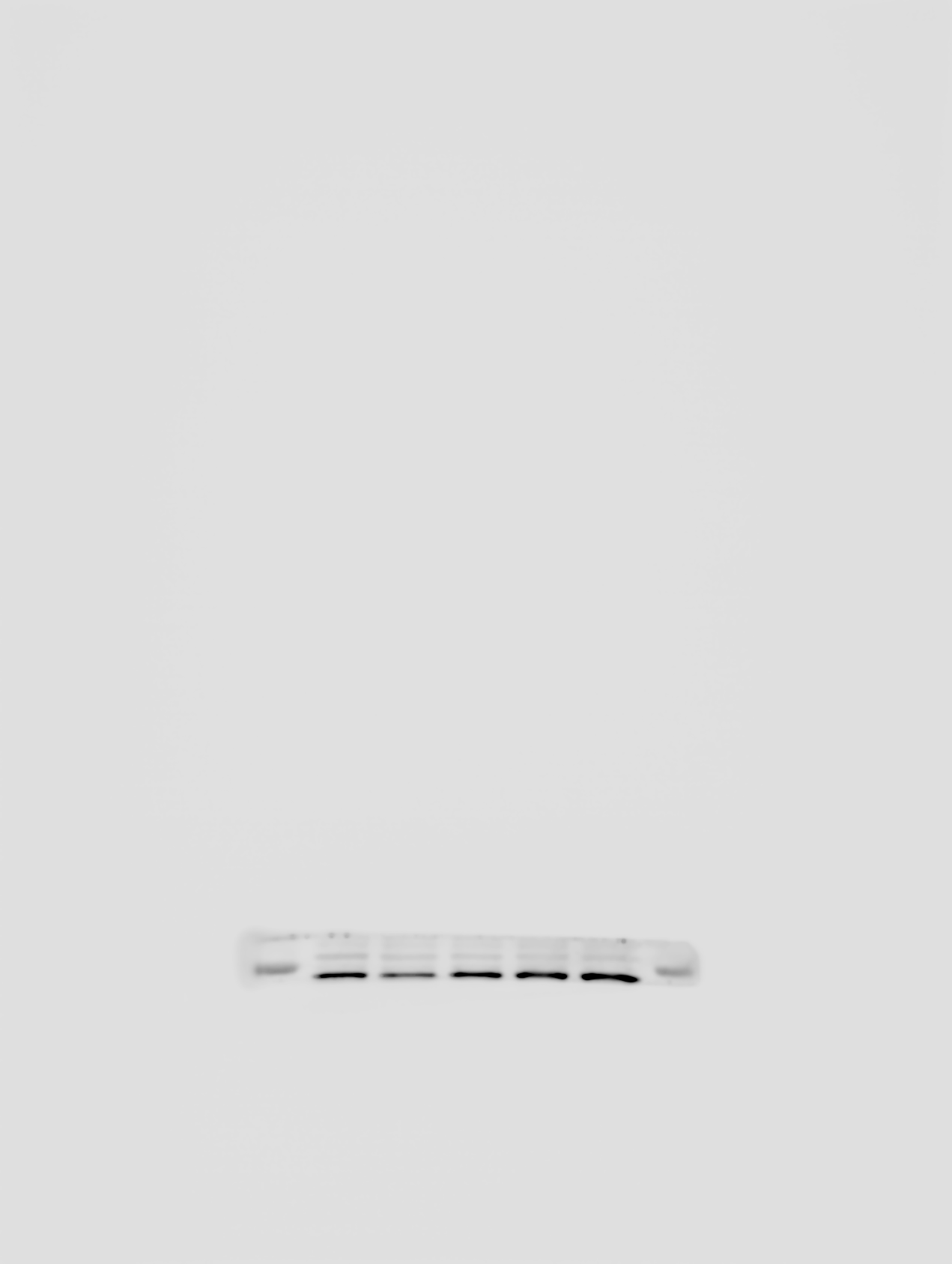

Supplement: Supplementary file 1 [file DataSheet1.zip › Raw data/WB data/Pictures/P-P38/P-P38-1-sample.png]

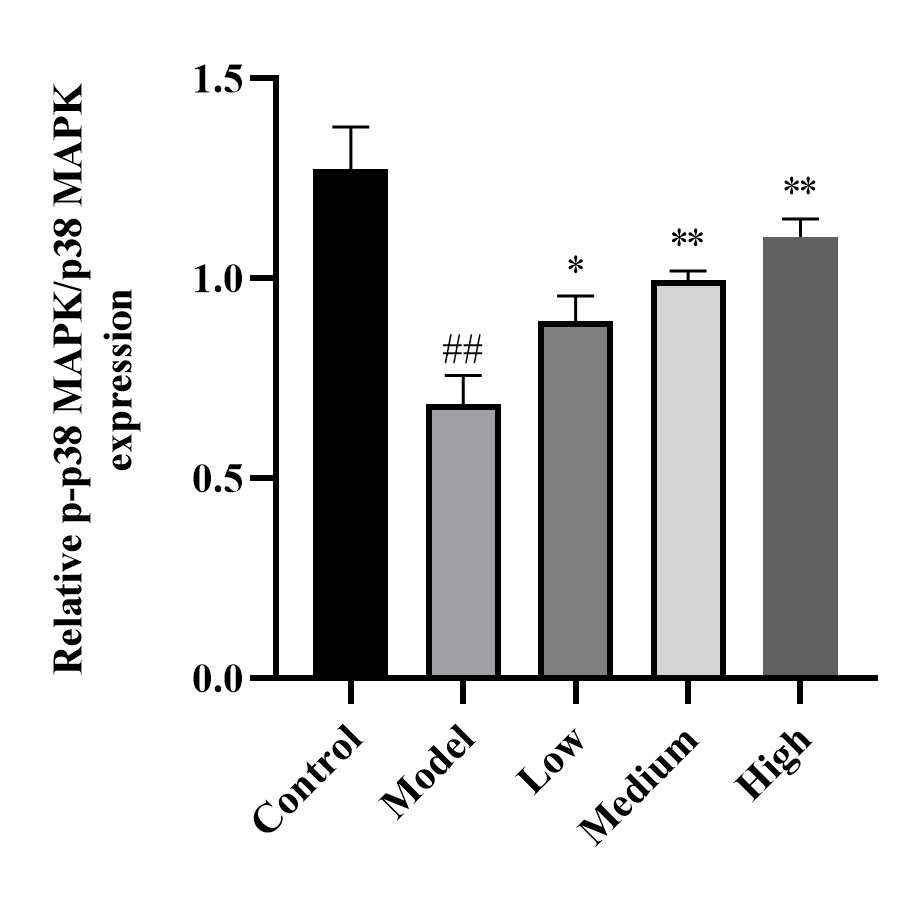

Supplement: Supplementary file 1 [file DataSheet1.zip › Raw data/WB data/Pictures/P-P38_P38.png]

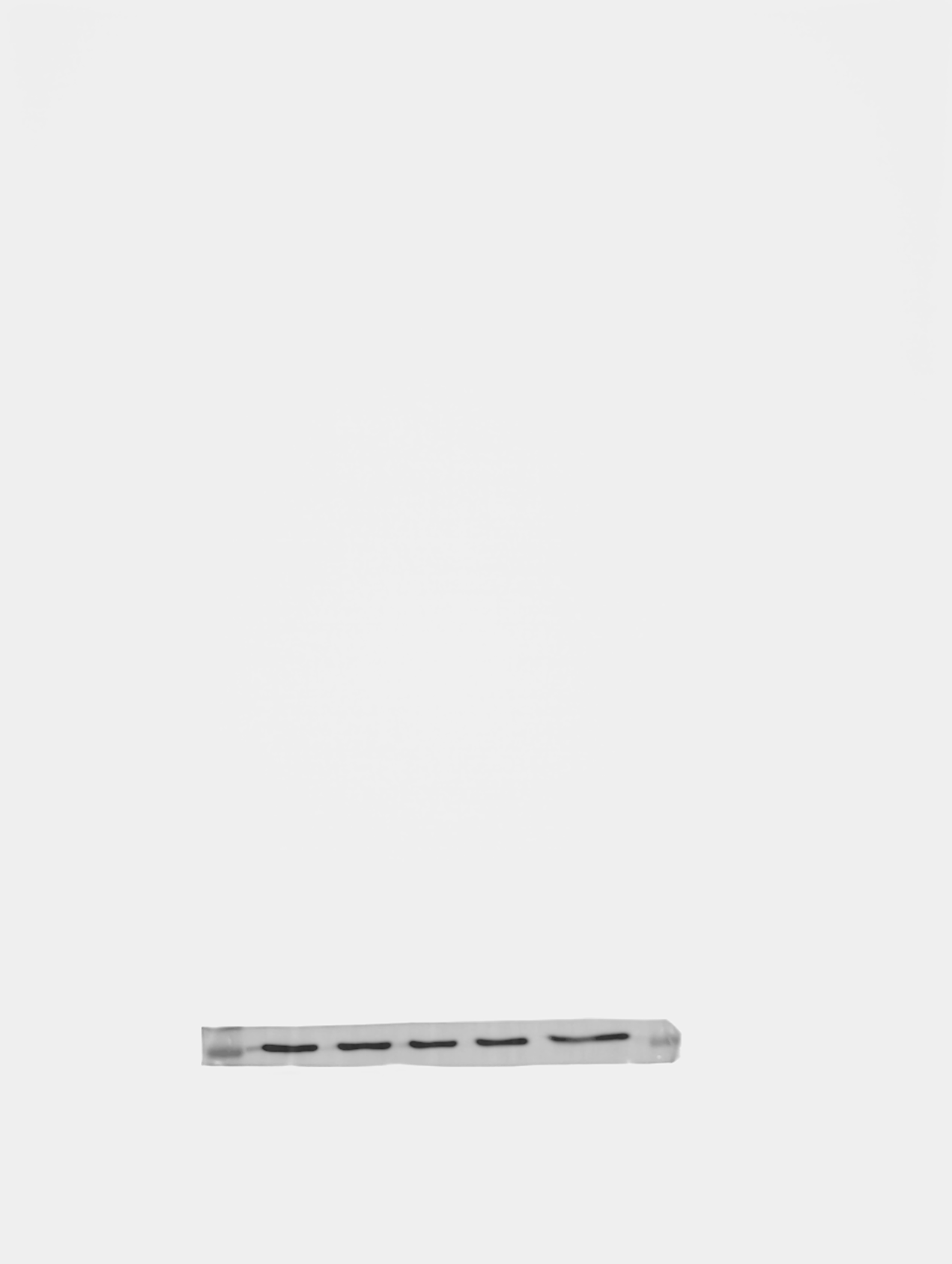

Supplement: Supplementary file 1 [file DataSheet1.zip › Raw data/WB data/Pictures/P38/GAPDH-4+M.png]

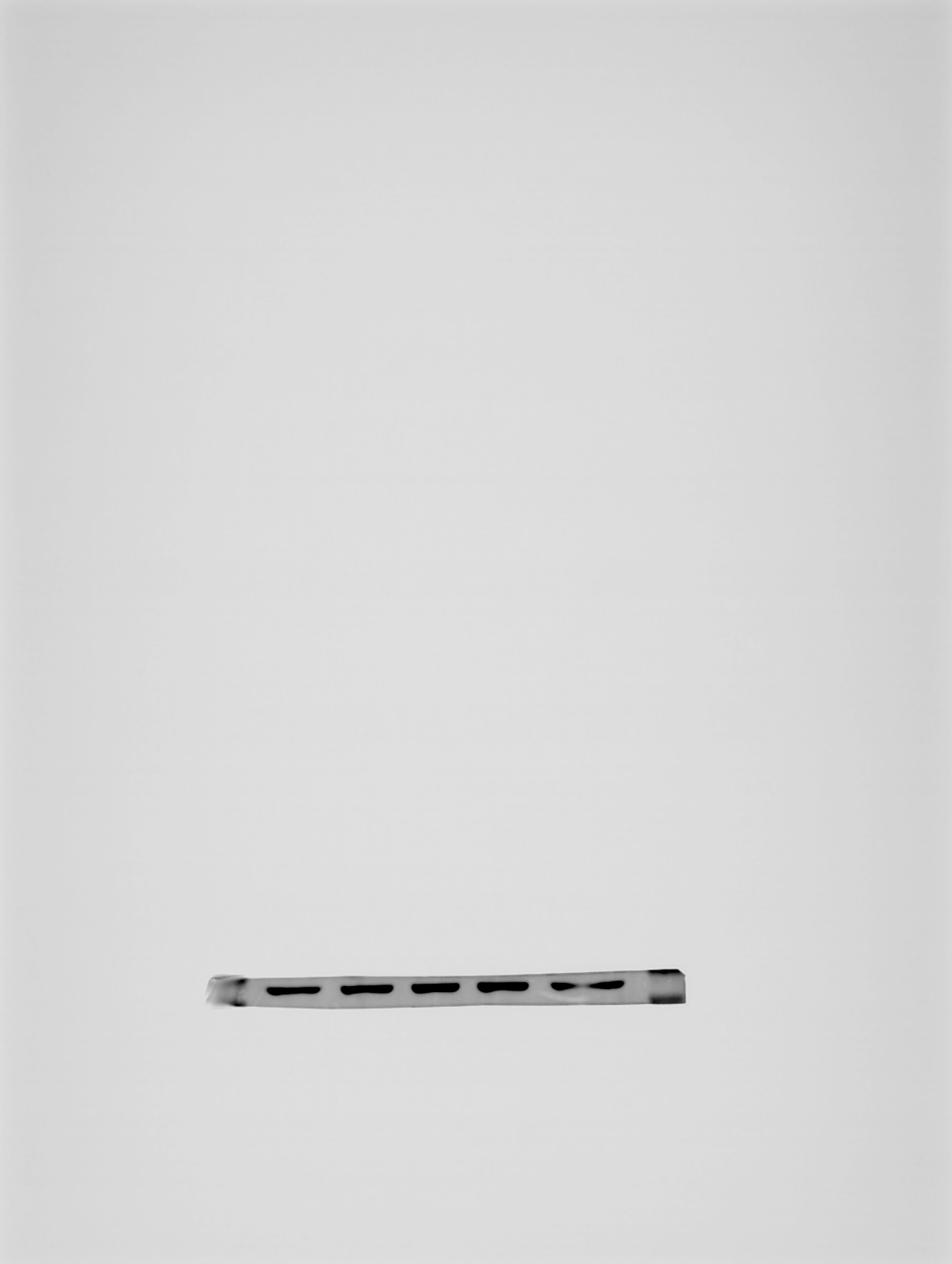

Supplement: Supplementary file 1 [file DataSheet1.zip › Raw data/WB data/Pictures/P38/P38-4+M.png]

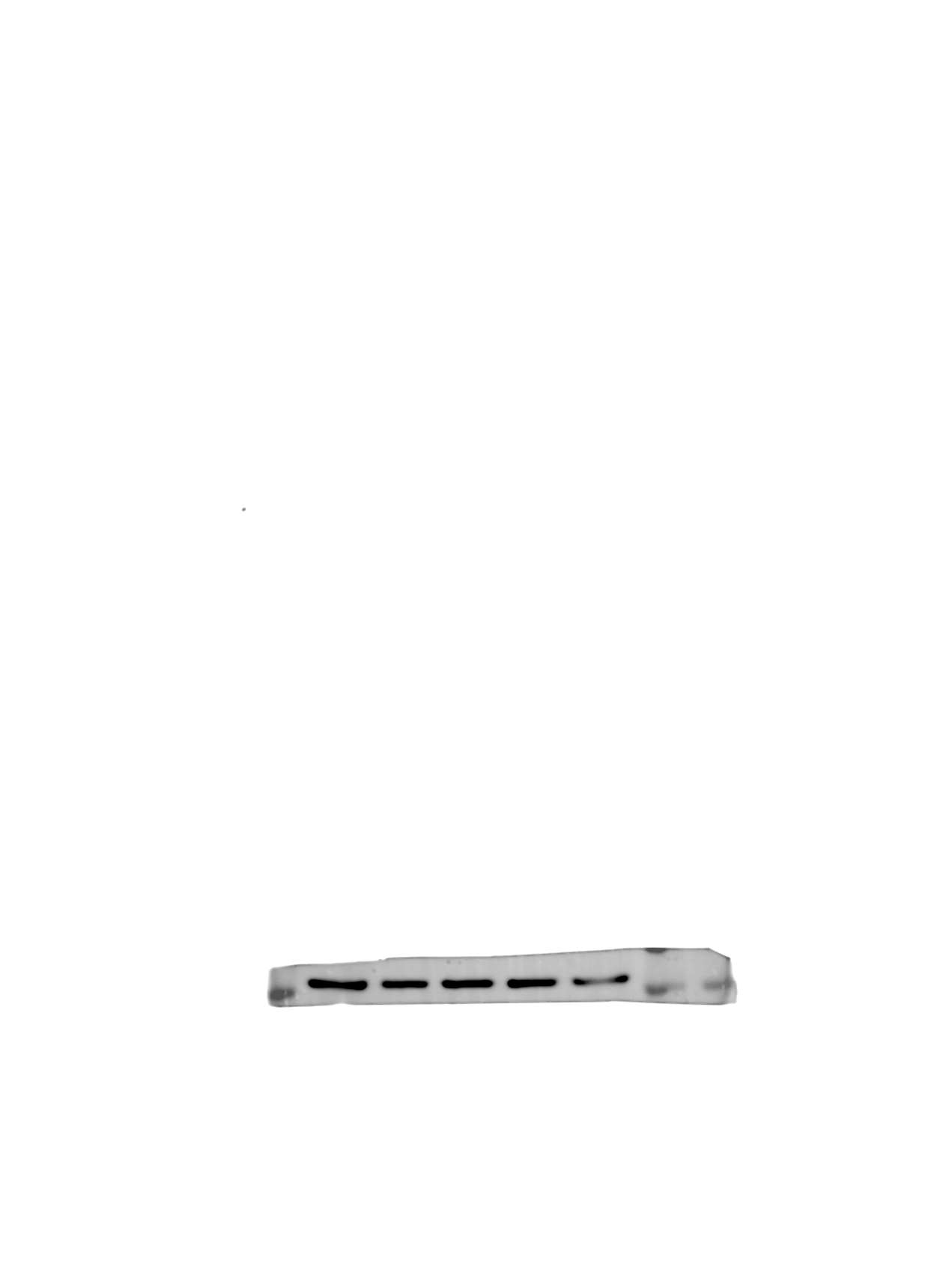

Supplement: Supplementary file 1 [file DataSheet1.zip › Raw data/WB data/Pictures/SCL3A2/GAPDH+M.png]

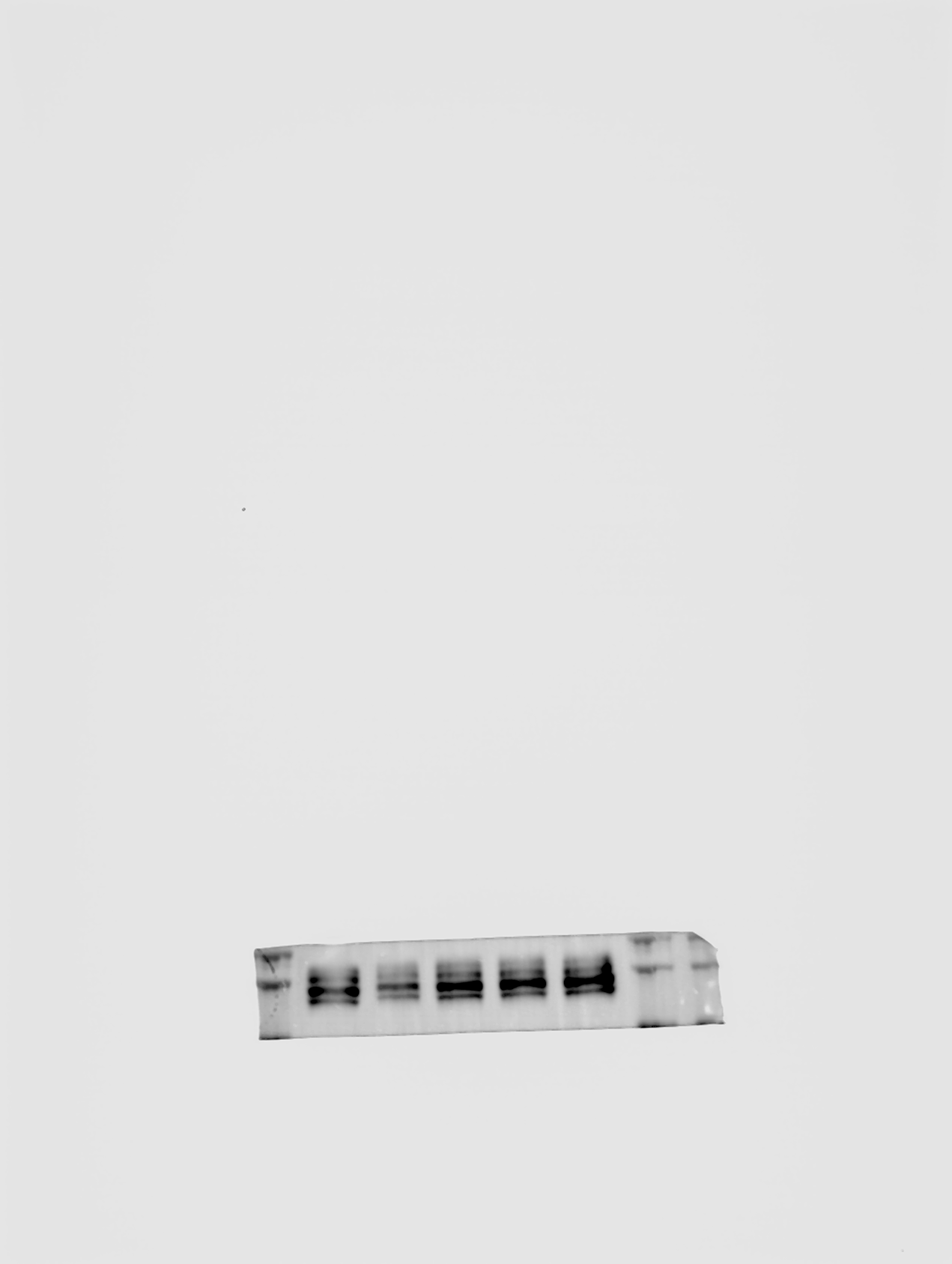

Supplement: Supplementary file 1 [file DataSheet1.zip › Raw data/WB data/Pictures/SCL3A2/SCL3A2+M.png]

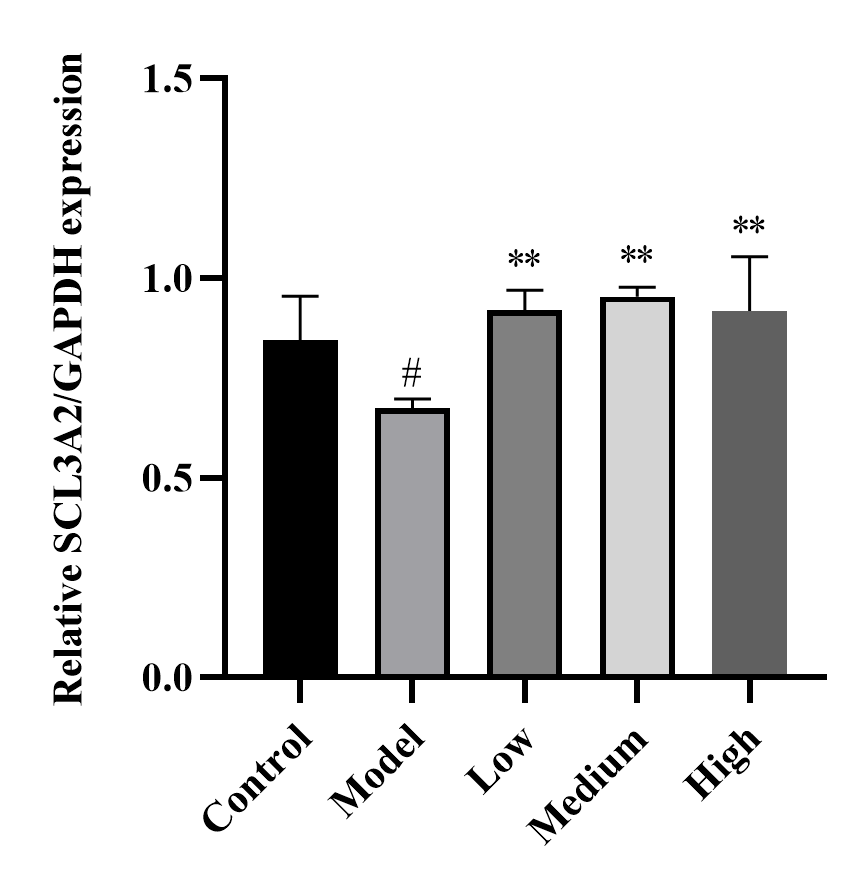

Supplement: Supplementary file 1 [file DataSheet1.zip › Raw data/WB data/Pictures/SCL3A2.png]

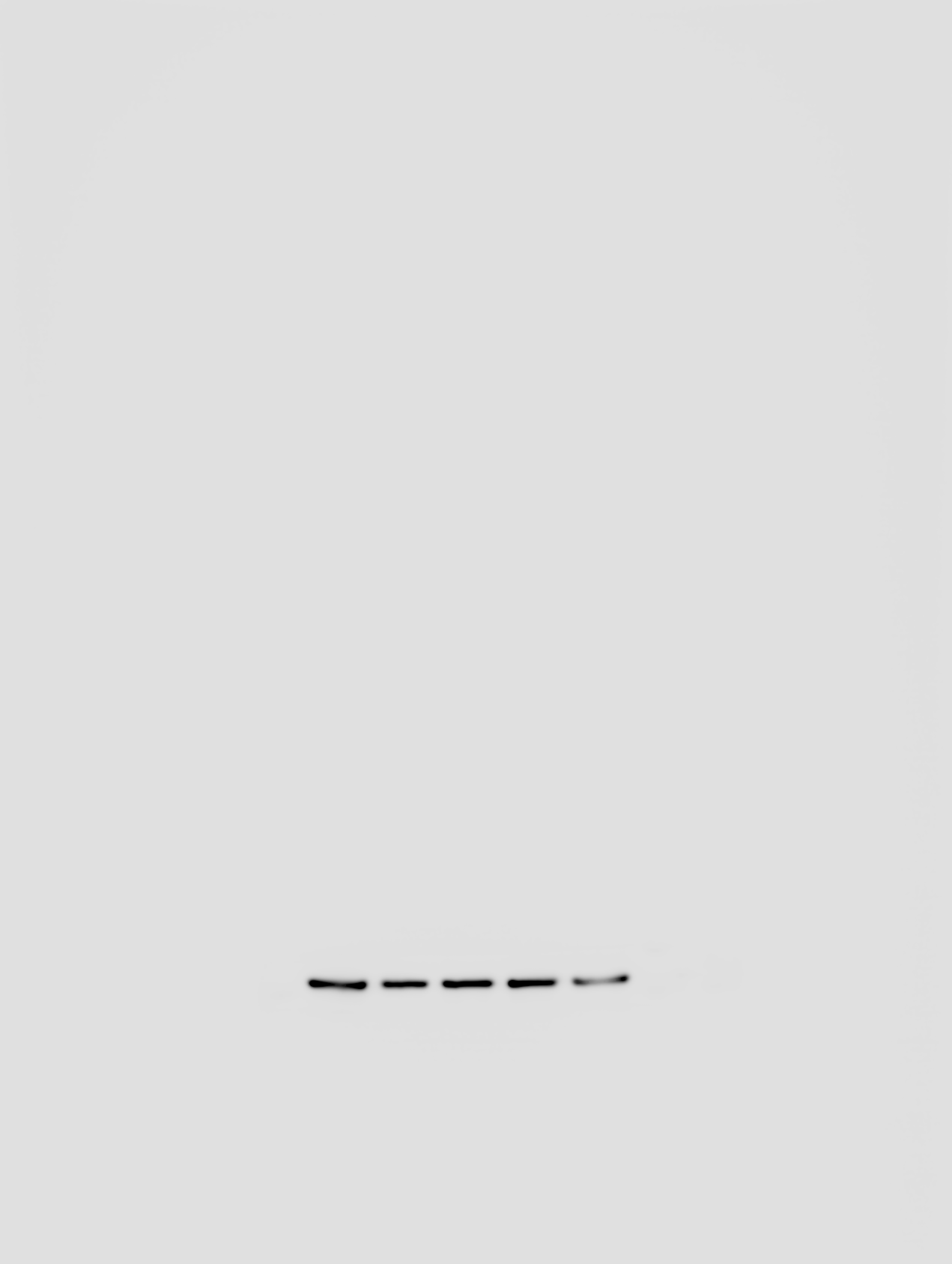

Supplement: Supplementary file 1 [file DataSheet1.zip › Raw data/WB data/Pictures/XCT/GAPDH+M-sample.png]

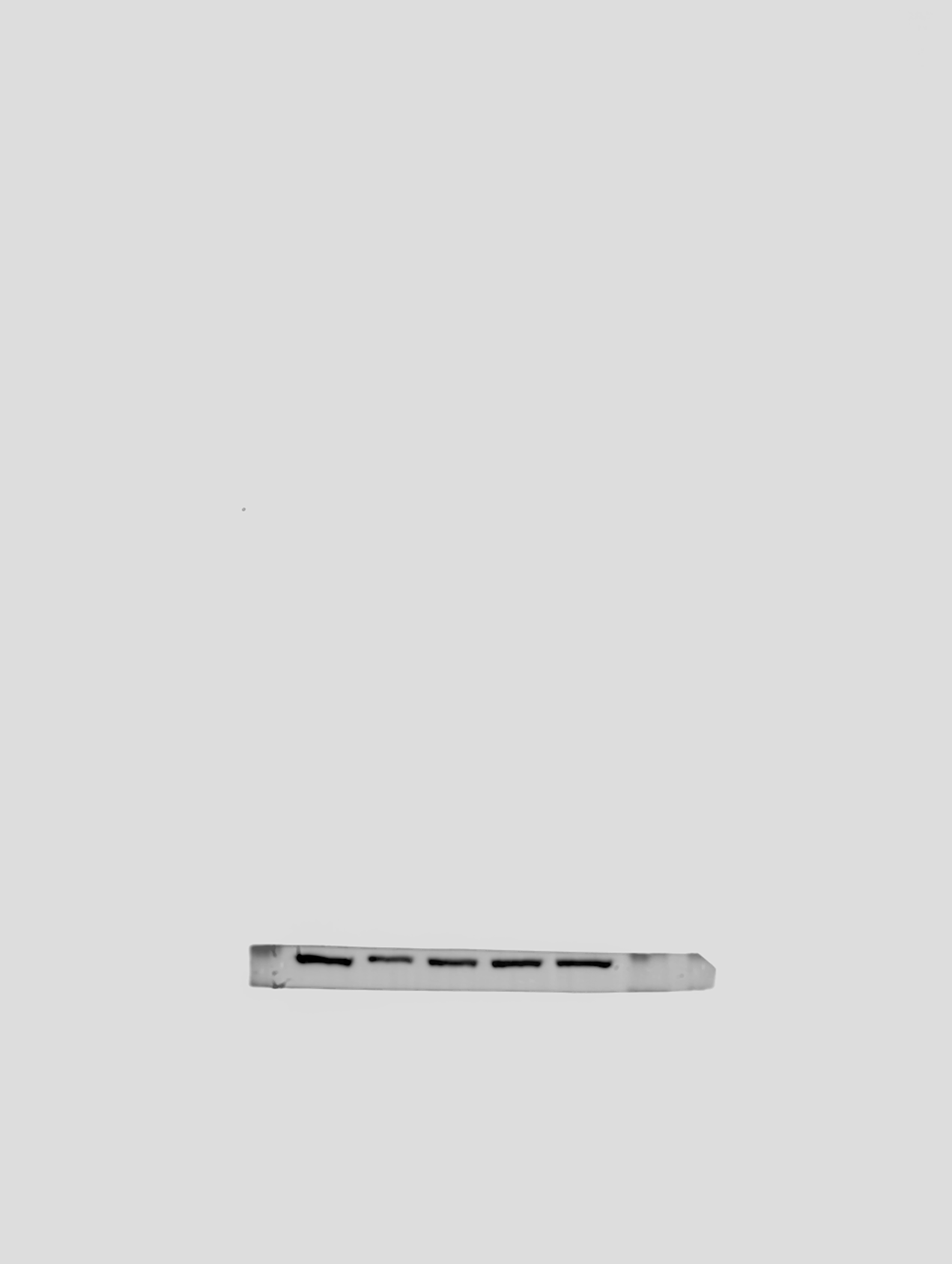

Supplement: Supplementary file 1 [file DataSheet1.zip › Raw data/WB data/Pictures/XCT/XCT+M.png]

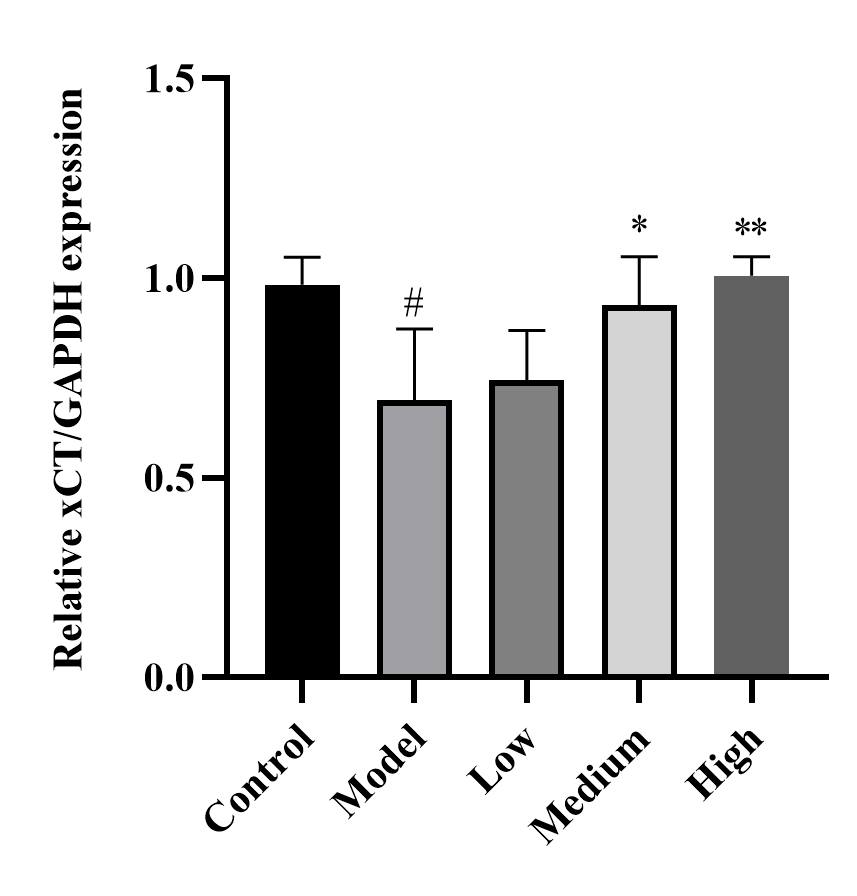

Supplement: Supplementary file 1 [file DataSheet1.zip › Raw data/WB data/Pictures/xCT.png]
